# Evidence supportive of a bacterial component in the etiology for Alzheimer’s disease and for a temporal-spatial development of a pathogenic microbiome in the brain

**DOI:** 10.3389/fcimb.2023.1123228

**Published:** 2023-09-13

**Authors:** Yves Moné, Joshua P. Earl, Jarosław E. Król, Azad Ahmed, Bhaswati Sen, Garth D. Ehrlich, Jeffrey R. Lapides

**Affiliations:** Department of Microbiology and Immunology, Centers for Genomic Sciences and Advanced Microbial Processing, Drexel University College of Medicine, Philadelphia, PA, United States

**Keywords:** Alzheimer’s disease, 16s sequencing, latent dirichlet allocation, Bayesian, microbiome, *Cutibacterium*, blood brain barrier, glymphatic network

## Abstract

**Background:**

Over the last few decades, a growing body of evidence has suggested a role for various infectious agents in Alzheimer’s disease (AD) pathogenesis. Despite diverse pathogens (virus, bacteria, fungi) being detected in AD subjects’ brains, research has focused on individual pathogens and only a few studies investigated the hypothesis of a bacterial brain microbiome. We profiled the bacterial communities present in non-demented controls and AD subjects’ brains.

**Results:**

We obtained postmortem samples from the brains of 32 individual subjects, comprising 16 AD and 16 control age-matched subjects with a total of 130 samples from the frontal and temporal lobes and the entorhinal cortex. We used full-length 16S rRNA gene amplification with Pacific Biosciences sequencing technology to identify bacteria. We detected bacteria in the brains of both cohorts with the principal bacteria comprising *Cutibacterium acnes* (formerly *Propionibacterium acnes*) and two species each of *Acinetobacter* and *Comamonas* genera. We used a hierarchical Bayesian method to detect differences in relative abundance among AD and control groups. Because of large abundance variances, we also employed a new analysis approach based on the Latent Dirichlet Allocation algorithm, used in computational linguistics. This allowed us to identify five sample classes, each revealing a different microbiota. Assuming that samples represented infections that began at different times, we ordered these classes in time, finding that the last class exclusively explained the existence or non-existence of AD.

**Conclusions:**

The AD-related pathogenicity of the brain microbiome seems to be based on a complex polymicrobial dynamic. The time ordering revealed a rise and fall of the abundance of *C. acnes* with pathogenicity occurring for an off-peak abundance level in association with at least one other bacterium from a set of genera that included *Methylobacterium*, *Bacillus*, *Caulobacter*, *Delftia*, and *Variovorax*. *C. acnes* may also be involved with outcompeting the *Comamonas* species, which were strongly associated with non-demented brain microbiota, whose early destruction could be the first stage of disease. Our results are also consistent with a leaky blood–brain barrier or lymphatic network that allows bacteria, viruses, fungi, or other pathogens to enter the brain.

## Introduction

More than a century ago, Oskar Fischer (Fischer, 1907; Fischer, 1910) and then Alois Alzheimer (Alzheimer, 1907) independently described the two histopathological hallmarks of the neurodegenerative disorder which is now called Alzheimer’s disease: amyloid-β (Aβ) plaques and neurofibrillary tangles (NFT) (Goedert, 2009). Alzheimer’s disease (AD) is the most common form of dementia in the elderly, accounting for an estimated 60 to 80% of cases of dementia. Worldwide, an estimated 55 million people are living with dementia and this number is expected to reach near 80 million in 2030 and 140 million in 2050. In 2019, the World Health Organization (WHO) estimated the global cost of dementia to be 1.3 trillion US$ and it is projected that by 2030 this cost will increase to almost 2.8 trillion US$ (World Health Organization, 2021). AD patients are affected by memory loss and a progressive decline of cognitive abilities (thinking, language, behavior changes) (2020 Alzheimer’s disease facts and figures, 2020). The majority of AD cases are sporadic, late-onset forms of the disease occurring after the age of 65 years, and only a small percentage of cases (around 5%), mostly familial, presenting earlier (Mendez, 2017).

AD is characterized by neuroinflammation, extracellular deposition of Aβ peptides into plaques in the brain parenchyma, and intraneuronal NFT, composed of hyperphosphorylated tau (ptau), which ultimately lead to a loss of synapses and neurons. Aβ deposition has been considered as the main cause of the disease leading to the “amyloid cascade hypothesis” as a model of AD pathogenesis (Hardy and Allsop, 1991; Hardy and Selkoe, 2002; Selkoe and Hardy, 2016). Aβ peptides are produced through the abnormal processing of the Aβ precursor protein (APP) by the sequential action of β- and γ-secretases. This amyloidogenic processing produces Aβ peptides differing in length, including the highly pathogenic and aggregation-prone Aβ42 (42 amino acids) and the less neurotoxic Aβ40 (40 amino acids) (Gu and Guo, 2013; Bolduc et al., 2016; Terrill-Usery et al., 2016; Dunys et al., 2018). Aβ peptides aggregate into oligomers, fibrils, and plaques in the extracellular space. Aβ is also involved in the formation of NFT by induction of hyperphosphorylation of the tau protein (a microtubule‐ associated protein) via the kinase Fyn (Larson et al., 2012; Li and Götz, 2017; Nisbet and Götz, 2018; Vergara et al., 2019).

For the last several decades, the amyloid cascade hypothesis has guided much of AD research. However, multiple observations challenge this model. First, the amyloid cascade hypothesis is based on the study of the genetic mutations observed in the rare early onset forms of AD, and clinical trials targeting Aβ accumulation have not resulted broadly accepted treatment (Kim et al., 2022). Moreover, the quantitative level of Aβ does not correlate with the amount of cognitive decline and a substantial proportion of healthy elderly subjects (10%–30%) show significant amyloid deposition (Cohen et al., 2012; Higashi et al., 2018; Osorio et al., 2019) (see Selkoe and Hardy (Selkoe and Hardy, 2016) for counter arguments). Although Aβ and ptau pathologies remain essential markers of the disease, the aforementioned observations suggest that the amyloid cascade hypothesis does not address satisfactorily the causality of AD and urge to investigate alternative explanatory models (Lathe et al., 2023).

In recent years, a growing body of evidence has suggested a role for various microorganisms (virus, bacteria, fungi) as well as the innate immune system and neuroinflammatory pathways in AD pathogenesis, leading to the emergence of alternative models variously called the “pathogen hypothesis” (or “infectious hypothesis”) and “antimicrobial protection hypothesis” (Itzhaki et al., 2016; Sochocka et al., 2017; Moir et al., 2018; Fülöp et al., 2020; Itzhaki et al., 2020). Diverse pathogens have been detected in the brains of AD patients. Viruses, particularly from the Herpesviridae family, have long been suspected to play a role in AD (Terrill-Usery et al., 2016; Itzhaki, 2018). Herpes simplex virus type 1 (HSV1) has been found to be active in brains from non-demented elderly as well as in AD patients and to be localized within amyloid plaques (Wozniak et al., 2009). A retrospective cohort study from Taiwan showed that subjects with HSV infections may have a 2.56-fold increased risk of developing dementia and that anti-herpetic treatment of HSV infections was associated with a decreased risk of dementia (Tzeng et al., 2018). Recent findings suggest that Herpesviridae infections could contribute directly to amyloid deposition (Eimer et al., 2018; Ezzat et al., 2019), and it has been suggested that multiple prion-like domains found in the HSV1 proteins could trigger protein misfolding in AD (Tetz and Tetz, 2018). Nonetheless, the potential role of Human Herpesvirus 6 and 7 in AD pathogenesis (Readhead et al., 2018) remains controversial (Jeong and Liu, 2019; Readhead et al., 2019; Allnutt et al., 2020; Chorlton, 2020; Rizzo, 2020).

Another body of work has associated bacteria with an etiological role in AD pathogenesis. Although the brain is protected by a highly selective barrier called the blood–brain barrier (BBB), which regulates the exchange between blood and brain compartments, some bacteria are able to invade the brain. The bacteria could reach the central nervous system through the olfactory tract or the trigeminal nerve or as a result of a weakened BBB (Doty, 2008; Dando et al., 2014; Chacko et al., 2022; Vojtechova et al., 2022). The presence of spirochetes including the Lyme disease agent, *Borrelia burgdorferi*, and the periodontal *Treponema* spp. pathogens has been repeatedly identified in postmortem AD brains. Moreover, tertiary syphilis produces a dementia, general paresis, with a neurohistopathology complete with Aβ, NFT, and associated behavioral changes essentially identical to AD (Miklossy, 2011; Miklossy, 2016). Other bacterial species including *Chlamydia pneumoniae*, *Porphyromonas gingivalis*, and *Cutibacterium acnes* (formerly *Propionibacterium acnes*) have also been linked with AD (Little et al., 2004; Carter, 2017; Chen et al., 2017; Emery et al., 2017; Alonso et al., 2018; Al-Atrache et al., 2019; Dominy et al., 2019; Haditsch et al., 2020; Woods et al., 2020; Emery et al., 2022). *C. pneumoniae* is an intracellular respiratory bacterial pathogen that was proposed to cause sporadic late-onset AD (Woods et al., 2020). *In vitro* studies have shown that *C. pneumoniae* is able to infect human astrocytes and to promote amyloidogenic APP processing (Al-Atrache et al., 2019) and murine models of *C. pneumoniae* CNS infection have recapitulated the cardinal features of AD (Little et al., 2004). Epidemiological studies suggest a relationship between periodontitis and AD (Chen et al., 2017; Emery et al., 2021). Among the periodontitis-related pathogens, *P. gingivalis* is a keystone pathogen for both chronic periodontitis and systemic sequelae. Dominy et al. have detected *P. gingivalis* DNA and gingipains (arginine- or lysine-specific cysteine proteases and major virulence factors in *P. gingivalis*) in postmortem AD brains and in the cerebrospinal fluid (CSF) of living AD patients (Dominy et al., 2019). Moreover, a recent *in vitro* study by Haditsch et al. has shown the neurotoxicity of the gingipains and that *P. gingivalis* can invade and persist in neurons. The infected neurons display AD-like neuropathology including an increase in tau phosphorylation ratio (Haditsch et al., 2020). In addition, other bacterial factors have been suggested to be involved in AD pathology such as lipopolysaccharides (LPS) from Gram-negative bacteria, which can induce a neuroinflammation (Zhan et al., 2018), bacterial extracellular DNA which may promote Aβ and tau aggregation (Tetz et al., 2020; Tetz and Tetz, 2021), or microbial amyloid proteins, which could trigger the propagation of misfolded endogenous proteins in a prion-like manner and enhance the inflammatory response (Chen et al., 2016; Friedland and Chapman, 2017).

The vast majority of such microbial survey studies in AD have relied on molecular diagnostics in which the bacterial DNA is directly detected, by either a PCR-based method (Balin et al., 1998; Dominy et al., 2019) or *in situ* hybridization (FISH) (Miklossy, 2016)—as opposed to cultural methods owing to the demonstrated difficulty in culturing bacteria associated with chronic infections and biofilms (Post et al., 1995; Costerton et al., 2003; Ehrlich et al., 2005; Ehrlich et al., 2010; Stoodley et al., 2011; Ehrlich et al., 2012) and the greatly improved sensitivity and specificity of nucleic acid-based methods (Post et al., 1996; Aul et al., 1998; Dingman et al., 1998). Most recently, species-specific, pan-domain molecular diagnostics have become available for bacteria (Tuttle et al., 2011; Nickel et al., 2015; Nickel et al., 2016; Earl et al., 2018; Socarras et al., 2021). These assays provide for unbiased surveys without the need for investigators to *a priori* decide what taxa to survey. Preliminary microbiome studies using next-generation sequencing of the variable regions of 16S ribosomal rRNA gene (V3, V4) have also identified several bacterial species in both AD brains and non-demented controls (Emery et al., 2017; Westfall et al., 2020). Emery et al. have found higher bacterial loads in AD brains with a higher proportion of Actinobacteria, especially *C. acnes* (Emery et al., 2017; Emery et al., 2022), whereas the study of Westfall et al. showed no difference in bacterial populations between AD and control subjects but variations in microbial composition between hippocampal and cerebellum regions in AD subjects’ brains (Westfall et al., 2020). Microbiome studies have also detected several fungal genera as being more prevalent in AD brains (*Alternaria* spp., *Botrytis* spp., *Candida* spp., and *Malassezia* spp.) (Alonso et al., 2018).

The potential involvement of microbes as etiological agents of AD has been strengthened by the evidence that the Aβ peptide has potent antimicrobial properties. Soscia et al. demonstrated *in vitro* that the Aβ peptide possessed antimicrobial properties (Soscia et al., 2010). The antimicrobial activity of Aβ is comparable to the well-known human antimicrobial peptide (AMP) LL-37. The protective effect of Aβ against bacterial infection has been shown in a murine model where it was demonstrated to mediate entrapment of microbes by oligomerization and fibrillization of Aβ (Kumar et al., 2016). The demonstration that Aβ is an AMP has led to the antimicrobial protection hypothesis. In this model, Aβ deposition is a defensive mechanism against infection and AD pathology results from a chronic innate immune inflammatory response to a recalcitrant bacterial biofilm leading to the accumulation of Aβ deposits and ultimately mediating neurodegeneration.

In this study, we take advantage of the Pacific Biosciences (PacBio) long-read DNA sequencing technology to sequence the full-length bacterial 16S rRNA gene (Earl et al., 2018; Greathouse et al., 2018; Socarras et al., 2021) and to profile the bacterial communities to the species level in AD-affected and non-demented age-matched brains.

## Materials and methods

### Biological material and sequencing

#### Brain tissue samples

Frozen postmortem human brain samples were obtained from the University of Arkansas for Medical Sciences (UAMS). All the samples were neuropathologically evaluated by the provider. All Alzheimer’s disease cases were given Braak stages IV–VI. The control cases designated as age-matched controls (controls) were described as non-demented. The average postmortem interval was 8 h. The data contained 130 samples from 32 individual subjects about half of whom had Alzheimer’s disease (“AD”). For most subjects, we had at least one sample from the entorhinal cortex and the frontal and temporal lobes. We had no underlying histological information from the sample sites with regard to AD diagnoses.

To minimize any risk of environmental contamination of the brain autopsy specimens, all specimens upon receipt were opened in a BSL2+ laminar flow hood with proper personal protective equipment (lab coat, mask, gloves, and protective eyewear). Then, a cortical piece of each specimen was dissected using sterile techniques such that none of the specimen used for DNA extraction had ever been in touch with a non-sterile surface. The control of contamination was also addressed analytically in the downstream statistical analyses (see below).

#### DNA extraction

Total DNA was isolated from frozen brain biopsies using the DNeasy Blood and Tissue Kit (Qiagen) according to the manufacturer’s recommendations with slight modifications. The biopsy material was incubated overnight at 56°C with 570 μl ATL tissue lysis buffer with 30 μl Proteinase K in a Lysing Matrix E tube (MP Biomedicals LLC), homogenized by SPEX 1600 MiniG (SPEX SamplePrep) for 10 min at 1500 Hz, and centrifuged for 1 min at 13,000 rpm. DNA was eluted with a 200-μl AE elution buffer. DNA quality and quantity were analyzed by agarose gel electrophoresis and using a NanoDrop 2000 spectrophotometer (Thermo Fisher Scientific), respectively.

#### Full-length 16S rRNA gene amplification

The taxonomic composition of bacterial communities in the postmortem human brain tissues were analyzed using the Pacific Biosciences (PacBio) single-molecule real-time (SMRT) sequencing technology (Pacific Biosciences, Menlo Park, CA, USA) to obtain the full-length 16S ribosomal RNA (rRNA) gene sequences as previously described (Earl et al., 2018; Greathouse et al., 2018; Socarras et al., 2021). Briefly, the full-length 16S rRNA gene was amplified using the universal 16S rRNA bacterial primers 27 F (5′-GRAGAGTTTGATYMTGGCTCA) and 1492 R (5′-TACGGYTACCTTGTTACGACTT). Both the forward and reverse 16S primers were tailed with the universal sequences (5′-GCAGTCGAACATGTAGCTGACTCAGGTCAC and 5′-TGGATCACTTGTGCAAGCATCACATCGTAG, respectively) to allow for multiplexed sequencing, and a 5′ block (5′NH2-C6) was added according to the recommendations of Pacific Biosciences. The primers were synthesized and HPLC purified by Integrated DNA Technologies.

Barcoded 16S rRNA amplicons were obtained via a two-step PCR. All the PCR reactions were performed in 96-well plates. The first PCR round was performed using 10 μl of total DNA (approximately 1–2 µg of DNA) as template, the universal 16S rRNA bacterial primers 27F and 1492R described above (0.2 µM each), and 1× Hot Start GoTaq DNA Polymerase Master Mix (Promega) in a 50-µl final volume. Cycling conditions were 94°C, 3 min; then 35 cycles of 94°C 30 s, 54°C 30 s, 72°C 2 min, and a final extension step at 72°C for 5 min. The amplified products were then analyzed by agarose gel electrophoresis to check the quality and size of the amplicons. The second PCR round was performed in a 50-µl reaction volume containing 2 µl of a unique primer pair of Barcoded Universal F/R Primers (Pacific Biosciences, 100-466-100), 10 µl of 16S rRNA amplicons from each sample, and 1× Hot Start GoTaq DNA Polymerase Master Mix (Promega). Cycling conditions were 94°C, 3 min; then 20 cycles of 94°C 15 s, 64°C 15 s, 72°C 2 min, and a final extension step at 72°C for 5 min. PCR products were cleaned with AxyPrep MAG PCR (Axygen) according to the manufacturer’s protocol with a volume ratio (bead suspension to PCR product) of 2:1 and eluted in 50 μl of water. Cleaned barcoded 16S rRNA amplicons were quantified using AccuClear Ultra High Sensitivity dsDNA Quantitation Kit (Biotium) on BioTek™ FLx800™ Microplate Fluorescence Reader. Based on quantification results, barcoded amplicons were then pooled in equimolar concentration into multiplexed sets of 2 to 18 samples per pool.

#### Pacific Biosciences Sequel System sequencing

Sequencing libraries were constructed from each pool of barcoded amplicons using the SMRTbell Express Template Prep 1.0 kit (Pacific Biosciences, 100-259-100) according to the manufacturer’s instructions (Amplification of Full-Length 16S Gene with Barcoded Primers for Multiplexed SMRTbell^®^). Multiple SMRTbell libraries were then multiplex sequenced in a single SMRT Cell 1M on a Pacific Biosciences Sequel System.

#### Generation of demultiplexed CCS reads

The raw subreads generated by the sequel sequencing run were converted into circular consensus (CCS) reads and demultiplexed using the command-line version of the Pacific Biosciences’ workflow engine pbsmrtpipe (v1.3.3) or pbcromwell (1.2.5) within the SMRT Link v7 or SMRT Link v9 software, respectively. CCS reads were generated using the following parameters: minimum number of passes = 3, minimum predicted accuracy = 0.99, minimum subread length = 1,000. CCS reads were then demultiplexed by their barcode into FASTQ files.

#### OTU clustering and taxonomic classification

Full-length 16S (FL16S) sequences were then clustered into Operational Taxonomic Units (OTUs) and assigned to species taxonomic level using the Microbiome Classification by the Single Molecule Real-time Sequencing (MCSMRT) pipeline designed by Earl et al. (Earl et al., 2018). Briefly, the MCSMRT pipeline was specifically built to (i) process PacBio CCS reads (hereafter reads), (ii) construct a set of OTU representative sequences using a 3% centroid-based divergence level, (iii) assign taxonomy and confidence values at each taxonomic level to OTUs, and (iv) quantify the abundance of each OTU in each sample by counting the number of reads that aligned to each representative “centroid” OTU sequence. In other words, each read is assigned to a centroid OTU with a maximum of 3% divergence for a hit to be counted.

#### Compositional structure of microbiome data

Microbiome sequencing data are count data, i.e., the number of DNA sequence reads of each OTU detected in each sample. However, the total number counts is not informative *per se* because it is constrained by the capacity of the sequencing instrument, which can only sequence a fixed number of DNA fragments. Consequently, (i) the read counts cannot be related to the absolute number of molecules in the input sample and (ii) read counts only carry a relative information reflecting the underlying proportions of the OTUs in the sample. That is why microbiome sequencing data are referred to as compositional data: the total number of reads are constrained to a biologically irrelevant constant sum, only providing information on the relative abundance of OTUs, and any variation (increase or decrease) in the read count of one OTU led to a change in the relative abundance of other OTUs in the sample (Fernandes et al., 2014; Gloor and Reid, 2016; Gloor et al., 2017).

### Analytical methodologies

#### Introductory comments

##### Analysis models

Our focus for the data analyses was to find one or more of the following patterns in the data: (1) individual microbes which were either correlated or anti-correlated with AD, or (2) combinations of microbes that were correlated or anti-correlated with AD, given the number of bacteria observed. In other words, we were not interested in not only whether a bacterium was intrinsically pathogenic but also whether pathogenicity derived from a polymicrobial interaction acting within or between ecosystems.

We took different analytical approaches to address these types of patterns. Despite their differences, any similar findings between the two methods can provide mutual support for their respective findings and serve as strong evidence that the findings were inherently reproducible.

To find relationships between individual bacterial species and AD, we first investigated the differences in relative abundance of individual taxa between AD and control groups. We used a hierarchical Bayesian modeling approach based on a Dirichlet-multinomial model (DMM) (Harrison et al., 2020). This procedure was supervised as it used information about whether or not the samples came from AD subjects. Its results showed which individual bacteria are associated with AD.

To find relationships between combinations of bacterial species and AD that could be utilized as evidence supportive of a bacterial component in AD etiology, we used an approach, called Latent Dirichlet Allocation (LDA) (Blei et al., 2003; Griffiths and Steyvers, 2004), that first found relationships among the bacteria without using information about the disease state of the subject at all, i.e., it was unsupervised. This approach enabled us to group the bacteria and their abundances into different subsets of bacteria and their abundances, called classes, and then relate the classes to AD.

##### Statistical properties of the data

The brain microbiome data are characterized by sample heterogeneity, huge variance in counts (overdispersion), sparseness, and compositionality (see **Supplement Figure SF1**). These characteristics are often observed in microbiome data (Weiss et al., 2017; Lutz et al., 2022) and are very challenging for the statistical analysis, which explain our choice of particular analytical approaches. **Table 1** shows the top 30 genera ordered by their abundances in the data set. The list contains both species and genera where we have broken out species for several high abundance genera. In this paper, we will often refer to *Cutibacterium*, *Acinetobacter*, and *Comamonas* as the principal bacteria mainly because of their overall abundance and prevalence, but in the case of *Comamonas*, because of its abundance and prevalence within a single class not associated with AD.

**Table 1:** Top 30 genera/species by prevalence in order top to bottom, left to right.

	
*Cutibacterium acnes*	Achromobacter
*Acinetobacter junii*	Sphingomonas
*Acinetobacter tjernbergiae*	Anabaena
*Comamonas jiangduensis*	Variovorax
*Comamonas testosteroni*	Bacillus
*Nitrosospira*	Streptococcus
*Acidovorax*	Gemella
*Delftia*	Bosea
*Sediminibacterium*	Stenotrophomonas
*Cloacibacterium*	Ferrovibrio
*Bradyrhizobium*	Bacteroides
*Pseudomonas*	Janthinobacterium
*Methylobacterium*	Brevundimonas
*Kocuria*	Corynebacterium
*Moraxella*	Massilia

###### High-abundance resolution view

We show a couple of comparisons of the abundance distributions for two of these in AD and control samples in **Figure SF1**. While there is a hint of difference in the average abundances between the AD samples and the controls, the wide variances apparent in the figure render the differences statistically insignificant. This pattern is similar for all of the bacterial species that occur frequently in the samples.

Another typical characteristic that we observed was the sparseness of the data, meaning that most of the observed bacteria do not occur in most of the samples and if they do, they do not have the same abundance. This could mean that the bacteria have little to do with AD or that behavioral redundancies across bacteria must be discovered to reveal bacterial pathogenicity.

A number of bacteria have high abundances only in a few samples, e.g., *Methylobacterium*. Using standard arguments, we could have chosen to filter these out because of their low occurrence, but it is hard to dismiss these bacteria because they have high abundance and, generally speaking, high abundance is more likely causal than low abundance. We considered that these were contaminants but eventually found that together they exhibited patterns that could be a critical factor in the etiology of AD.

###### Low-abundance resolution discrete view

In order to get a better sense for the data and potential biologically meaningful patterns it harbored, we decided to generate a view of the data with greatly reduced abundance resolution. Mindful of the possibility that some bacteria of low abundance may have a disproportionate effect on pathogenicity, we chose to logarithmically bin the data abundances. Moreover, it provided a simple way to compare differences between the cohorts within an abundance range.

Specifically, we defined a set of contiguous abundance bins in the 0.0% to 100.0% range and labeled them with integers. The bin sizes which we chose are shown in **Table 2**. We then mapped the abundance data into descriptive discrete objects formed by appending the numerical bin label to the microbe name, e.g., *Cutibacterium acnes*-14. The result of the binning was to transform a row of abundance data from a table whose rows correspond to samples and whose columns correspond to microbe name into a list of microbial objects. We will sometimes refer to these objects as measurements or measurement objects.

**Table 2:** Abundance bins in percent.

Bin	Normalized abundance
1	0.00E+00 – 3.16E−05
2	3.16E−05 – 1.00E−04
3	1.00E−04 – 3.16E−04
4	3.16E−04 – 1.00E−03
5	1.00E−03 – 3.16E−03
6	3.16E−03 – 0.010
7	0.010 – 0.0316
8	0.0316 – 0.100
9	0.100 – 0.316
10	0.316 – 1.00
11	1.00 – 3.16
12	3.16 – 10.00
13	10.00 – 31.62
14	31.62 – 100.0

In **Table 3**, we compare the occurrence of these objects in the AD and control cohorts. Note the correlations with AD among certain objects, in particular *Cutibacterium acnes*-13, *Acinetobacter junii*-13, and *Acinetobacter tjernbergiae*-13. It is important to note that these are not the maximum abundances nor close to 100% abundance, indicating the presence of other microbes in the sample and potential microscopic dynamics at play. Standard statistics do not usually look at parts of distributions. They look at averages and variances of the entire distribution, so we found this intriguing. *Comamonas jiangduensis* presents a curious situation in that its objects are associated with the no-AD disease state of the controls. Last, while the statistics are weak, there is little indication that low-abundance microbial objects are correlated with disease state.

**Table 3:** Object statistics, comparison of samples from Alzheimer’s and controls subjects.

MICROBIAL OBJECT	Alzheimer’s	Controls	MICROBIAL OBJECT	Alzheimer’s	Controls
***Cutibacterium acnes*-14**	20	23	***Acidovorax*-8**	4	3
***Cutibacterium acnes*-13**	17	6	***Acinetobacter junii*-10**	2	5
***Acinetobacter junii*-13**	14	8	***Comamonas jiangduensis*-14**	0	7
***Acinetobacter tjernbergiae*-13**	11	6	***Sediminibacterium*-13**	4	2
***Acinetobacter junii*-14**	4	13	***Pseudomonas*-9**	4	2
***Cloacibacterium*-12**	9	6	***Comamonas testosteroni*-11**	4	2
***Cutibacterium acnes*-12**	9	4	***Sediminibacterium*-11**	3	3
***Acidovorax*-13**	8	4	***Streptococcus*-12**	2	4
***Acinetobacter tjernbergiae*-10**	1	10	***Nitrosospira*-13**	4	1
***Cloacibacterium*-11**	3	7	***Moraxella*-10**	4	1
***Acidovorax*-11**	3	7	***Delftia*-14**	4	1
***Cutibacterium acnes*-11**	6	3	***Acinetobacter junii*-11**	4	1
***Acinetobacter tjernbergiae*-12**	6	3	***Acidovorax*-14**	4	1
***Acinetobacter tjernbergiae*-14**	5	4	***Sediminibacterium*-10**	3	2
***Streptococcus*-11**	4	5	***Nitrosospira*-14**	3	2
***Sediminibacterium*-12**	6	2	***Bacillus*-10**	3	2
***Corynebacterium*-11**	6	2	***Acidovorax*-12**	3	2
***Comamonas testosteroni*-10**	6	2	***Streptococcus*-10**	2	3
***Delftia*-11**	5	3	***Stenotrophomonas*-10**	2	3
***Moraxella*-11**	3	5	***Cutibacterium acnes*-10**	2	3
***Corynebacterium*-10**	3	5	***Kocuria*-10**	2	3
***Bradyrhizobium*-10**	3	5	***Streptococcus*-9**	1	4
***Acinetobacter junii*-12**	3	5	***Pseudomonas*-8**	1	4
***Pseudomonas*-12**	2	6	***Pseudomonas*-11**	1	4
***Cloacibacterium*-10**	2	6	***Nitrosospira*-11**	1	4
***Novosphingobium*-9**	5	2	***Lactobacillus*-10**	1	4
***Moraxella*-12**	5	2	***Anaerococcus*-8**	1	4
***Moraxella*-9**	4	3	***Comamonas jiangduensis*-13**	0	5

The statistics quoted in the previous paragraph and in **Table 3** show how often the object came from a subject who had or did not have AD. When an object occurs more often in AD subjects, this does not necessarily mean that the bacterium and abundance it represents are pathogenic. We will show below that many of these are likely not pathogenic.

These simple observations suggested to us why crude analyses fail and that a completely different way of analyzing the data is needed.

#### Differences in individual bacterial abundances between AD and control subjects

##### Data filtering and contaminant removal

As samples vary in total read number, low-yield samples could introduce substantial noise, so the samples with less than 100 total reads were removed from the dataset. Four blank extraction controls (composed of all reagents used during sample processing but without sample input) were processed in the same way as the true biological samples to allow identification of any contamination from reagents or during sample processing. Potential contaminant OTUs were detected based on their occurrence in biological samples vs. negative controls using a prevalence-based method (IsNotContaminant function) from the R package Decontam (Davis et al., 2018). To qualify as contaminant, an OTU had to have a score ≥0.5 or a higher mean relative abundance in the negative controls than the biological samples (**Supplementary Table S1**). Contaminant OTUs were then removed from the dataset. The phyloseq R package (McMurdie and Holmes, 2013) was used for handling OTU counts, taxonomy, and sample metadata.

##### Exploratory data analyses

Exploratory data analysis has been done using compositional data analysis (CoDA) methods which are based on log-ratio transformation of the data (Gloor et al., 2017). OTU counts were normalized using the centered log-ratio (clr) transformation by taking the log of the ratio of each OTU counts to the geometric mean of all OTUs in a sample. A pseudocount of 1 was applied to zero entries in the OTU count table before taking the log to the base 2. A positive clr value for a given OTU indicates a relatively higher amount than the overall composition mean, and a negative value indicates a relatively lower amount. Clr-transformed data were used to produce a heatmap with the pheatmap R package and to perform a principal component analysis (PCA) using the prcomp function from the R Stats package (R Core Team, 2018).

##### Differences in relative abundances

To test for differences in the relative abundances of individual OTUs between AD and control sampling groups, the OTU count data were analyzed using a hierarchical Bayesian model that relies on the Dirichlet and multinomial distributions as described in Harrison et al. (2020). Briefly, these two distributions are used to construct a hierarchical model which estimates relative abundances among samples and sampling groups and enables the detection of differences in relative abundances for each OTU between AD and control groups (see **Supplementary Methods** for a full description of the model). The Dirichlet-multinomial model (DMM) is relevant for the compositional structure of microbiome data because analyses are performed on proportions and there is an interdependency between parameters of the Dirichlet and the multinomial distributions, permitting identification of differences not easily found with the frequentist methods. Moreover, DMM quantifies and propagates the uncertainty associated with the OTU abundances in the parameter estimates, which make multiple comparison corrections unnecessary (Fordyce et al., 2011; Holmes et al., 2012; Harrison et al., 2020).

#### Method for analyzing combinations of bacteria

The algorithm we chose to adapt, Latent Dirichlet Allocation (LDA), is used frequently in computational linguistics, to find patterns in documents. It groups words into topics, easily discernible by human readers, and summarizes the documents in terms of these meaningful topics (Blei et al., 2003; Griffiths and Steyvers, 2004). Here, we used LDA to find patterns in bacterial abundances in an analogous way.

Below, we describe how to set up the abundance data for use in the algorithm, and then, at a high level, we describe how the algorithm works and the mathematical form of its results. Because of the challenges of understanding and interpreting LDA’s results, we also present a graph theoretic methodology for doing so. The details of the algorithm and our computations are described in the **Supplementary Methods**. LDA is the core of the methodology that will enable us to uncover relationships relating bacteria at different times to AD and to describe microscopic arrangements of possibly pathogenic sets of bacteria and their large-scale macroscopic distribution in the brain.

##### Transforming abundance data for LDA

Like the original implementation of LDA which used words as the data, we needed a discrete form of the data to work with. The microbial objects that combined microbial name and abundance bin, described above, sufficed, but with a few changes.

For many of the lower-occurring and lower-abundance objects, we surmised that we needed even less abundance resolution, so we reduced the resolution to two bins instead of 14. A number of very low-occurring objects were also merged together under a general name instead of the individual bacterial names. Some taxa were grouped to species and some to genus levels. We did not group more by going further up the phylogenetic tree because we did not wish to lose behavioral information. We did not know *a priori* that this would work but had prior experience analyzing the gut microbiomes of ~7,000 subjects. This merging was performed with well-defined rules, primarily based on abundance resolution or occurrence, to maintain objectivity. We review the heuristic optimization procedures we used in the **Supplementary Methods**.

These changes to the data binning improved the similarity between pairs of samples that was limited by the sparsity of microbial measurements and large width of the microbial abundance distributions. For example, if two samples both had a high but different abundance of a particular species, they now both contained the same object, “bacteria-name-hi”. Overall, this scheme reduced the sparsity and allowed the algorithm to perform better.

##### Summary of Latent Dirichlet Allocation (LDA)

In order to gain a sense for how the LDA algorithm works and how its results are expressed, we first describe its use for documents and then its adaptation to microbiome data.

The LDA (Blei et al., 2003; Griffiths and Steyvers, 2004) algorithm works by classifying words, i.e., assigning classes (topics in the literature) to each word. It does not directly classify a document. Documents are “classified” by statistically summarizing the fraction of their words assigned to each class. Similarly, unique words are “classified” by summarizing the fraction of times they are assigned to each class over the entire set of documents. This word classification summary is how LDA reveals that a particular word may have more than one meaning. Classes (topics) are summarized by the fraction of times each word in the set of documents is assigned to a class. Class summaries are also how LDA reveals how a set of words has a common meaning forming a topic. LDA works by locating word co-occurrence within a document. Another way of saying this is that LDA finds the context of words.

It will become clear shortly why it is important to stress that document classifications, word classifications, and class summaries are not words. They are statistical summaries of word classifications.

Our implementation of LDA’s words, microbial objects, carry information about both microbial behavior (i.e., its identified name) and an abundance which is the result of underlying microscopic ecosystems summed over the sample. We thought that if we used the LDA algorithm to classify these objects, biological meaning could be revealed by a relationship between class and the occurrence of AD.

**Figure 1:**
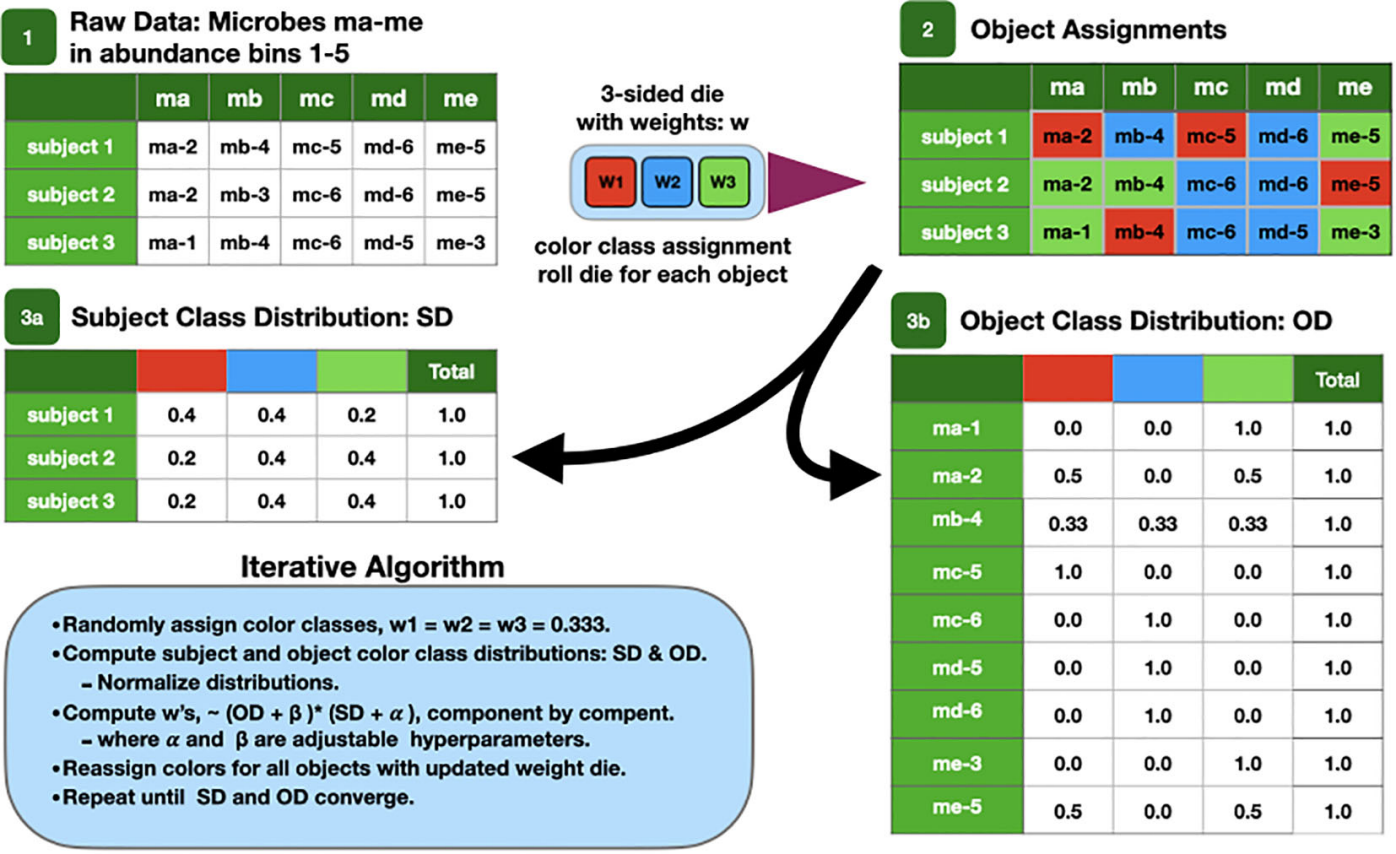
Cartoon of the LDA algorithm.

These microbial objects are assigned one of a preset number of classes using the LDA algorithm. A cartoon version of the algorithm is described at a high level in **Figure 1** and described in detail in the **Supplementary Methods**.

The LDA results data are tabulated in two tables. The sample distribution table has a row for each sample, and its columns are labeled by class. Its entries are the number of times the sample’s objects were classified with a particular class. The rows can be normalized to show the fraction of objects classified in a class. The object distribution table has a row for each unique object with columns labeled by class. One microbe may have multiple rows corresponding to different abundance bins. The entries are the number of times objects were classified with a particular class for the entire data set. The object class distribution is computed by normalizing across the rows to show the fraction of times each object is assigned to a class. In either representation, the rows should be thought of as vector-valued classifications. In other words, samples and objects have multi-valued classifications.

If the object table were normalized by column instead, the composition of each class is revealed by microbial object. In other words, the normalized column is the computed microbiome. Its elements are the fractional number of times each object is classified into a particular class and what we call the rigorous microbiome.

The existence of a class structure within a data set provides an opportunity to uncover patterns that can be missed by methods that ignore class. In fact, ignoring class structure implicitly averages out the very evidence that is sought.

In order to make analysis and discussion easier, we label the components of the classifications with colors. From this, we define the color of a sample or object to be the color of its largest component. For example, a red sample’s largest component is the red component. We also use the concept of color to approximate or describe microbiomes. In this example, it can be thought of as the set of microbial objects that occur in samples of a given color.

Because the number of classes is much less than the number of objects or the number of bacterial species, LDA results in a dimensionality reduction of the data. The number of classes is determined by an optimization process discussed in the **Supplementary Methods**. This should not be confused with the dimensionality reduction achieved by PCA. Typically, PCA evidences sample clustering by plotting two different linear combinations of abundances for each sample, the principal components, in two dimensions. These clusters are not the same as LDA’s. Other dimensionality reduction schemes, e.g., t-SNE and UMAP (van der Maaten and Hinton, 2008; McInnes et al., 2018), work in a different way, and it is not clear that they can easily allow objects (or measurements) to have multiple meanings although the common objects within the samples of a sample cluster in low dimensions could represent common meaning the way a class does in LDA. More information is provided in the **Supplementary Methods**, but a detailed discussion is beyond the scope of this work.

We emphasize that LDA’s results are not abundances themselves or even linear combinations of abundances. Rather, they are statistical summaries of microbial object classifications, which are measures of the co-occurrence or context of multiple objects that are described by their class composition.

Our adaptation of LDA to small microbiome data sets also involved several other procedures, which are discussed in detail in the **Supplementary Methods**. From here on, we will refer to our implementation as modified LDA or MLDA.

##### Higher-order statistics

The next step in the methodology is to construct statistics of the MLDA results to infer information about microbial spatial distributions at the microscopic cellular and macroscopic brain levels, how they change over time, and what their relationship is with AD.

Since we know the sample spatial positions, we can look at how class varies with position. This is not the same as looking at how a particular microbe’s abundance varies with position because the same microbial object may have significant components in more than one class and their spatial positions could be different. For example, we determine in which lobes possible pathogenic classes predominate. Other statistics derived from the occurrence of class by subject can be determined whether the spatial distribution of classes is random or regionally clustered, suggesting where it is located with respect to the brain’s physiological structures and the paths taken to the brain.

Second, we can look at how abundance occurrence is distributed within class for particular bacteria, especially the principals. The width of this distribution is associated with the microscopic ecosystem spatial arrangements. Narrower distribution suggests more microscopic homogeneity within the sample, and wider distributions are associated with more heterogeneity, both of which suggest how far apart different principal bacteria are from one another.

Similarities among the classes of samples and the assumption that health precedes disease suggests how to order them in time. Finally, relationships between sample classes with subject suggests possible pathogenic microbiomes. In other words, the higher-order statistics permits identification of the temporal-spatial aspects of relationships that could be evidence for a bacterial component in the etiology of Alzheimer’s disease.

##### Graphs

The patterns found by MLDA are sometimes difficult to understand so we developed graphical visualization techniques to assist us. Additional details can be found in the **Supplementary Methods** (Also see Results) section).

###### Type I graphs

This type of graph, where the nodes are samples, was designed to display classification results, sample similarity, metadata values, and metadata statistics. A glance enables you to get a sense of the quality of the classification and see the presence of statistical fluctuations in the classification, which are the nodes outside the clusters. The graph helps to reveal gross features of the classification, which may relate to the emergent features of the microscopic ecosystem biology. The graphs were drawn using Wolfram Mathematica (Wolfram Research Inc, 2010; Wolfram Research Inc, 2021). It is possible to define a Type II graph that uses objects as nodes.

###### Nodes

Each node represents a sample.

###### Color

Each node is colored with the sample’s color. The MLDA computations result in each sample being described by C components, where C is the preset number of classes used by the MLDA algorithm. The color of a node should not be confused with an exclusive classification of the node. While each node is, in fact, described by a mixture of C components, the ubiquitous existence of color clusters in the graph suggests that the exclusive classification suggested by the colors is an approximation that is justified.

Node size. Nodes are enlarged (other graphs below) if a sample contained one or more specific microbial objects of interest. This visualization is used frequently to explore the class location of objects of the same microbe but differing abundance bin.

###### Node shape

The shape of the node displays the subject metadata value—diamonds for AD, circles for controls. Typically, we may note the diamond fraction statistic next to a color cluster. This AD statistic is the number of diamonds in the cluster divided by the total number of nodes in the color cluster. In our data, we have roughly 50% of the samples from AD subjects and 50% from controls. Thus, if the class means something for AD, the diamond statistic should be way over 50% if there is a correlation with AD or way less than 50% if the class is anti-correlated with AD. The fact that this is not the case is something we address.

###### Edge

Edges were defined by node pair similarity. In general, many types of similarities can be used but we used a coarse measure, the dot product. In this case, the similarity is the product of each pair of components, summed together. This is further discussed in the **Supplementary Methods**.

###### Node position

The features above define the topology of the graph—how the nodes were connected (Wolfram Research Inc, 2010). An embedding algorithm is used to position the nodes in 2D or 3D space. The algorithm finds the equilibrium position of the nodes when the nodes and edges are given physical properties that both repel and attract the nodes. The repulsion is computed by assuming that each node possesses the same electrical charge, and the attraction derives from representing each edge as a spring. This algorithm is known as spring-electrical embedding (Wolfram Research Inc, 2021), and the resultant graphs are called force-directed graphs. Because nodes that are the most similar are connected by springs, samples that are the most similar are pulled together in clusters. Nodes that are similar are found near one another, and nodes that are not similar are located far away from each other.

## Results

### Individual bacteria

After data filtering (low-yield samples and contaminant removal), 548 OTUs and 108 samples remained. Infrequent OTUs (present in less than 20% of the samples) and low abundance OTUs (relative abundance ≤0.005%) were grouped into a composite feature named “OTU others”. After this step, the dataset contained 108 samples and 247 OTUs (including “OTU others”). OTUs were assigned to 229 species; although most of the species correspond to a single OTU, 14 species were assigned up to 3 OTUs.

At the phylum level, the major components (i.e., those with higher average relative abundance) were Proteobacteria (control = 47.35%, AD = 46.35%), Actinobacteria (control = 35.65%, AD = 30.62%), Firmicutes (control = 10.80%, AD = 15.17%), and Bacteroidetes (control = 5.44%, AD = 6.11%). Three OTUs showed a broad prevalence across samples and were present in more than 50% of samples. They were assigned to the species *Cutibacterium acnes* (control = 82.69%, AD = 91.07%), *Acinetobacter junii* (control = 67.31%, AD = 55.36%), and *Staphylococcus epidermidis* (control = 55.77%, AD = 60.71%). There were 23 OTUs present in more than 10% of the samples, and 93 OTUs were observed in only one sample.

**Figure 2:**
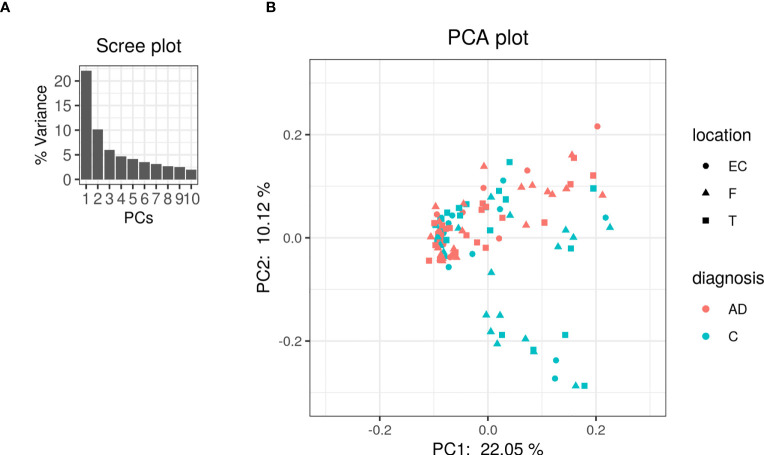
PCA was performed on clr-transformed composition. **(A)** Scree plot for the PCA ordination. Each bar corresponds to one axis of the PCA; the height is proportional to the amount of variance explained by that axis. **(B)** PCA ordination plot. Each colored point represents a sample. Points are colored by diagnosis and shaped by biopsy location (EC: entorhinal cortex, F: frontal lobe and T: temporal lobe).

The PCA on the clr-transformed OTU counts did not reveal any notable clusters of samples related to the disease status or biopsy sites (**Figure 2**), except for 14 control samples from 6 subjects that clustered together at the bottom of the PCA space. Only 32% of the variance was explained by the two first components.

**Figure 3:**
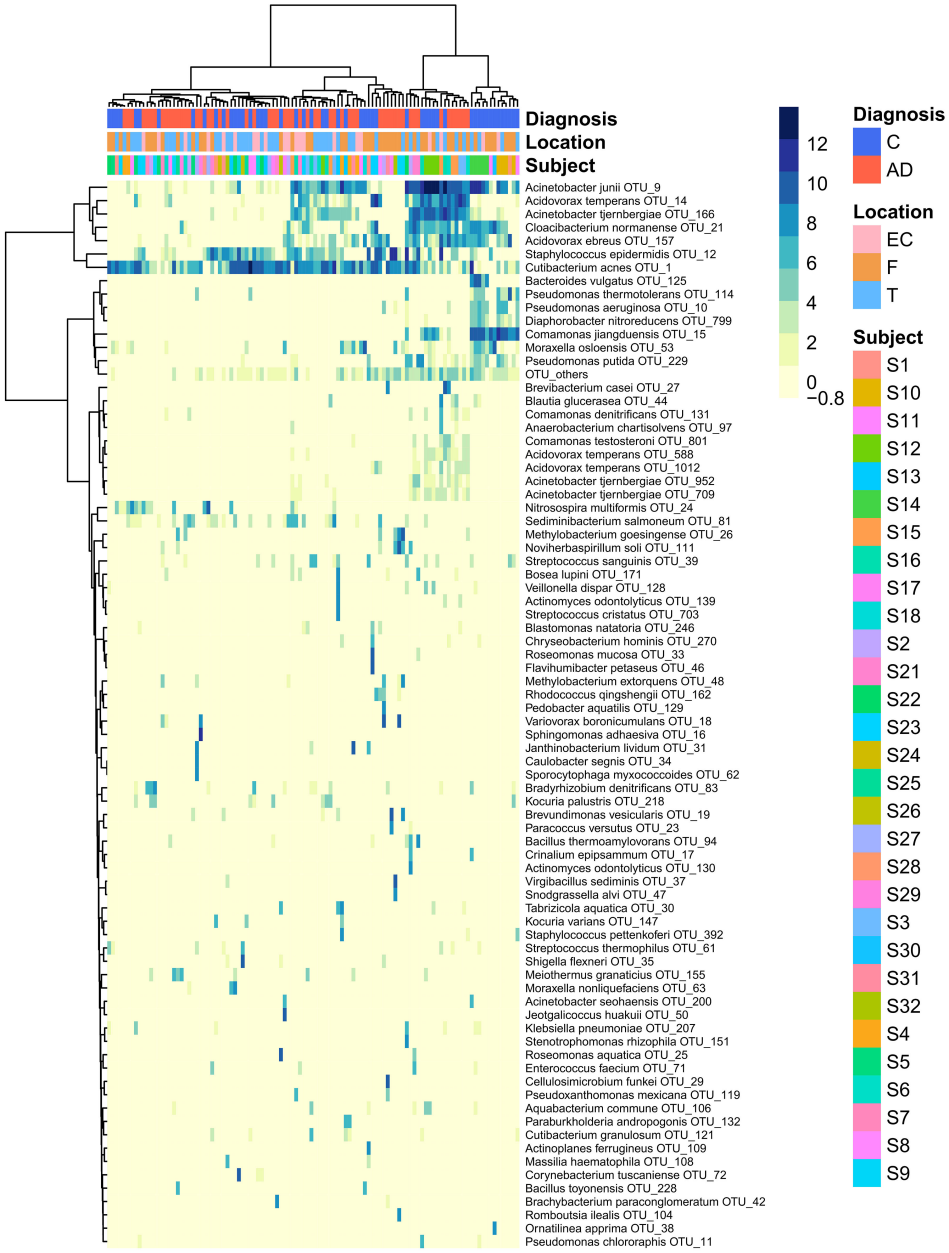
Heatmap that represents the clr-transformed OTU counts within each sample of the 80 most variable OTUs (higher relative abundance corresponds to darker colors). Unsupervised grouping of samples with similar OTU composition (columns) and OTUs with similar abundance across samples (vertical) into clusters was achieved by hierarchical clustering using the Euclidean distance between clr-transformed compositions. The sample’s subjects, biopsy brain locations and diagnosis are indicated by the vertical colored strips. AD, Alzheimer’s disease; C, controls; EC, entorhinal cortex; F, frontal lobe; T, temporal lobe.

The heatmap of the top 80 most variable OTUs, where the OTUs and the samples were grouped by hierarchical clustering, shows that most of the samples were dominated by the same OTUs but did not evidence any pattern related to AD or control groups (**Figure 3**).

#### Difference in relative abundance between AD and controls

**Figure 4:**
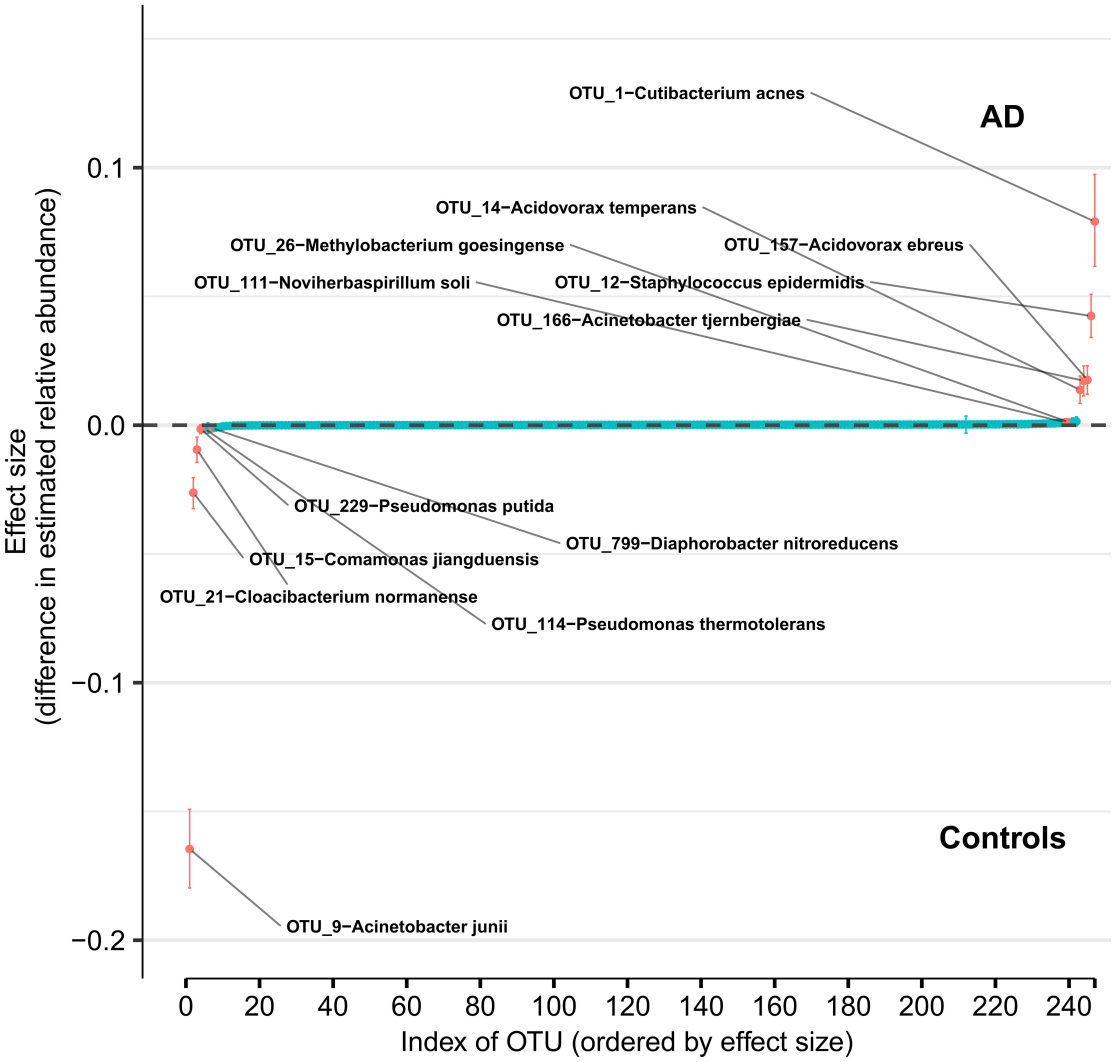
Differences in relative abundance between the Alzheimer’s disease (AD) group and the age-matched control group. The relative abundances were estimated for each OTU from each group through Dirichlet-multinomial modelling. The vertical axis shows the estimated differences in the relative abundance of each OTU between the AD and control groups. Points are the means of the posterior probability distribution of differences (PPD) and the whiskers show the 95% equal tail probability intervals of PPD (see Materials and Methods and Supplement).

Using DMM and assuming sample non-independence due to multiple samples coming from a single subject in the model, we found 12 OTUs that shift in relative abundance between AD and control groups (**Figure 4**). Six OTUs are more abundant in the control group: *Acinetobacter junii*, *Comamonas jiangduensis*, *Cloacibacterium normanense*, *Pseudomonas putida*, *Pseudomonas thermotolerans*, and *Diaphorobacter nitroreducens. C. jiangduensis*, *C. normanense*, *D. nitroreducens*, and *P. putida* have low species-level confidence values (**Table S2**). The most important shift is in *A. junii.* Seven OTUs are more abundant in the AD group (*Cutibacterium acnes*, *Staphylococcus epidermidis*, *Acidovorax ebreus*, *Acinetobacter tjernbergiae*, *Acidovorax temperans*, *Noviherbaspirillum soli*, and *Methylobacterium goesingense*). *A. ebreus*, *A. tjernbergiae*, and *N. soli* show very low species-confidence values (0.2112, 0.1169, and 0.0076, respectively). The most important change was in *C. acnes*. When the non-independence of the samples is ignored, the same results are obtained for *A. junii*, *C. jiangduensis*, *C. normanense*, *A. temperans*, *A. tjernbergiae*, *A. ebreus*, *S. epidermidis*, and *C. acnes*, whereas no shift in relative abundance has been detected for *P. putida*, *P. thermotolerans*, *N. soli*, and *M. goesingense* (**Figure S1**).

### Combinations of bacteria

#### Introduction

Overall, we organized the results according to four themes: (1) the color classes, their microbiomes, and their principal bacteria as revealed by the MLDA classification and graph methods; (2) microbe object abundance statistics that were used to infer the spatial distributions of underlying cellular-scale ecosystems and the macroscopic distribution of ecosystem mixtures by class; (3) the relationships between the classes that will be used to determine the temporal order of the classes assuming each class represents different stages of underlying ecosystem evolution; and (4) the occurrence of the classes within each subject that suggests the pathogenicity of the ecosystems within each class. In this section, we focus on the mathematical results without detailed discussion of the ecosystem biology, which will come in the discussion section.

#### Theme 1—color classes and their microbiomes

##### Microbiome description

**Figure 5:**
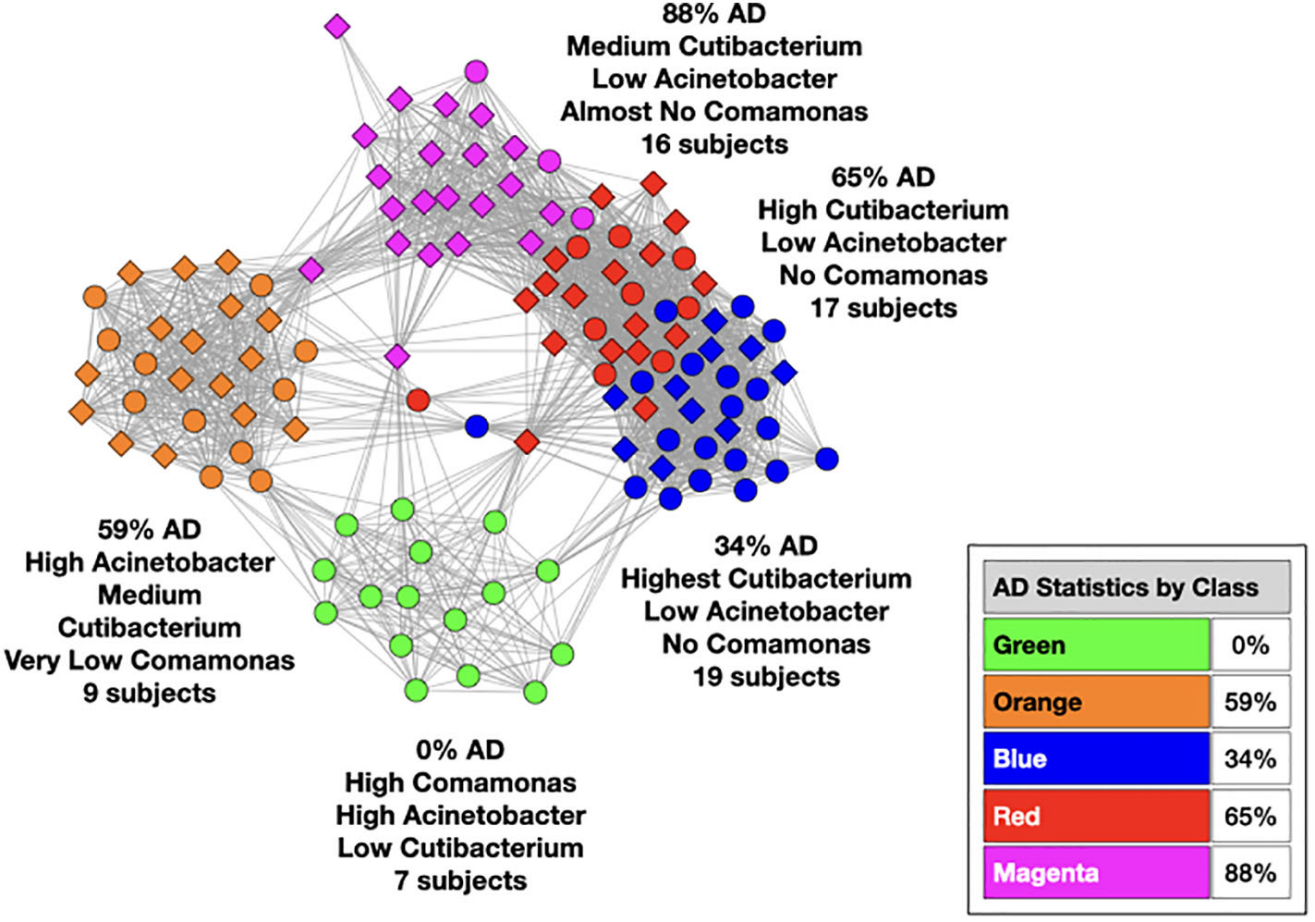
Type I graph. Results from summation of five runs. Nodes are samples. Colors are maximum classes. Principal bacterial genera and abundance levels indicated for each color. The inset contains the percentage of samples that come from AD subjects by class, called AD statistics.

We used MLDA to compute five distinct color classes of samples. The results of our computations are summarized in a graph shown in **Figure 5**, which is the sum of five different stochastic runs. We chose five classes as the optimum for our computation with the methods described previously.

The set of microbial objects resulting from a statistical summary of microbial objects from the samples in each color cluster will be called the class microbiome in the following paragraphs, which approximates the rigorous microbiome. The observed microbial objects derive from the summation of one or more ecosystems at the cellular scale during the physical sampling process. We will show how to characterize these ecosystems and how they determine the sample measurements in the discussion section.

In the graph (see **Figure 5**) of these five homogeneously colored clusters, two are very distinct, the green and orange, whereas the other three are merged together: blue, red, and magenta. This means the green and orange microbiomes are both different from one another and different from the blue, red, and magenta supercluster microbiomes. On the other hand, the latter are more related to one another since they are closer to one another. Each cluster represents a different underlying microbiome with a different set of microbial objects statistics. The green and orange sets must therefore have very different objects whereas the blue, red, and magenta have objects in common, specifically blue with red and red with magenta although not blue with magenta. As we have pointed out before, common objects between classes could be suggestive of multiple biological meanings of an object. This is one of the peculiarities and benefits of MLDA, that identical measurements mean different things in different contexts.

The statistical results shown in **Table S3**, **Table S4**, and **Table S5** show these microbiome summaries for each of the color classes for objects with abundance bin ≥10 (0.3% abundance) and counts ≥5 in **Table S3** and ≥2 in **Table S4** and **Table S5**. Different tables show different abundance combinations for particular microbes to show the importance of various microbes. **Table S7** shows the same information in a different form. Each object is shown with their approximate microbiome computed from the occurrence count of the object in the sample color class.

For each color class, we also show the fraction of samples that come from AD subjects. Diamond-shaped nodes are from AD subjects and circles from the controls. It does not necessarily follow that this number is an estimate of the pathogenicity of the underlying microbiome, which will be explained in Theme 4 below.

##### A few graph anomalies

Note that several nodes fall outside the clusters. This occurs because MLDA uses a stochastic algorithm which creates statistical fluctuations in class assignments. When the two largest class components are close, sometimes one ends up a little bigger and sometimes the other. The sample will be pulled from the cluster if one of these ends up being too large.

##### Principal microbes and microbial objects

**Figure 6:**
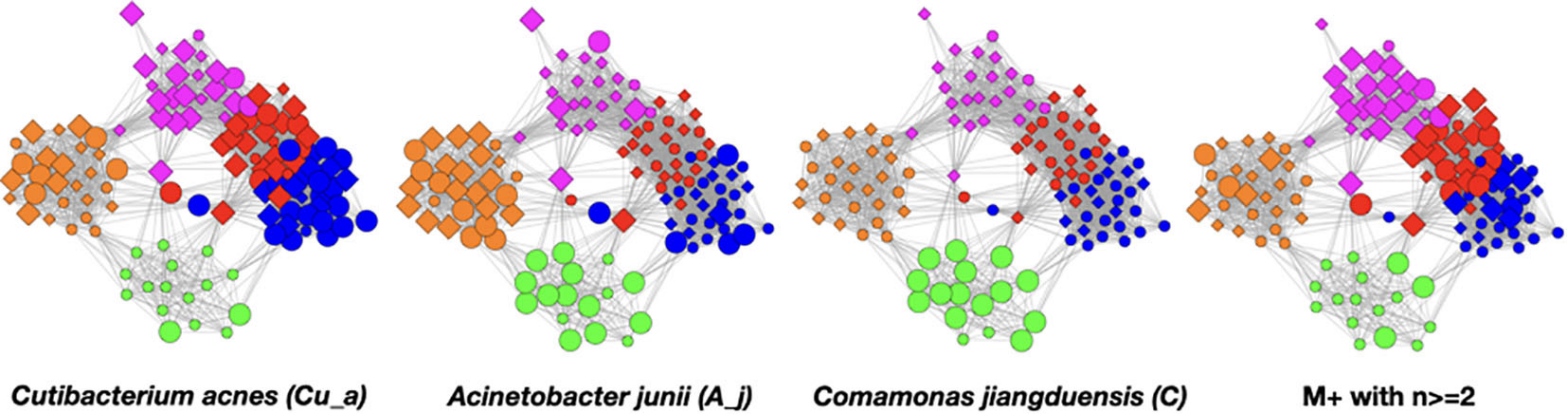
Graphs showing samples with abundances levels 12–14 with enlarged nodes for three main microbes.

In the **Figure 6** graphs, we show the samples in which the most abundant species are found, combining their objects in abundance ranges 12–14. The samples that contain these objects are shown by enlarging the sample/nodes containing these microbial objects. *C. acnes* is most prevalent in the blue–red–magenta supercluster with some in the orange. The blue is dominated by the 14 abundance range, the red has a combination of 14 and 13 abundance ranges, and the magenta has a combination of 13, 12, and 11 abundance ranges. *Acinetobacter junii* is mainly found in the green and orange classes and *Comamonas jiangduensis* is found almost exclusively in the green class. These underlying details can be found in **Table S3**, **Table S4**, **Table S5**, **Table S7**, and **Table 4**.

##### Low-count microbial objects

**Figure 7:**
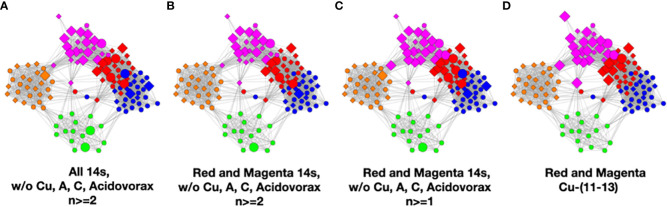
Several definitions of M+ compared to *C. acnes* (11–13). **(A)** Objects with level 14 that occur two or more times in any class, **(B)** objects of level 14 that occur two or more times in magenta or red, **(C)** objects of level 14 that occur two or more times and their corresponding objects of level 13 in magenta or red, and **(D)** *C. acnes* (11-13).

To understand the low-count microbial objects, it is best to look at high-abundance and low-abundance objects separately. Because we are spreading low-count objects over five classes, it is difficult to find statistically significant patterns among them. In fact, we were unable to find any statistically sound patterns among the low-count and low-abundance objects. We did, however, find a fundamentally important pattern for high-abundance low-count objects that occurs mainly in the magenta and red classes, although signs of it can be traced to the other classes too; see **Table 4**. Specifically, we noticed that samples with *C. acnes* with abundances 11–13 in the red and magenta classes correlated with a set of low-count bacteria with abundance level 14. In most cases, there was only one that occurred per sample. In **Figure 7**, we show two different ways of defining this set.

In **Figure 7**, we illustrate this point in a few different ways. Graphs a–c show low-count high-abundance objects in the sample with various occurrence rates. The right shows *C. acnes-*(11-13) objects to demonstrate that they co-occur in many samples with the low-count high-abundance objects. From here on, we will refer to the abundance 14 objects of (c), occurring in the red and magenta classes, as the M+ set because the most prevalent member of the set is *Methylobacterium.* In fact, of the 28 samples where these *C. acnes* objects occur, 22 contain M+ objects. The nine M+ objects that occur two or more times are *Bacillus*, *Bradyrhizobium*, *Caulobacter*, *Delftia*, *Kocuria*, *Methylobacterium*, *Nitrosospira*, *Sediminibacterium*, and *Variovorax*. Their objects can also be found in **Table S4**.

##### Robustness of color clusters

Three of the color classes have a heterogeneous mix of AD and control samples (orange, blue, and red). The other two, green and magenta, are nearly homogeneous in the disease state, comprising almost entirely samples from either AD or control subjects. We initially thought that we should observe clusters whose disease state was nearly homogeneous, meaning that either a microbiome was pathogenic or not. The reason for the heterogeneity will become clear with the results of Theme 4 below.

We thought that these statistics suggested that there may be within-class differences in the microbiomes of the AD and control samples that could split the orange, blue, and red classes into homogeneous color clusters with the right input and object merging parameters. Indeed, there were within-class differences between the cohorts, but when we tried to split the clusters by using a larger number of input classes with these adjustments, the clusters would not split. The object differences between samples were just not large enough to support entirely new color classes.

##### Correspondence with results of method I

We will look at three aspects of these results in light of the MLDA results. First, we will compare the PCA method’s capability of identifying microbiome clusters; second, we will look at the bacteria that were identified as being correlated or anti-correlated with AD; and last, we will look at a contamination issue.

**Figure 8:**
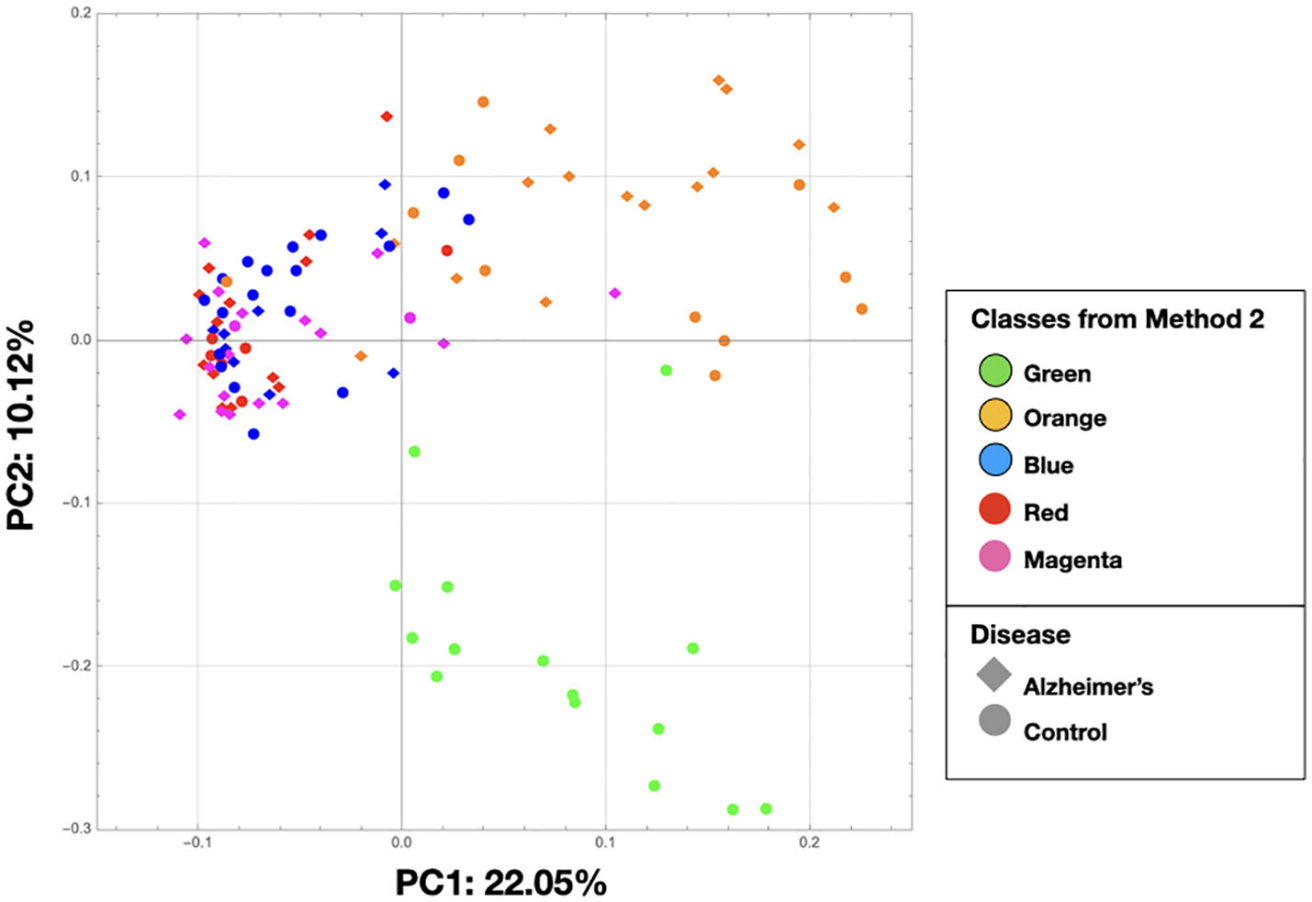
This is the same as **Figure 2**, but the nodes are colored by the class colors.

In **Figure 8**, we show the same chart as **Figure 2** except that the nodes are colored by the class colors of the MLDA results. The green and orange samples are distinctly clustered whereas the blue, red, and magenta are mixed together. While the orange are clustered, there is not an obvious way to distinguish the colors. The MLDA plus graph does show the blue, red, and magenta samples in a super cluster; however, these color clusters are distinct and defined by the maximum class component whereas they are interspersed in the PCA results. A complete reconciliation of the PCA and MLDA results is beyond the scope of this paper.

For the following, see **Table S3**, **Table S4**, and **Table S5**. The DMM method reveals *Cutibacterium acnes* as associated with AD. DMM has no class structure, so the method essentially averages over it. As we discuss both in this section and the next, this association is likely due to the prevalence of *C. acnes* in abundance levels 11–13 in the red and magenta clusters being high enough not to be washed out by its presence elsewhere where it is frequently found in the controls.

The method also found *Comamonas jiangduensis* to be correlated with the controls, which is also found with the MLDA method. Regarding *Acinetobacter*, one species *A. junii* was found to be anti-correlated with AD and another *A. tjernbergiae* was correlated with AD. *A. junii* objects are found in both the green and orange classes, with green not being associated with AD and orange somewhat associated with AD. *A. tjernbergiae* objects are mainly found in the orange class. Overall, since DMM is essentially averaging over the classes, these results appear to be consistent with the MLDA results.

Last, but importantly, the *Sediminibacterium* and *Methylobacterium* species identified by DMM as associated with AD is consistent with the MLDA findings. These two are among the M+ bacterial set that is found in the red and magenta classes. *Methylobacterium* is mainly found in the samples of the magenta class, which is most associated with AD. Other findings of DMM can be reconciled with MLDA by referring to **Table S3**, **Table S4**, and **Table S5**. Overall, correlation of the results with AD is more complicated than this discussion and requires an understanding of the subject level results.

The first method found that *Staphylococcus epidermidis* was associated with AD. The main OTUs in this species were, however, removed in the background removal process for the second method because all of the OTUs present in the negative controls were removed even if there was only a small amount as was the case for this OTU. A post-MLDA analysis found that *S. epidermidis* was present in 45 samples ≥ abundance level 11 and in 39 samples ≥ abundance level 12. In this analysis, we were able to estimate the class distribution of *S. epidermis* objects and found that their class distributions were fairly flat, which is consistent with a contaminant. Furthermore, its objects with abundances ≥12 come from AD samples 59% of the time partially accounting for the DMM result.

In summary, the results of the DMM analysis are what happens when class is not considered in an analysis. As it is a confounding variable, ignoring it can sometimes skew results, although not always.

#### Theme 2—microscopic structure and macroscopic spatial distribution

There are two sets of results that provide information about the macroscopic spatial distribution of ecosystem mixtures and microscopic spatial distribution of individual ecosystems within the samples. We describe the results here and review their detailed relationship to ecosystems in the discussion section.

##### Graph clustering and macroscopic structure

A fundamental result of the graph visualization of the MLDA results is the appearance of homogeneously colored clusters. This occurs because the value of the maximum MLDA component (color) of the node classification vector is ⪆0.4, showing that a particular class dominates in each sample. Furthermore, each color cluster contains samples from many subjects suggesting similarities in microbiome across subjects. Even though each subject has been undersampled (two to five samples/brain), the class structure suggests picturing the physical sampling process as coming from two virtual brains, one with AD and one without AD, each with about 60 samples/brain. This grouping can inform the large-scale macroscopic distribution of the individual microbiome classes.

##### Abundance distributions of principal bacteria and microscopic structure

By examining the actual underlying objects in each color class, we can learn even more. In **Table 4**, we present the abundance statistics of each of the principal objects in each class, specifically *C. acnes*, *A. junii*, *C. jianduensis*, and M+. See the **Supplementary Methods** for a detailed description of this table. The principal *C. acnes*, *A. junii*, *C. jianduensis*, and M+ object abundance averages and distributions within a class provide information about the microscopic ecosystem structure, specifically their density and their spatial homogeneity on sample scales. In other words, these statistics provide information regarding the structure within the sample from which we can infer a microscopic structure.

**Table 4:** Principal bacteria abundance distributions. Note that the M+ rows are different because they show the occurrence of any of 21 different genera in the M+ set.

	Microbe/Abundance Bin	7	8	9	10	11	12	13	14	tot/tot-class	Abundance	Width	Avg	SD	Sum
Green	**C. acnes**	1	1	0	1	3	0	1	1	8/16	low	wide	10.6	2.3	8.0
	**A. junii**	0	0	0	1	0	2	5	5	13/16	med	narrow	13.0	1.2	13.0
	**C. jianduensis**	0	0	0	0	1	2	5	7	15/16	med	narrow	13.2	0.94	15
	**M+ n≥2**	0	4	1	4	3	1	1	1	8/16	low	wide	10.2	1.9	15
	**M+**	2	7	6	6	6	2	1	1	15/16	low	wide	9.7	1.7	31
Orange	**C. acnes**	2	1	3	3	2	3	7	2	23/27	med	med	11.1	2.2	23
	**A. junii**	0	0	0	0	1	0	12	11	24/27	high	narrow	13.4	0.71	24
	**C. jianduensis**	2	0	1	2	3	0	0	0	8/27	low	wide	9.5	1.7	8.0
	**M+ n≥2**	1	4	4	5	5	5	1	0	15/27	low	wide	10.1	1.6	25.0
	**M+**	1	7	8	8	7	6	2	1	20/27	low	wide	10.1	1.7	40.0
Blue	**C. acnes**	0	0	0	0	0	0	0	27	27/29	high	narrow	14.0	0	27
	**A. junii**	0	0	3	5	2	3	3	0	16/29	low	wide	10.9	1.5	16
	**C. jianduensis**	0	0	0	0	0	0	0	0	0/29	none	none	0.0	N/A	0
	**M+ n≥2**	0	5	3	11	5	5	1	1	18/29	low	med	10.3	1.5	31
	**M+**	0	5	6	16	10	9	4	3	24/29	low	med	10.7	1.6	53
Red	**C. acnes**	0	0	0	0	1	2	6	14	23/26	high	narrow	13.4	0.84	23
	**A. junii**	0	0	0	1	2	0	1	0	4/26	low	wide	11.3	1.3	4
	**C. jianduensis**	0	0	0	0	0	0	0	0	0/26	none	none	0.0	N/A	0
	**M+ n≥2**	0	1	1	4	9	10	9	7	25/26	med	med	12.0	1.5	41
	**M+**	0	1	1	4	9	12	11	10	25/26	med	med	12.1	1.5	48
Magenta	**C. acnes**	0	0	0	1	3	7	9	0	20/24	med	narrow	12.2	0.89	20
	**A. junii**	0	0	0	0	0	3	1	1	5/24	med	narrow	12.6	0.89	5
	**C. jianduensis**	0	0	0	0	0	0	0	0	0/24	none	none	0.0	N/A	0
	**M+ n≥2**	0	0	2	5	2	5	6	12	21/24	med	med	12.4	1.7	32
	**M+**	1	1	2	7	6	9	8	19	24/24	med	med	12.2	1.8	53

We show the occurrence of each object in each class and then characterize the width of the abundance distribution over bins. The average abundance is related to the density of underlying ecosystems, and the width of the abundance is related to the spatial homogeneity of ecosystems within a sample. More details of this interpretation are provided in the discussion. Generally, when the abundance average is ~14, we call it high; between 13 and 14, we call medium; and everything else is called low. Also, when the distribution is concentrated in one or two bins, we call it narrow, mainly two or three bins with some peaking, medium, and greater than or equal to three bins, we call wide. We will explain in the discussion how to predict the spatial distribution of underlying ecosystem mixtures from these results, which is not obvious because it depends on the ecosystem model. The sample data are presented in **Table S6**.

In presenting the results in this manner, we also need to call attention to an important equivalence principle that we use to understand these results. Furthermore, we emphasize that we are assuming that the microbial objects used in the computations result from summing over physically sampled mixtures of ecosystems, but we do not know much about the ecosystems yet. Therefore, we are assuming that each class microbiome results from a different mixture of ecosystems. The principle is as follows.

**The sum of the virtual sampling of ecosystem mixtures equals the physical sampling of the sum of the ecosystem mixtures**

In other words, we can treat the results of **Table 4** as what we would obtain had we been able to individually sample a single ecosystem class mixture. We can then use these results to derive something about the nature of the individual class ecosystem mixtures. This is done in the discussion section.

#### Theme 3—temporal order of the classes

Temporally ordering the classes requires finding relationships between pairs of classes and then determining their temporal order. Below, we describe statistical methods that relate class pairs where it can be argued that the method is finding classes where the underlying ecosystems could evolve from one class to the other. Using these pairs, together with arguments about temporal order, we construct a time-ordered network of the classes.

Note: There are many interesting correlations and anomalies found in the data, most of which can be accounted for by our methods. Some involve spurious time correlations, and others involve competitive interactions. We have not included these here but mention them to emphasize the usefulness of these methods to explain far more than we present.

##### Microbial abundance dynamics

A strong statistical relationship between pairs of color clusters, which might indicate a temporal relationship, should involve samples that contain principal microbial objects whose abundances are the same or differ by one. These are situations where it is likely that one microbe is just beginning to outcompete others or the reverse.

**Figure 9:**
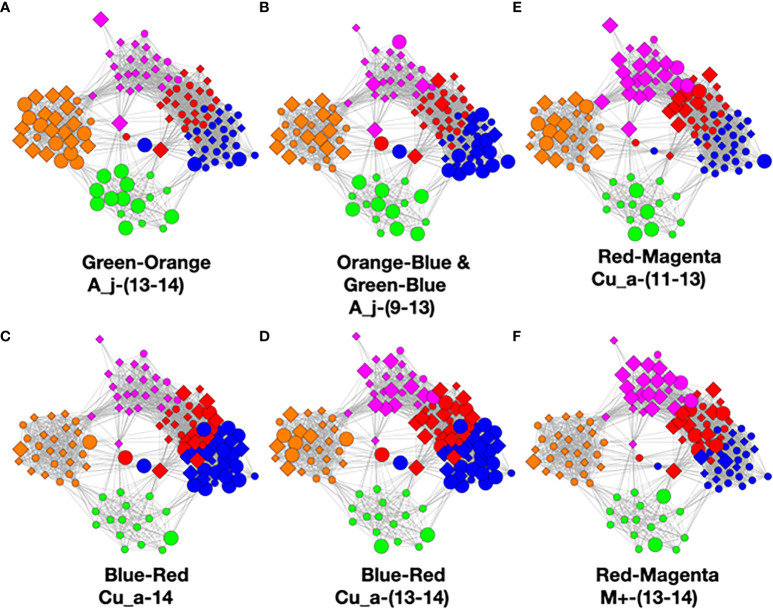
Color pair relationships. **(A)** Green-Orange: A_j -(13-14), **(B)** Orange-Blue and Green-Blue: A_j,-(9-13), **(C)** Blue- Red: Cu_a-14, **(D)** Blue-Red: Cu_a-(13-14), **(E)** Red-Magenta: Cu_a-(11-13), **(F)** Red- Magenta: M+ -(13-14).

To visualize this, we constructed graphs where the samples of class pairs that meet these criteria are enlarged. In **Figure 9**, we show results for the highly occurring species of the *Cutibacterium*, *Acinetobacter*, and *Comamonas* genera as well as the low-count high-abundance objects referred to as M+. Refer to **Table 4** for more details. For less frequently occurring objects, the prevalences are too low to be useful.

This analysis found that the green–orange, orange–blue, green–blue, blue–red, and red–magenta pairs showed the strongest relationships utilizing the above evaluation standards. Refer to the **Supplementary Methods** for detailed discussion of the comparisons.

##### Time ordering of classes

Now that we have established relationships between pairs of classes, it is straightforward to order them in time. To do this, we need a beginning and an end which is provided by AD statistics and the reasonable assumption that health precedes disease. Earlier, we cautioned about the use of these statistics because it does not necessarily follow that the bacteria in all of the samples of an AD subject are necessarily responsible for the disease. This is most likely not the case, however, for the green and magenta classes.

When the class AD statistic is either close to 0% or close to 100%, we are looking at situations where the population either never came from a diseased subject (green) or almost always came from a diseased subject (magenta). In these cases, the former would most likely not be pathogenic, or the subject would have AD. As the latter is almost always associated with disease, it is a reasonable hypothesis that it is pathogenic. Given that all the other sample colors are associated with both AD and controls, unless the physical sampling of the subject brains somehow missed a part of the brain that contained other pathogenic ecosystems not in any of our data, the magenta class most likely contains pathogenic ecosystems.

**Figure 10:**
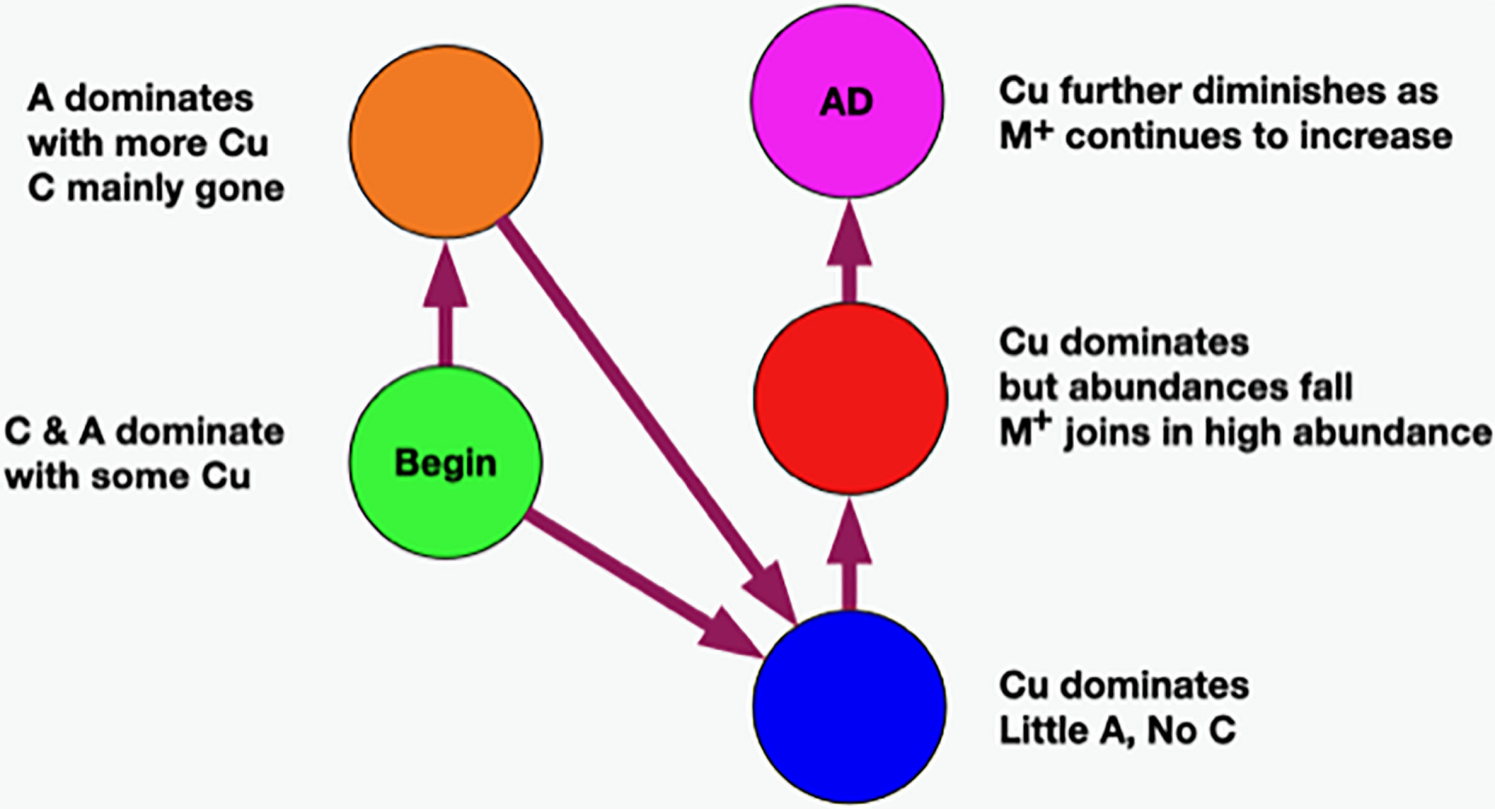
Temporal network of classes.

Refer to **Figure 10**. The only strong relationship to green is orange although there might be a minor link to blue. At the other end, the only relationship to magenta is red. The only one we are left with is blue to red which must go in between the former two. Now we have a time-ordered class network. Another way of saying this is that we have derived a temporal variable where class color is the variable.

##### Microbial dynamics and *C. acnes* anti-correlation

At this point, we observe that the network essentially reveals temporal dynamics for all of the principal bacteria, but most importantly, it has revealed the dynamics of *C. acnes*. It begins in the green class with a low average abundance and a broad distribution. It evolves in the orange to a medium average abundance that is still quite broad. In the blue class, it reaches a high narrow peak. In the red class, the abundance begins to fall and broaden. It falls further in the magenta class to a medium level with an even broader distribution.

In the green class, *A. junii* displays a medium average and narrow width. It stays roughly the same in the orange class. It then rapidly diminishes in blue and continues its decline to low abundance for red and magenta. The *C. jiangduensis* dynamic is more pronounced. It begins with a medium average abundance and width in green and then drops precipitously and broadens in orange. It is essentially not present in blue, red, or magenta.

On closer inspection, there seems to be another inter-object dynamic, an anti-correlation with *C. acnes* as seen in **Table 4**. In the green, *C. acnes* is either non-existent, as seen in half the samples or at very low abundances. Conversely, this is the class where the *Comamonas* and *Acinetobacter* species have the highest abundances and are the most prevalent. The orange class has the next lowest level of *C. acnes* where it is present in almost all of the samples but with only a small peak in abundance (at 13) observed with seven samples and displaying a very wide distribution ranging from 7 to 14. Curiously, there is virtually no *Comamonas*, but *Acinetobacter* persists at high levels.

In the blue class, the levels of *C. acnes* are the highest among all of the classes. There is virtually no *Comamonas*, and *A. junii* levels are low; the latter occurring in only about half the samples with a very wide abundance distribution from 9 to 13. In the red class, there is a high level of *C. acnes* but *A. junii* is further diminished being present in less than a quarter of the samples over a wide abundance distribution of 10–13. Even with somewhat lower levels of *C. acnes* in magenta compared with red, *A. junii* is still only present in fewer than a quarter of the samples over a distribution from 12 to 14.

Thus overall, it appears that when *C. acnes* is not present or is present only at low levels, we observe both *Comamonas* spp. and *Acinetobacter* spp. However, as the abundance of *C. acnes* increases, first the *Comamonas* spp. is lost and then the *Acinetobacter* spp. As the *C. acnes* abundance decreases from the red class to the magenta class, a new non-specific dynamic enters the mix. The high-abundance low-count microbes, M+, appear mainly in the upper red and magenta classes. They increase in the magenta class as the *C. acnes* abundance falls, resulting in an antiparallel dynamic. There is something very curious here that we will take up again in discussion of the ecosystems and the biology. The *C. acnes* distributions in the magenta class look similar to those in the orange class, but there are major differences between the classes otherwise. This is an example of objects with multiple meanings described in the Methods section that MLDA can identify. The orange has a lot of *Acinetobacter* spp. whereas magenta does not, and the magenta has a lot of M+ whereas the orange class does not. Clearly, the ecosystem evolution that goes along with *C. acnes*’ rise and fall is neither the same nor reversible. Time could provide the explanation. Some of the more prevalent M+ are present at low abundances and prevalence in the earliest stages, orange and green (see **Table 4**). Curiously, there is only one instance of *Methylobacterium* in orange and two in upper red. Given long enough, however, many seem to be able to take over, even if *C. acnes* is present, either by increasing from earlier lower levels, or coming onto the scene later, like *Methylobacterium*.

Overall, these findings will constrain the underlying ecosystem structure that is presented in the discussion section.

#### Theme 4—pathogenicity of classes

##### Disease state statistics

The disease state statistics of each class, which summarize the fraction of samples from AD subjects, provide the first clues about whether a particular microbiome is pathogenic or not, and we have used these clues to set the temporal order of classes. However, as we pointed out earlier, these numbers are not estimates of the actual pathogenicities of the classes for every class. These statistics summarize the fraction of samples from a class that come from a subject who has AD.

If it were assumed that class AD statistics were an estimate of class pathogenicity, this would be tantamount to assuming a stochastic pathogenicity mechanism where sometimes a microbiome is responsible for AD and sometimes not. It might be reasonable if we were observing AD-control mixes of 80–20 or even 70–30 where we might be able to speculate that they derived from individual differences such as immune system capability. The AD statistics for blue, orange, and red were, however, just too close to 50–50 for comfort. Furthermore, since we were unable to split the color clusters through parameter adjustment, we were confident in their robustness and therefore their microbiomes. Therefore, we needed to find a more parsimonious explanation than accepting stochastic pathogenicity. We found one in the subject color class statistics. It is important to remember that the class results emerge from an analysis of bacterial data only. The disease statistics from the inset of **Figure 5** are a statement of results only, i.e., which samples in each class came from AD or control brains and are not computed by fitting an *a priori* hypothesis.

##### Subject color class statistics

**Figure 11:**
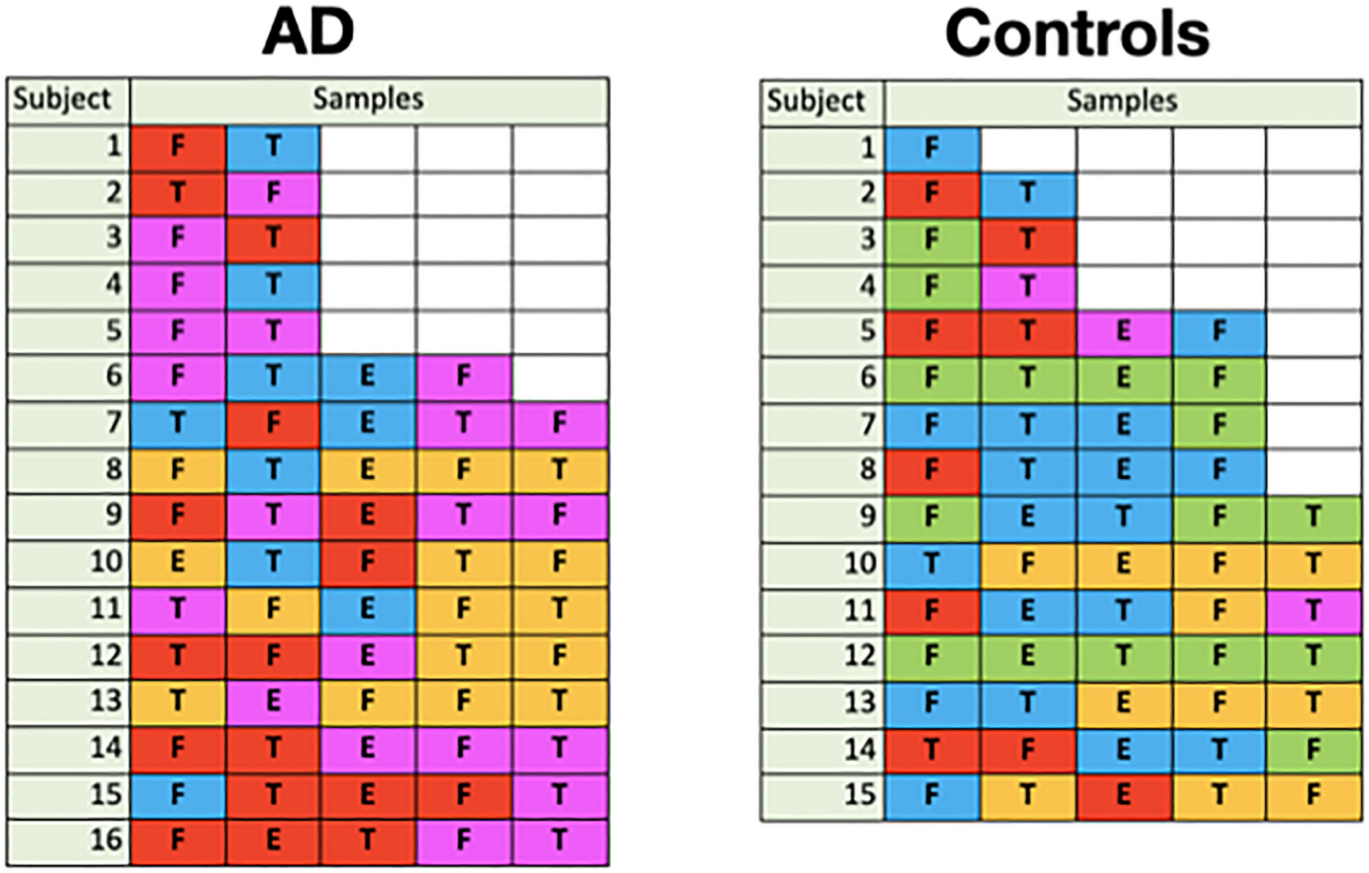
Color class of samples by subject (F: frontal lobe, T: temporal lobe, E: entorhinal cortex).

Because the disease state variable is an attribute of a subject, not a sample, it was necessary to relate the subjects’ sample color class distributions to disease state in order to find a relationship between class and AD. In **Figure 11**, we display the color class of each sample for all the subjects.

**A glance at this figure indicates that the occurrence of a single magenta sample accounts for a subject’s AD in almost all cases**

This suggests that the ecosystems underlying the other classes are not pathogenic even though many of their samples come from subjects who have AD. In order to do a more rigorous computation, we defined a subject-level classification as the normalized sum of the classifications of each of its samples. The sum was unweighted as we had no *a priori* way to assign weights. Using the resultant mixture vectors as independent variables in a logit regression with a cutoff of 0.5 (Wolfram Research Inc, 2008), we were able to obtain a high accuracy prediction (about 88%) of AD or lack of AD. True Positive, False Positive, True Negative, and False Negative rates were found to be 88%, 13%, 87%, and 13%, respectively. See the **Supplementary Methods** for additional details.

##### Location

There are insufficient data to say anything definitive about location, but we offer one set of results. There is a hint that when a magenta infection occurs in an AD subject, it is likely to be found at least in the frontal lobe. Out of 13 AD subjects with magenta infections, nine have at least one frontal lobe magenta sample whereas only two have temporal lobe magenta samples without a frontal lobe magenta sample. Of these 13 AD subjects, five have temporal lobe magenta samples that also have magenta frontal lobe infections. For the three subjects that had samples in the entorhinal cortex, only one had samples in the temporal or frontal cortex and, in this case, it was both. The other two had neither.

**Thus, at least with this small set of data, there is a suggestion that subjects with the magenta class in their frontal lobe are more likely to have the diminished cognitive abilities that result in AD diagnoses.**

## Discussion

The existence of a human brain microbiome has been suggested recently (Branton et al., 2016; Emery et al., 2017; Link, 2021), and a dysbiotic brain microbiome could contribute to AD pathogenesis. The use of long-read sequencing of full-length 16S rRNA genes allowed us to profile the bacterial composition of human brain tissue samples from AD and non-demented control subjects as well as to evidence potential complex polymicrobial interactions.

### Spatiotemporal and pathogenic relationships as evidence supportive of a possible bacterial etiology of AD

The DMM and MLDA computations provide us with a rich set of results that allow us to extract multiple patterns relating to AD. DMM succeeded in finding what was not revealed by using the usual frequentist tests. By properly treating the compositional nature of the data, it rigorously revealed those taxa that are the most important in the two cohorts and thus requiring further investigations in terms of space and time. The interpretation of the MLDA computations has led us to surmise how Alzheimer’s disease may develop because of dynamic bacterial ecosystems in the brain, although other microbial or non-microbial factors may also be simultaneously involved (Hu et al., 2022). We discuss how these ecosystems are arranged microscopically and how they are spatially distributed over the brain, using the color classes as surrogates for time or phase of disease development to reveal spatial and temporal microbiome patterns related to AD.

The patterns and relationships obtained from our analyses constitute evidence supportive of a possible bacterial role in the etiology of AD. Even so, considerably more work will be required to establish proof of such a role. In particular, we do not yet know where these bacteria are located with respect to the brain’s physiological architecture, whether other non-bacterial microbes are involved, or whether they are causing damage or are only markers for physiological changes that they did not cause. Even so, while there are no standard statistical tests for all of what we have found, it seems unlikely that these patterns occurred by chance.

#### Role of *Cutibacterium acnes*

*C. acnes* occurs at some level in 83% of the samples, both the AD and controls, and in all classes. It occurs in over 88% of the samples not including the green class. These observations suggest that it may be interacting with all the ecosystems in each class and through these interactions plays a primary role in defining class by determining which microbes ultimately predominate. If the temporal order of classes we have argued is correct, *C. acnes* begins at low abundance as seen in the green class, which then increases in abundance in the orange class, peaking in the blue class and falling in abundance throughout the red and magenta classes as disease emerges. The fact that orange and magenta have similar average abundances strongly suggests that, as time passes, something changes, perhaps physiologically, to allow the M+ species complex to dominate in the magenta rather than the ecosystems evolving back to orange as *C. acnes*’ abundance falls. Perhaps the brain’s immune protection is diminished or a failing blood–brain barrier gradually increases the microbes it lets in over time. This may be an example of multiple meanings of one object mentioned earlier where the same *C. acnes* objects in the orange and magenta classes are involved in different processes.

*C. acnes*’ ubiquity may correlate with another well-known observation, the ubiquity of plaques and NFTs in the brain tissue of both AD and non-AD patients. We do not have Braak stage data for the age-matched controls to test this hypothesis. We suggest that the ubiquity of both is not happenstance and perhaps the plaques and tangles are some type of response to the *C. acnes*.

We emphasize that our results suggest that the presence of *C. acnes* alone is not evidence of damaged tissue that results in the observed cognitive impairment of the subjects. In other words, we are suggesting that the presence of *C. acnes* could cause plaques and NFTs but *not* AD. Even so, the evolution of the microscopic structure of *C. acnes* ecosystems (see below for discussion of microscopic structure) suggests that it is a driving factor in the emergence of AD, even if it does not directly cause it. While the *C. acnes* ecosystems are a little closer together in the orange than in the green, it is enough to eliminate the ability of *C. jiangduensis* to survive. As the concentration of *C. acnes* in ecosystems increases, the ability of *A. junii* to survive diminishes, suggesting its lack of a role in pathogenesis. Clearly, in the magenta class, something dramatic changes as the microscopic homogeneity of *C. acnes* declines along with its abundance.

#### Contamination

We did have a concern about contamination by *C. acnes* and *Acinetobacter* given their prevalence on the human body and in the environment, respectively. We do not believe there to be a problem for three reasons. First, we presented findings in Data Filtering above that they were not. Second, they did not appear in the negative controls. Third, if objects are from contaminants, they should affect all classes the same and have a class distribution with roughly equal components. This is far from the case with these bacteria whose objects had components with high values in some classes.

#### Pathogenicity

We have argued that the only class that has a strong relationship to pathogenicity is the magenta class. Given that there are many M+ bacteria from many species and genera, it is hard to argue that these are all pathogens. Their presence in the magenta class and their pairing with *C. acnes*-(11-13) in individual samples suggest that the presence of both is related to pathogenicity, although it is possible that the M+ are pathogenic alone and occupy spatially distinct niches, with their presence driving down the Cu abundance. From a biological point of view, this pairing suggests some type of interaction between the *C. acnes* and M+. It is not outlandish to presume that the M+ share how they communicate or compete even though they are demonstrably different species (Prindle et al., 2015; Andrew et al., 2021). Thus, it may be that the biochemical mode of communication or other interaction between M+ and *C. acnes* directly causes AD. Alternatively, there may be something physiological that changes to allow the M+ and *C. acnes* to coexist. The microscopic ecosystem structure that we present below allows us to further characterize this interaction. Those computations suggest that the *C. acnes* and the M+ are part of separate polymicrobial clusters so these clusters are involved in whatever interactions that may exist.

#### Microbiology of principal bacteria

As we stated at the beginning of the paper, it is difficult to ascertain the behavioral properties of all the bacteria observed including the principal ones. We will nonetheless try to point out how some of these properties may be consistent with what our results show. We will focus on their motility and preferred pH. We will not comment on the M+ set. We understand that microbes other than bacteria could be involved but did not attempt to observe fungi, viruses, or other microorganisms. That will be the subject of future work.

*C. acnes* is not motile (Vorobjeva, 1999; Mayslich et al., 2021), while *A. junii* has twitching motility (Bitrian et al., 2013; Jung and Park, 2015) and *C. jiangduensis* is motile (Steinberg and Burd, 2015). *C. acnes*’ lack of motility suggests that there must be a mechanism for its ubiquity other than the ability to move. Perhaps it gains access through the capillaries of the blood–brain barrier or another system like the glymphatic system. *A. junii* has limited motility, suggesting a somewhat similar mechanism.

*C. jiangduensis*’ association with control subjects suggests that it might be part of a healthy microbiome or at least the microbiome of elderly subjects without AD. As it is motile, perhaps it functions efficiently in the intercellular medium as a waste processor. While it is not a major gut bacterium, its biofilms proliferate in human wastewater treatment facilities (Wu et al., 2015; Wu et al., 2018) suggesting it may prefer the pH found there of 7–9. *C. acnes*’ ability to emit propionic and acetic acids (Mayslich et al., 2021) may make it difficult for *C. jiangduensis* to thrive or live. This type of mechanism provides for a long-range mechanism to reduce *C. jiangduensis* if the *C. acnes* and *C. jiangduensis* ecosystems are not close. Furthermore, if *C. jiangduensis* has a waste treatment function, its elimination could result in pathologies. *A. junii* also prefers a pH of 7–9, again suggesting a mechanism for anti-correlation with *C. acnes* (Jung and Park, 2015).

It is not clear if the lower levels of *C. acnes* in the magenta class are due to competition with M+, an increase of M+ in niches not occupied by *C. acnes*, or other factors.

Last, given our suggestion that *C. acnes*, *A. junii*, *C. jiangduensis*, and M+ may occupy distinct spatial niches, the question arises as to where these niches are. One group imaged brain tissue from ALS patients and found inter- and intracellular bacteria as well as fungi, which is consistent with our prediction of distinct spatial niches (Alonso et al., 2019). Most importantly, this group observed bacterial abundance profiles in these subjects that had key similarities to the magenta microbiome. Specifically, many samples had levels of *Methylobacterium* that were several times higher than the *Cutibacterium* they found. This, of course, suggests a multi-disease effect for bacterial infection, although the common theme reported among degenerative brain diseases is buildup of toxic protein breakdown products.

#### Points of entry—blood–brain barrier

Much has been written about the possibility of AD being a vascular disease involving the failure of the blood–brain barrier (BBB) (Sweeney et al., 2018). While less is known, other distribution systems like the glymphatic system could also be candidates (Iliff et al., 2012) as well as nervous networks.

The major reason why fluidic distribution systems could be behind our results is that there needs to be a mechanism for the random microscopic distribution of ecosystems and macroscopic distribution of class mixtures that we detail below (BBB will be used to refer to all networks from here on). The BBB could provide such a mechanism because it could deliver bacteria anywhere in the brain—if and as it fails. This process could be essentially a pseudo-random scattering of bacteria into the capillaries and across them into cells and beyond. On the other hand, if the BBB fails in only some localized places, we would need a spreading mechanism inside the brain to distribute the bacteria. Spreading, however, is more likely to cause a regional structure of ecosystems, which is contradicted by our computations below. While we suggested that the orange class could have a regional structure, this appears to occur early and is not associated directly with the illness. There are reports, however, that it happens (Stopschinski et al., 2021). It may be though that it is the failure of the BBB that spreads spatially, masquerading as a spreading infection (Pritchard et al., 2022).

The hints at class diversity between lobes that we have mentioned where the frontal lobe seems more associated with AD than the temporal lobe further suggest that we are observing a gradual failure of the BBB by lobe. A worsening of the failure could also account for both the rise and fall of *C. acnes* abundances. Perhaps, on the ascendant side of the *C. acnes* abundance curve, the BBB is not in as bad shape and lets through the *C. acnes* and *A. junii* ecosystems without cognitive symptoms. As the pathology worsens, maybe the BBB allows more of the M+ set in and they can either outcompete the *C. acnes* or occupy a different niche outside of *C. acnes* influence where they can increase in abundance and do the damage associated with AD cognitive symptoms. If large-scale BBB failure happens in AD, bacterial introduction to the brain through a failure in the BBB could be at the root of other neurological diseases but involve failure in other parts of the brain.

There is also an alternative to this bacterio-centric picture, or at least another pathogenic mechanism that might coexist with it. Bacteria may just be markers for the progressive failure of the BBB that is allowing another pathogenic microbe such as a virus or fungus or some other molecule to enter the brain concurrent with the development of the magenta stage. The latter would indicate that the complex dynamics that we report are actually temporal markers for the gradual failure of a brain blood or lymph distribution system. This certainly is always worth keeping in mind, especially with the increasing evidence of the presence of fungi and viruses in the brain (Itzhaki et al., 2020; Li et al., 2021). Even so, it is quite hard to conclude that all of the bacterial patterns are unrelated to the cause of AD. Further research could illuminate which possible mechanisms exist and whether the location and type of infection explains other neurological diseases.

#### Ecosystem mixtures—from microscopic to macroscopic

Since the sampling process sums and averages the bacterial load over the sample volume, the sum does not tell us about microscopic structure within a single sample directly. It comprises a bulk macroscopic measurement. There could be ecosystems inside human cells, between the cells, within capillaries, etc., each with different bacterial abundances. The same bacteria may be in more than one ecosystem. In other words, there are lots of possibilities, but the sampling process just sums them all giving us a set of total abundances for each sample. In other words, a class microbiome comes from a sum of the sample’s ecosystem mixtures.

While we did not observe the bacterial ecosystems like a microscope, the MLDA results give us enough to identify important features. We will use the differences among samples *within* a class to infer spatial features of the underlying ecosystems. Specifically, we will use the abundance distributions of the principal bacteria and their occurrence within each class to reveal the microscopic structure. Since the results already tell us that the magenta class is associated with AD and its lack mainly not associated with AD, the underlying structure of this class should provide additional information with regard to how its bacterial ecosystems could be causing AD.

At the other end of the spatial scale, we wish to understand how the classes of ecosystem mixtures are spatially distributed in the brain. We will compare the large-scale spatial distribution of sample color classes to simulated spatial distributions of ecosystem mixture classes to look for such patterns.

### Cellular-scale microscopic structure

#### Spatial sampling—simplifying assumption

The classes are dominated by three genera: *Cutibacterium (P)*, *Acinetobacter (A)*, and *Comamonas (C)* and, in particular, three species, *C. acnes*, *A. junii*, and *C. jiangduensis*, which have both high abundance within a sample and a high prevalence among the samples of a class. We will assume that classes represent different mixtures of ecosystems that the MLDA algorithm separates and that it is reasonable to apply the virtual sampling principle of Theme 2 in the Results (not to be confused with the virtual brain). A physical sample is the weighted sum of these class mixtures where the weights are the class components. The principle allows us to model a single physical sample as if it were the separate sampling of each ecosystem mixture class (see for example **Table S3**, **Table S4**, **Table S5**, and **Table S7**). Given that the maximum class components in most samples are high (see Results—Theme 2), it is reasonable to approximate the sample as if it contained a single component defined by its color (maximum component).

#### Idealized microscopic model of ecosystems

**Figure 12:**
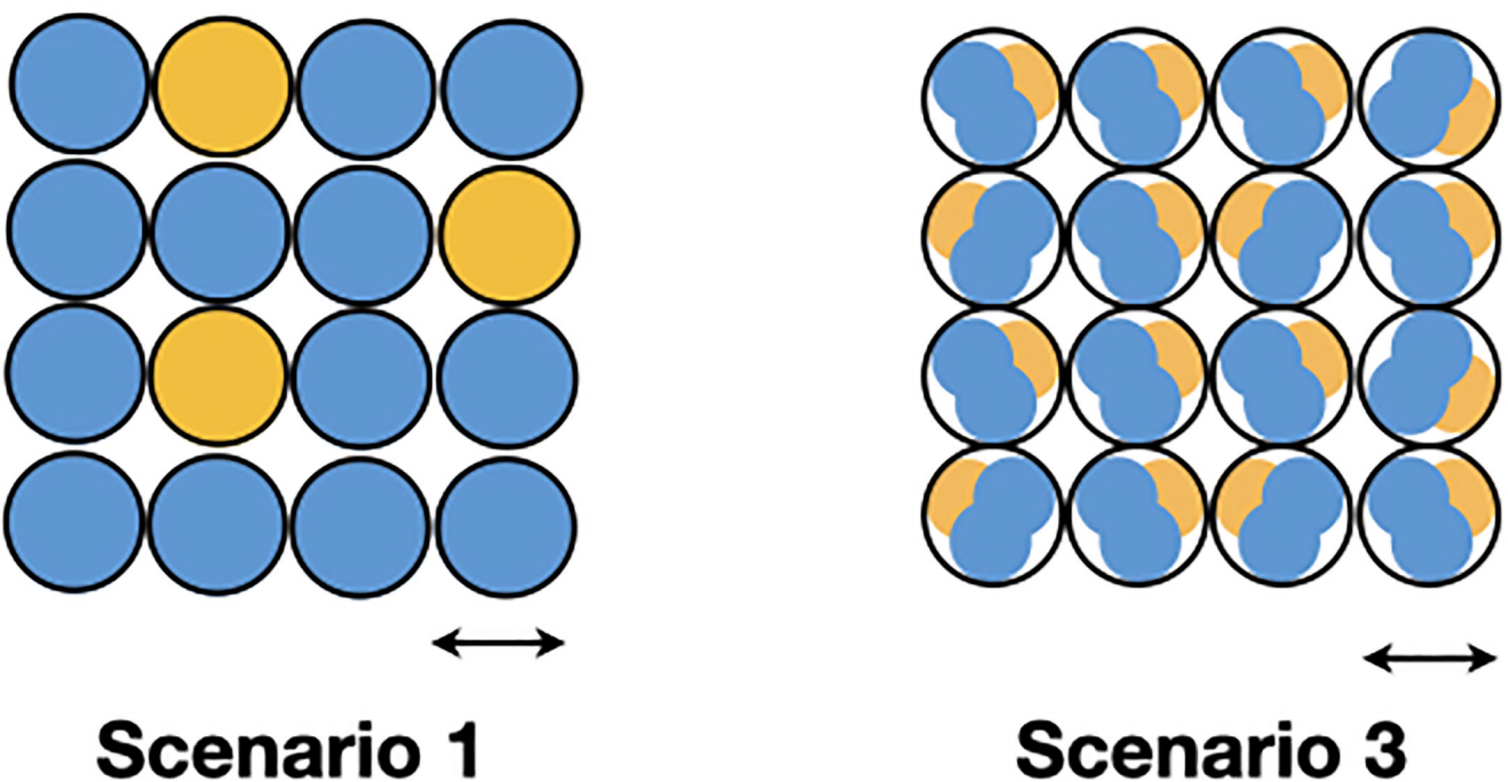
This figure shows possible ecosystem structure at the microscopic scale. The arrows roughly indicate the human cellular scale. Scenario 1 suggests ecosystems dominated by one principal bacterium predominate around a particular cell, whereas scenario 3 suggests that ecosystems composed of multiple principal bacteria predominate around a particular cell. A physical sample would comprise all or large fractions of the above arrays.

**Figure 13:**
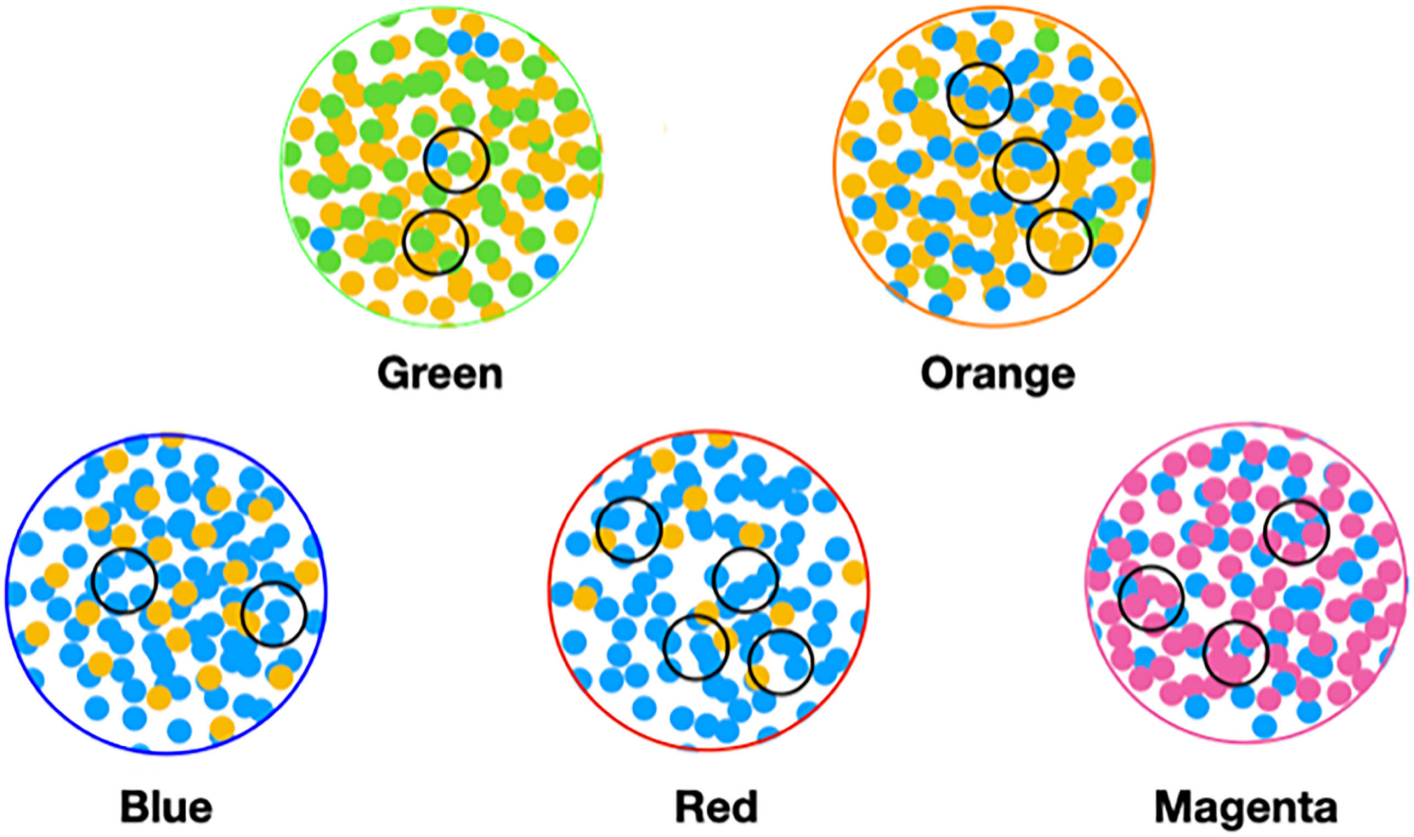
Idealized depiction of distribution of ecosystems. Each dot is an ecosystem dominated by a particular species: blue for *C. acnes*, orange for *A. junii*, green for *C. jiangduensis*, and magenta for M+. The large circles are class mixtures also labeled by colors. The small black circles depict samples. Green is dominated by *C. jiangduensis*; blue is dominated by *C. acnes*; orange by *A. junii*, and magenta by the M+ set.

There are several models that could account for the measured data. We present the following one as it is sufficiently general to encompass many and is parsimonious. It is an interleaving model that assumes that there are distinct polymicrobial ecosystems that exist at the human cellular scale, each being dominated by one of the principal/dominant bacteria. Refer to **Figure 12**. One can visualize them as pixels that aggregate to a sample. Each pixel represents a *Cutibacterium*, *Acinetobacter*, M+ or a *Comamonas*-dominated ecosystem. Each class has its own unique arrangement of these ecosystems that can manifest different densities and homogeneities within the brain. In some cases, one pixel may be spread over the sample area with a random scattering of one or two of the others. In other cases, there may be two with similar densities and a scattering of the third. The scattering densities vary by class. In general, low densities imply a larger average distance between ecosystems and high densities imply a smaller distance (**Figure 13**). Add up the number of ecosystem pixels within a black circled sample as an estimate of the measured abundance of the raw data in a particular class. Densities and homogeneities of each ecosystem pixel will determine the abundance averages and distributions within the class upon repeated samplings by randomly moving the black circle around the displayed region. Lower-density pixels will tend to be less homogeneous and have wider abundance distributions than higher-density pixels. Note our use of “density” to describe actual biological distributions as opposed to “abundance,” which we use to describe the measured fractional amount of a bacterium within a macroscopic sample.

Given that the sample to human cell size ratio is on the order of 50–100×, it is possible to qualitatively reproduce the class raw data, by experimenting with different densities and homogeneities of the pixel distributions. While we know that these pixels representing ecosystems are small, we are not able to compute a precise size or shape with the information we have. Closer physical sampling would help, but direct microscopic observation is needed to confirm actual sizes and densities. Even so, if we can infer a particular arrangement of the principal bacterial ecosystems, we will obtain a sense for their relative distances.

#### Idealized microscopic model of ecosystems—class details

These descriptions show how small discrete polymicrobial clusters or ecosystems can explain the bulk data for each class. The less abundant and less prevalent microbes are assumed to be part of the ecosystems because the MLDA results depended on them although we discuss them in terms of their dominant microbe.

In **Figure 13**, each large circular area is an idealized area of the brain representing a pure ecosystem mixture class. Each smaller black circle is a physical sample. Each dot or pixel is a polymicrobial ecosystem with a particular dominating microbe: blue for *C. acnes*, orange for *A. junii*, green for *C. jiangduensis*, and magenta for M+. The number of dots within a sample is a qualitative way of estimating the abundance of the principal microbe. By showing gaps in some of the spatial distributions, we are trying to create a visualization of inhomogeneity. Below, we describe how particular ecosystem structures could produce the results from **Table 4**. For each class (a), we first characterize the distributions from **Table 4** and then (b) describe a polymicrobial ecosystem arrangement (pictured in **Figure 13**) that could produce these distributions upon repeated samplings described above.

Green. (a) The green class has *C. jiangduensis* and *A. junii* as its dominant microbes. There is a lot of *C. jiangduensis* in the green class. Green presents with a narrow *C. jiangduensis* distribution of 14s and 13s. *A. junii* has a narrow distribution of 13s and 14s. *C. acnes*, on the other hand, has a wider distribution peaking at 11 with a width of several abundance units and is only present in half of the samples at levels ≥7. (b) These results suggest an ecosystem structure where *C. jiangduensis* and *Acinetobacter* spp. are interleaved with one another at high density with a random scattering of *C. acnes* at lower density than either *C. jiangduensis* or *A. junii*. This density should be low enough so that there is a high probability that some samples from a green region will not contain *C. acnes* as observed.

Orange. (a) The orange class has a narrow distribution of *A. junii* in 13s and 14s that it shares with green. In comparison with green, the *C. acnes* distribution is narrower with a pronounced peak at 13 and a presence in nearly all samples. *C. jiangduensis* has a wide distribution but is present in less than one-third of the samples in this class and has no samples with abundances at 13 or 14. Compared with the green class, it has largely disappeared. (b) These results suggest a high-density distribution dominated by *A. junii* with a significant density of *C. acnes* but with *C. jiangduensis* present only at a very low density. The fact that the *C. acnes* distribution has a width >2 and its abundance level is not the highest suggests some inhomogeneity in its spatial distribution.

Blue. (a) The blue class has a very narrow distribution of *C. acnes* with a high average abundance at 14 that is representative of almost every sample of the class. *A. junii* has a wide distribution, peaking at 10, suggesting a competition where *C. acnes* has become dominant. *C. jiangduensis* is not present at all in abundances ≥7. (b) Because of the narrowness of the *C. acnes* distribution and high abundance, its spatial distribution should be homogeneous with high density with a light random scattering of *A. junii.*

Red. (a) The red class has a somewhat wider *C. acnes* distribution than the blue class with some 13s in addition to 14s. There is again no *C. jiangduensis*. (b) The larger width of the *C. acnes* distribution compared with the blue class indicates that the underlying spatial distribution of its ecosystems is not as homogeneous nor as dense as in the blue class. The *A. junii* distribution is wide, but it does not occur in most of the samples suggesting it is widely spaced with significantly lower densities than in the *A. junii-*predominant green and orange classes.

Magenta. (a) The magenta class has a wider *C. acnes* distribution compared with blue with a mix of 13s, 12s, and a few 11s leading to lower average abundances of this species. Again, there is no *C. jiangduensis* ≥level 7 abundance. As mentioned in the results, the M+ microbes appear along with the *C. acnes* within individual samples, but at far higher abundances. Looking back at the M+ microbes in earlier classes, we see that they have been present, however, at far lower abundances with wider distributions, roughly in the 8–12 range. Importantly, in the red and magenta classes, they jump up into the 13–14 range where in most samples they overtake the *C. acnes* abundances. (b) The *C. acnes* ecosystems are therefore not homogeneously distributed due to the 11–13 range abundances. A distinct *C. acnes* ecosystem assumption has worked in the previous four classes, so we will continue to assume this here and now also assume that the M+ are also distinct. In future work, however, it should be considered that *C. acnes* and M+ are part of the same ecosystem. In other words, in overtaking *C. acnes* in the magenta class, the M+ microbes could either have increased their abundances in microscopic niches where *C. acnes* was not located, or they may have outcompeted *C. acnes* in their own microscopic niches within a single ecosystem. Using the former assumption leads to a homogeneous high-density M+ arrangement interleaved with an inhomogeneous *C. acnes* arrangement at lower density.

#### Microscopic structure summary

The analysis above suggests a structure much smaller than the sample size that explains the MLDA results, showing how to qualitatively reproduce their abundances and their variances with a sampling process. We cannot determine the size of these interleaved ecosystems at this point, but the model suggests that they are spatially distinct with different dominant microbes and relative distances that are class dependent. Given that the brain’s tissue architecture is roughly the same from sample to sample, their spatial arrangement is likely driven by different biological niches; the architecture of the brain’s vascular, lymphatic, or nervous networks; and processes that randomize their location. Last, because a particular spatial arrangement is associated with a color, the arrangement is related to a particular stage in the development of Alzheimer’s disease.

### Large-scale macroscopic structure

We explored how the ecosystem mixture classes were distributed spatially, as this might suggest how the bacteria travelled to their measured locations. Our metadata includes anatomical brain locations (e.g., lobe), which is not the same thing as a geometric location. We therefore took a statistical approach to location, using the concept of the virtual brain. Recall that the virtual brain idea is to assume that the samples came from two subjects, one for each cohort, increasing the sampling density from ~4/subject to about ~60/subject. This is different than just compiling averages over each cohort because we are assuming that the samples actually sampled only two brains and that measurement variances are due purely to spatial distributions. The following analysis is distinct from our earlier finding that the magenta class samples of AD subjects are mainly found in the frontal lobe. There, we simply reported counts by lobe of sample color class occurrence. In the following analysis, we are trying to determine how different simulated spatial arrangements of color classes can reproduce the data at the subject level.

**Figure 14:**
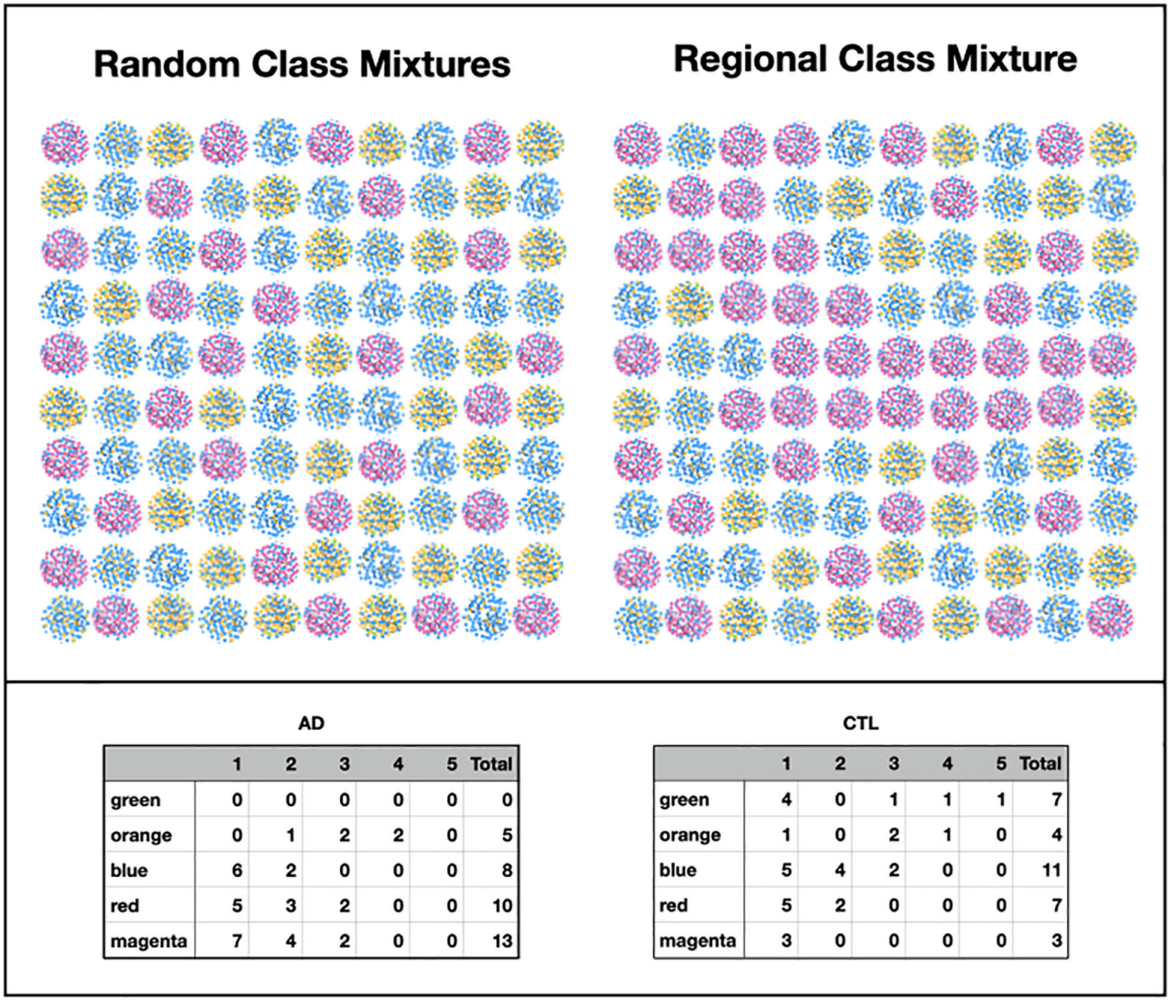
Each array represents a large area of the brain. Each element is a single class mixture like the ones from **Figure 13**. The size of the element could be from centimeters to several centimeters. The left-hand side produces statistics like the inset below (except orange). The right side produces flatter statistics without 1s, like orange. The inset contains the number of class occurrences by subjects for each class for comparison with simulation of macroscopic distribution scenarios.

We constructed statistics from the actual class results summed over subject to create a virtual brain. We interpreted these statistics by comparing them with the same statistics of simulated spatial distributions. We used two simulated scenarios detailed below. For the actual results statistics, we constructed distributions of the number of class occurrences by subject for each class. In other words, we counted the number of subjects that have one occurrence of a class, the number of subjects that have two, etc. See the bottom inset of **Figure 14**. In all colors and disease states but one, orange, we see a skewing toward the occurrence of one class. In the orange case, both the AD and control distributions are skewed toward a flatter broader distribution that does not include 1s.

The first comparison scenario used random class mixtures in a plane with magenta having the highest probability of occurring (LHS of **Figure 14**), to be consistent with its occurrence in the AD subjects (see **Figure 11**). The second scenario clumped the magenta over a large region (RHS of **Figure 14**). We repeatedly randomly undersampled each like we did with the real brain experiments, sampling four at a time, and constructed the same statistic as for the real results above.

The random scenario produced results skewed toward one occurrence among four colors. The regional structure, once it is large enough, produces a flatter distribution that is missing 1s and distributed over 2s, 3s and 4s. Our results, except for orange, are therefore consistent with the *lack* of a regional structure. Given the amount of data, it is hard to say much more than the orange data suggests more of a regional structure, but it is, nonetheless, different.

#### Macroscopic structure summary

Therefore, given the high occurrence of magenta in the AD subjects, we can say that there is a high random density of small areas in these subjects that contain the magenta microbiome interspersed with the others. We do not have sufficient data to determine their actual size. In addition, if our comment about orange is right, some of the subjects might have large regions that are orange with scatterings of the other colored microbiomes. Overall, there is a high degree of randomness that we infer throughout our results. Different classes of ecosystem mixtures are randomly distributed over the brain with magenta having a higher probability in the AD brain. Since class is associated with time and disease stage, some areas are at an earlier stage and others at later stages.

## Conclusions

We were able to ascertain abundance differences of specific bacteria comparing AD and control cohorts. Using a separate methodology, we were also able to distinguish multiple microbiomes in both cohorts, defined by sets of bacteria within specific abundance ranges. While these methodologies were different, they were consistent.

We described spatiotemporal changes in these microbiomes from microscopic to macroscopic spatial scales. Specifically, we uncovered an evolving microbiome in the human brain that begins, perhaps as a healthy microbiome, and then gradually changes until it is unquestionably associated with Alzheimer’s disease. These patterns can be interpreted as evidence for a bacterial etiology of AD, although proof of their role will require further investigation, especially an examination of the role of other microbes.

The earliest of these microbiomes, found in the control cohort, is dominated by *Comamonas* spp. and could be part of the healthy microbiome. The latest of these microbiomes is characterized by two microbes, *Cutibacterium acnes* and another bacterium most often *Methylobacterium* spp. Their co-occurrence in the brains of the AD cohort, primarily in the frontal lobe, suggests possible pathogenicity. The identification of a very similar microbiome in ALS patients (Alonso et al., 2019) raises the idea of common pathogenic factors in the diseases.

*C. acnes* is ubiquitous in the samples and found in both cohorts. It begins at a low abundance, rises, peaks, and falls off when it is joined by *Methylobacterium* in the possibly pathogenic microbiome. This dynamic and its ubiquity suggests that the formation of plaques and NFTs could be a reaction to its presence in both non-demented and AD-demented individuals. The co-occurrence of *C. acnes* and *Methylobacterium* from the AD-correlated microbiome suggests that pathogenicity may be related to an interactive process of some kind.

The large-scale spatial distribution of most of these microbiomes appears to be random without large regional clustering of microbiomes. The randomness extends to the microscopic scale where there is evidence that the most abundant bacteria arrange to form separate polymicrobial clusters. The randomness could be explained by microbial transport to the end of the brain’s vascular, lymphatic, or nervous networks, and the dynamics of the failure of one or more of them could be involved with the evolution of the pathogenic microbiome and the onset of Alzheimer’s disease.

There is an alternative to this bacterio-centric picture, or at least another pathogenic mechanism that might coexist with it. This mechanism may involve other pathogenic microbes such as viruses or fungi or some other molecule that enters the brain concurrent with the development of the *C. acnes* and *Methylobacterium* microbiome. The latter would indicate that the dynamics that we found are actually temporal markers for the gradual failure of the brain’s networks. Further research could illuminate the type of failure and whether the location and infection characteristics explains other neurological diseases.

This bulk microbiome dynamic, however, calls out for explanation in more fundamental terms. It is a complex dynamic that likely involves the time dependence of multiple interacting systems: including the microbial ecosystems, a changing immune reaction with genetic constraints, and dynamic delivery networks driven by external factors that could have happened once or are ongoing. This investigation has only begun to uncover how this works. Understanding AD apparently will involve a program of examining these fundamental components, how they affect each other, and ultimately how they affect the function of the mind.

## Data availability statement

The full-length 16S sequences have been deposited at the NIH NCBI SRA repository (BioProject PRJNA822777). Additional code and input and output files related to each analytical method are shown below.

## Ethics statement

The studies involving human participants were reviewed and approved by the Institutional Review Board (IRB) of Drexel University College of Medicine (IRB approval # 1410003161). The patients/participants provided their written informed consent to participate in this study.

## Author contributions

GDE and YM conceived and designed the study. AA, BS, GDE, JK, JE, and YM provided the materials and reagents and carried out the experiments. JL coded and wrote the software for the method to analyze combinations of bacteria. GDE, JL, and YM analyzed and interpreted the data. GDE, JL, and YM wrote the manuscript. All authors read and approved the final manuscript.

## Supplementary material

Supplementary Table S1 OTU identified as potential contaminants.

Supplementary Table S2 Taxonomic classification of OTU that shift in abundance between the Alzheimer’s disease group and the age-matched control group. For each OTU of interest, the taxonomic assignment and the family-, genus- and species-confidence values are reported.

Supplementary Table S3 Classification results with lower occurring objects summed over abundance bin.

Supplementary Table S4 Classification results without summing lower occurring objects over abundance bin.

Supplementary Table S5 Classification results without summing lower occurring objects over abundance bin after object merging.

Supplementary Table S6 Main Microbial Objects By Class and Disease State - Individual Samples.

Supplementary Table S7 Microbiome objects approximated from sample input data of a given color - counts.

SUPPLEMENTARY FIGURE S1 Differences in relative abundance between the Alzheimer’s disease (AD) group and the age-matched control group (controls). The relative abundances were estimated for each OTU from each group through hierarchical Bayesian modeling while ignoring the non-independence of the samples. The vertical axis shows the difference for the estimated relative abundance of OTU between the AD and control groups. Points are the means of PPD and the whiskers show the 95% equal tail probability intervals of PPD (see Materials and methods).

SUPPLEMENTARY METHODS Details of mathematical methods.
