# Supplementary material for: Evidence supportive of a bacterial component in the etiology for Alzheimer’s disease and for a temporal-spatial development of a pathogenic microbiome in the brain
Source: Front Cell Infect Microbiol. 2023 Sep 13;13:1123228. doi: 10.3389/fcimb.2023.1123228 (PMC10534976; doi:10.3389/fcimb.2023.1123228)

 10.3389/fcimb.2023.1123228

Supplementary Material (Tables S1-S7, Figure S1, Supplementary Methods in separate file)

Table S1: OTU identified as potential contaminants
OTU identifiers (column 1), species name (column 2), Genus-level confidence values (column 3), Species-level confidence values (column4), Mean of relative abundance (RAb) in negative controls (column 5), Mean of relative abundance in biological samples (column 6), Prevalence-based score statistic P (column 7). OTU are identified as contaminant (TRUE) when they show a score statistic P > 0.5 or when RAb in negative controls is higher than RAb in biological samples (column 8).

**OTU_ID**	**species**	**genus conf**	**species conf**	**Rab in negative controls**	**Rab in samples**	**decontam statistic score *P***	**contaminant**
OTU_771	*Achromobacter denitrificans*	0.409	0.034	0.037	0.001	0.995	TRUE
OTU_2	*Achromobacter xylosoxidans*	0.829	0.494	16.688	2.530	0.800	TRUE
OTU_555	*Achromobacter xylosoxidans*	0.470	0.044	0.012	0.000	0.996	TRUE
OTU_566	*Achromobacter xylosoxidans*	0.348	0.024	0.002	0.001	0.962	TRUE
OTU_650	*Achromobacter xylosoxidans*	0.530	0.044	0.022	0.000	0.974	TRUE
OTU_9	*Acinetobacter junii*	0.973	0.935	0.001	9.810	NA	FALSE
OTU_3	*Anabaena cylindrica*	0.002	0.000	14.117	0.812	0.847	TRUE
OTU_549	*Anabaena cylindrica*	0.002	0.000	0.002	0.000	NA	TRUE
OTU_295	*Bordetella petrii*	0.829	0.719	0.002	0.028	0.974	TRUE
OTU_916	*Corynebacterium mucifaciens*	0.953	0.054	0.004	0.000	0.974	TRUE
OTU_103	*Corynebacterium ureicelerivorans*	0.986	0.306	0.311	0.114	0.772	TRUE
OTU_1	*Cutibacterium acnes*	0.973	0.958	0.001	22.043	NA	FALSE
OTU_8	*Delftia acidovorans*	0.980	0.599	0.001	2.243	NA	FALSE
OTU_467	*Hydrotalea flava*	0.876	0.306	0.009	0.001	0.995	TRUE
OTU_6	*Hydrotalea flava*	0.980	0.840	23.986	12.350	0.500	TRUE
OTU_729	*Hydrotalea flava*	0.288	0.014	0.028	0.026	0.859	TRUE
OTU_1060	*Hydrotalea sandarakina*	0.050	0.003	0.009	0.018	0.859	TRUE
OTU_1099	*Pelomonas puraquae*	0.470	0.044	0.002	0.000	NA	TRUE
OTU_1054	*Pelomonas saccharophila*	0.348	0.054	0.001	0.000	NA	TRUE
OTU_7	*Pelomonas saccharophila*	0.735	0.400	3.590	0.601	0.809	TRUE
OTU_262	*Peredibacter starrii*	0.001	0.000	0.020	0.001	0.998	TRUE
OTU_5	*Peredibacter starrii*	0.001	0.000	21.524	3.155	0.742	TRUE
OTU_835	*Pseudomonas hibiscicola*	0.348	0.034	0.114	0.005	0.975	TRUE
OTU_65	*Sediminibacterium ginsengisoli*	0.050	0.004	0.518	0.375	0.784	TRUE
OTU_1082	*Sphingomonas sanxanigenens*	0.001	0.000	0.001	0.000	NA	TRUE
OTU_36	*Sphingomonas sanxanigenens*	0.001	0.000	0.672	0.011	0.974	TRUE
OTU_436	*Sphingomonas sanxanigenens*	0.001	0.000	0.006	0.000	0.974	TRUE
OTU_1250	*Stenotrophomonas humi*	0.002	0.000	0.001	0.000	NA	TRUE
OTU_4	*Stenotrophomonas maltophilia*	0.688	0.306	18.322	2.288	0.823	TRUE

 10.3389/fcimb.2023.1123228

Table S2: Taxonomic classification of OTU that shift in abundance between the Alzheimer's disease group and the age-matched control group. For each OTU of interest, the taxonomic assignment and the family-, genus- and species-confidence values are reported.

OTU_ID	Family	Species	family_conf	genus_conf	species_conf
OTU_1	Propionibacteriaceae	*Cutibacterium acnes*	0.9909	0.973	0.9575
OTU_111	Oxalobacteraceae	*Noviherbaspirillum soli*	0.7151	0.4696	0.0641
OTU_114	Pseudomonadaceae	*Pseudomonas thermotolerans*	0.9864	0.9235	0.9392
OTU_12	Staphylococcaceae	*Staphylococcus epidermidis*	0.9797	0.9869	0.7194
OTU_14	Comamonadaceae	*Acidovorax temperans*	0.9432	0.9531	0.8396
OTU_15	Comamonadaceae	*Comamonas jiangduensis*	0.8636	0.6407	0.1169
OTU_157	Comamonadaceae	*Acidovorax ebreus*	0.8835	0.5302	0.2112
OTU_166	Moraxellaceae	*Acinetobacter tjernbergiae*	0.8238	0.7821	0.1169
OTU_21	Flavobacteriaceae	*Cloacibacterium normanense*	0.9921	0.9663	0.494
OTU_229	Pseudomonadaceae	*Pseudomonas putida*	0.9898	0.9663	0.5993
OTU_26	Methylobacteriaceae	*Methylobacterium goesingense*	0.9819	0.9862	0.7194
OTU_799	Comamonadaceae	*Diaphorobacter nitroreducens*	0.6006	0.2271	0.0341
OTU_9	Moraxellaceae	*Acinetobacter junii*	0.9631	0.973	0.9346

Table S3: Classification results with lower occurring objects summed over abundance bin.

Green			Orange			Blue	
Comamonas jiangduensis	15		Acidovorax	25		Cutibacterium acnes-14	26
Pseudomonas	12		Cloacibacterium	20		Streptococcus	8
Cloacibacterium	11		Comamonas testosteroni	16		Corynebacterium	8
Moraxella	8		Acinetobacter tjernbergiae-13	13		Sediminibacterium	7
Acinetobacter tjernbergiae-10	6		Acinetobacter junii-13	12		Moraxella	7
Bacteroides	5		Acinetobacter junii-14	11		Achromobacter	7
Acinetobacter junii-14	5		Acinetobacter tjernbergiae-14	9		Kocuria	6
Acinetobacter junii-13	5		Cutibacterium acnes-13	7		Nitrosospira	5
			Zoogloea	5		Acinetobacter junii-10	5
			Pseudomonas	5			
			Novosphingobium	5			
			Comamonas jiangduensis	5			

Red			Magenta	
Cutibacterium acnes-14	14		Cutibacterium acnes-14	14
Nitrosospira	14		Nitrosospira	14
Sediminibacterium	11		Sediminibacterium	11
Cutibacterium acnes-13	6		Cutibacterium acnes-13	6
Kocuria	6		Kocuria	6
Bradyrhizobium	6		Bradyrhizobium	6
			Cutibacterium acnes-14	14
			Nitrosospira	14
			Sediminibacterium	11

Table S4: Classification results without summing lower occurring objects over abundance bin.

Green		Orange (1)		Orange (2)	
Comamonas jiangduensis-14	7	Acinetobacter tjernbergiae-13	13	Peptoniphilus-10	2
Acinetobacter tjernbergiae-10	6	Acinetobacter junii-13	12	Pedobacter-11	2
Pseudomonas-12	5	Acinetobacter junii-14	11	Novosphingobium-12	2
Comamonas jiangduensis-13	5	Acinetobacter tjernbergiae-14	9	Novosphingobium-10	2
Cloacibacterium-12	5	Acidovorax-13	9	Moraxella-11	2
Acinetobacter junii-14	5	Cloacibacterium-12	8	Delftia-12	2
Acinetobacter junii-13	5	Cutibacterium acnes-13	7	Delftia-11	2
Pseudomonas-13	4	Comamonas testosteroni-10	7	Corynebacterium-10	2
Moraxella-11	4	Cloacibacterium-10	7	Comamonas testosteroni-12	2
Lactobacillus-10	4	Acidovorax-11	7	Comamonas jiangduensis-10	2
Cloacibacterium-11	4	Comamonas testosteroni-11	6	Chryseobacterium-11	2
Streptococcus-11	3	Cloacibacterium-11	4	Brevibacterium-12	2
Cutibacterium acnes-11	3	Acidovorax-14	4	Bosea-10	2
Acinetobacter tjernbergiae-11	3	Acidovorax-12	4	Blastomonas-10	2
Stenotrophomonas-10	2	Sphingobium-10	3	Acinetobacter tjernbergiae-12	2
Moraxella-14	2	Cutibacterium acnes-12	3		
Lachnoclostridium-10	2	Cutibacterium acnes-10	3		
Delftia-11	2	Comamonas jiangduensis-11	3		
Comamonas jiangduensis-12	2	Bacillus-10	3		
Bradyrhizobium-10	2	Zoogloea-12	2		
Bacteroides-13	2	Zoogloea-10	2		
Acinetobacter junii-12	2	Streptococcus-10	2		
Acidovorax-11	2	Stenotrophomonas-11	2		
		Pseudomonas-12	2		
		Pseudomonas-10	2		
		Cutibacterium acnes-14	2		
		Cutibacterium acnes-11	2		

Blue		Red		Magenta	
Cutibacterium acnes-14	26	Cutibacterium acnes-14	14	Cutibacterium acnes-13	9
Acinetobacter junii-10	5	Cutibacterium acnes-13	6	Cutibacterium acnes-12	7
Streptococcus-12	4	Sediminibacterium-12	5	Corynebacterium-11	5
Moraxella-12	4	Nitrosospira-13	5	Delftia-14	4
Corynebacterium-10	4	Delftia-11	4	Streptococcus-11	3
Acinetobacter tjernbergiae-10	4	Nitrosospira-14	3	Cutibacterium acnes-11	3
Sediminibacterium-11	3	Moraxella-10	3	Noviherbaspirillum-12	3
Moraxella-13	3	Bradyrhizobium-13	3	Methylobacterium-13	3
Kocuria-10	3	Stenotrophomonas-13	2	Acinetobacter junii-12	3
Bradyrhizobium-10	3	Sediminibacterium-13	2	Variovorax-14	2
Acinetobacter tjernbergiae-12	3	Sediminibacterium-11	2	Variovorax-13	2
Acinetobacter junii-13	3	Schlegelella-12	2	Streptococcus-12	2
Acinetobacter junii-12	3	Ralstonia-11	2	Sediminibacterium-13	2
Achromobacter-10	3	Cutibacterium acnes-12	2	Rhodococcus-11	2
Streptococcus-11	2	Ottowia-11	2	Pseudomonas-11	2
Streptococcus-10	2	Nitrosospira-12	2	Noviherbaspirillum-13	2
Sphingomonas-10	2	Nitrosospira-11	2	Moraxella-11	2
Sediminibacterium-12	2	Nitrosospira-10	2	Methylobacterium-14	2
Sediminibacterium-10	2	Meiothermus-13	2	Methylobacterium-12	2
Nitrosospira-11	2	Meiothermus-12	2	Comamonas nitrativorans-11	2
Microlunatus-12	2	Kocuria-14	2	Caulobacter-14	2
Microbacterium-10	2	Cloacibacterium-13	2	Brevundimonas-12	2
Meiothermus-11	2	Bradyrhizobium-14	2	Bradyrhizobium-10	2
Kocuria-12	2	Blastomonas-10	2	Bacillus-14	2
Corynebacterium-13	2	Acinetobacter tjernbergiae-12	2	Bacillus-12	2
Corynebacterium-11	2	Acinetobacter junii-11	2	Actinomyces-13	2
Acinetobacter tjernbergiae-13	2	Acidovorax-13	2	Acinetobacter tjernbergiae-13	2
Acinetobacter junii-11	2			Acinetobacter tjernbergiae-12	2
Achromobacter-13	2				

Table S5: Classification results without summing lower occurring objects over abundance bin after object merging.

Green	
Comamonas_jiangduensis-hi	14
Pseudomonas-hi	10
Cloacibacterium-hi	7
Acinetobacter junii-14	5
Acinetobacter junii-13	5
Moraxella-hi	3
Acinetobacter junii-12	2

Orange	
Acidovorax-hi	17
Acinetobacter tjernbergiae-13	13
LoCnt-hi	12
Acinetobacter junii-13	12
Acinetobacter junii-14	11
Cloacibacterium-hi	9
Acinetobacter tjernbergiae-14	9
Cutibacterium acnes-13	7
Cutibacterium acnes-12	3
Sediminibacterium-hi	2
Pseudomonas-hi	2
Cutibacterium acnes-14	2
Delftia-hi	2
Acinetobacter tjernbergiae-12	2

Blue	
Cutibacterium acnes-14	26
Moraxella-hi	7
Streptococcus-hi	4
LoCnt-hi	3
Kocuria-hi	3
Acinetobacter tjernbergiae-12	3
Acinetobacter junii-13	3
Acinetobacter junii-12	3
Achromobacter-hi	3
Sediminibacterium-hi	2
Nitrosospira-hi	2
Acinetobacter tjernbergiae-13	2

Red	
Cutibacterium acnes-14	14
Nitrosospira-hi	10
Sediminibacterium-hi	8
Cutibacterium acnes-13	6
Bradyrhizobium-hi	6
LoCnt-hi	5
Meiothermus-hi	4
Kocuria-hi	4
Stenotrophomonas-hi	3
Cloacibacterium-hi	3
Cutibacterium acnes-12	2
Acinetobacter tjernbergiae-12	2
Acidovorax-hi	2

Magenta	
Cutibacterium acnes-13	9
LoCnt-hi	9
Cutibacterium acnes-12	7
Methylobacterium-hi	7
LoCnt-14	6
Delftia-hi	6
Noviherbaspirillum-hi	5
Streptococcus-hi	4
Bacillus-hi	4
Sediminibacterium-hi	3
Acinetobacter junii-12	3
Acinetobacter tjernbergiae-13	2
Acinetobacter tjernbergiae-12	2
Acidovorax-hi	2
Achromobacter-hi	2

Table S6: Main Microbial Objects By Class and Disease State - Individual Samples

Green			
**Cutibacterium**	**Acinetobacter**	**Comamonas**	**Disease**
C. acnes-13	A. junii-13	none	AD
C. acnes-14	none	none	C
C. acnes-14	A. junii-13	none	AD
C. acnes-13	A. junii-13	none	AD
C. acnes-11	A. junii-13	none	AD
C. acnes-9	A. junii-13	none	AD
C. acnes-13	A. junii-14	none	C
C. acnes-10	A. junii-13	C. jiangduensis-7	AD
C. acnes-9	A. junii-14	C. jiangduensis-11	C
C. acnes-7	A. junii-14	C. jiangduensis-10	C
C. acnes-12	A. junii-13	none	AD
C. acnes-13	A. junii-13	none	AD
C. acnes-10	A. junii-13	C. jiangduensis-9	AD
C. acnes-8	A. junii-13	none	AD
C. acnes-10	A. junii-14	C. jiangduensis-11	C
none	A. junii-14	C. jiangduensis-11	C
none	A. junii-14	none	AD
C. acnes-12	A. junii-13	none	AD
none	A. junii-13	none	AD
C. acnes-13	A. junii-14	none	AD
C. acnes-13	A. junii-14	none	C
C. acnes-11	A. junii-14	none	AD
C. acnes-12	none	C. jiangduensis-7	C
C. acnes-9	A. junii-14	C. jiangduensis-10	C
C. acnes-7	A. junii-14	none	C
C. acnes-13	none	none	C
none	A. junii-11	none	AD

Orange			
**Cutibacterium**	**Acinetobacter**	**Comamonas**	**Disease**
none	none	C. jiangduensis-14	C
none	A. junii-13	C. jiangduensis-12	C
none	A. junii-12	C. jiangduensis-14	C
none	A. junii-14	C. jiangduensis-13	C
none	A. junii-13	C. jiangduensis-14	C
C. acnes-10	A. junii-13	C. jiangduensis-14	C
C. acnes-14	A. junii-14	C. jiangduensis-13	C
C. acnes-11	A. junii-13	C. jiangduensis-14	C
C. acnes-7	A. junii-14	C. jiangduensis-13	C
C. acnes-8	A. junii-14	C. jiangduensis-13	C
C. acnes-11	A. junii-12	none	C
none	A. junii-10	C. jiangduensis-14	C
C. acnes-11	A. junii-13	C. jiangduensis-14	C
C. acnes-13	A. junii-14	C. jiangduensis-11	C
none	none	C. jiangduensis-13	C
none	none	C. jiangduensis-12	C

Blue			
**Cutibacterium**	**Acinetobacter**	**Comamonas**	**Disease**
C. acnes-14	none	none	C
C. acnes-14	A. junii-11	none	AD
C. acnes-14	none	none	AD
C. acnes-14	none	none	C
C. acnes-14	none	none	C
C. acnes-14	A. junii-10	none	C
C. acnes-14	A. junii-9	none	C
C. acnes-14	A. junii-9	none	C
C. acnes-14	A. junii-12	none	C
C. acnes-14	A. junii-10	none	C
C. acnes-14	A. junii-13	none	C
C. acnes-14	none	none	AD
C. acnes-14	A. junii-10	none	C
C. acnes-14	none	none	AD
C. acnes-14	A. junii-13	none	C
C. acnes-14	A. junii-12	none	AD
C. acnes-14	A. junii-13	none	C
C. acnes-14	none	none	AD
none	none	none	AD
C. acnes-14	A. junii-10	none	AD
C. acnes-14	none	none	C
none	A. junii-10	none	C
C. acnes-14	A. junii-11	none	AD
C. acnes-14	A. junii-12	none	C
C. acnes-12	A. junii-9	none	C
C. acnes-14	none	none	C
C. acnes-14	none	none	C
C. acnes-14	none	none	C
C. acnes-14	none	none	AD

Red			
**Cutibacterium**	**Acinetobacter**	**Comamonas**	**Disease**
C. acnes-14	none	none	AD
C. acnes-14	none	none	AD
C. acnes-14	none	none	C
C. acnes-13	none	none	AD
C. acnes-11	none	none	AD
C. acnes-12	none	none	C
C. acnes-14	A. junii-13	none	AD
C. acnes-13	none	none	AD
none	none	none	C
C. acnes-13	none	none	AD
C. acnes-14	none	none	C
C. acnes-12	none	none	AD
none	none	none	C
C. acnes-14	none	none	C
C. acnes-14	none	none	AD
C. acnes-14	A. junii-11	none	C
C. acnes-14	none	none	AD
C. acnes-13	A. junii-11	none	AD
C. acnes-14	none	none	AD
C. acnes-14	none	none	AD
C. acnes-14	none	none	AD
C. acnes-14	A. junii-10	none	AD
C. acnes-13	none	none	C
none	none	none	C
C. acnes-13	none	none	AD
C. acnes-14	none	none	AD

Table S6: (cont.)

Magenta			
**Cutibacterium**	**Acinetobacter**	**Comamonas**	**Disease**
C. acnes-13	A. junii-12	none	AD
none	none	none	AD
none	none	none	AD
C. acnes-13	none	none	C
C. acnes-12	none	none	AD
C. acnes-12	none	none	AD
C. acnes-12	A. junii-14	none	AD
C. acnes-12	A. junii-13	none	AD
C. acnes-13	none	none	AD
C. acnes-13	none	none	AD
C. acnes-13	none	none	AD
none	none	none	AD
C. acnes-12	none	none	AD
C. acnes-11	none	none	AD
C. acnes-13	none	none	AD
C. acnes-10	A. junii-12	none	C
C. acnes-13	none	none	AD
C. acnes-13	none	none	AD
C. acnes-13	none	none	AD
C. acnes-11	none	none	AD
none	none	none	AD
C. acnes-12	none	none	C
C. acnes-12	A. junii-12	none	AD
C. acnes-11	none	none	AD

Table S7: Microbiome objects approximated from sample input data of a given color - in counts.

OBJECT	(green)	(orange)	(blue)	(red)	(pink)
Cutibacterium_acnes-14	1	2	26	14	0
Cutibacterium_acnes-13	1	7	0	6	9
Acinetobacter_junii-13	5	12	3	1	1
Acinetobacter_tjernbergiae-13	0	13	2	0	2
Acinetobacter_junii-14	5	11	0	0	1
Cloacibacterium-12	5	8	0	0	0
Acidovorax-13	0	9	1	2	0
Cutibacterium_acnes-12	0	3	0	0	7
Acinetobacter_tjernbergiae-14	0	9	0	0	0
Comamonas_jiangduensis-14	7	0	0	0	0
Sediminibacterium-13	1	1	0	2	2
Acinetobacter_junii-12	0	0	3	0	3
Sediminibacterium-12	0	0	0	5	0
Pseudomonas-12	5	0	0	0	0
Nitrosospira-14	0	0	1	3	1
Nitrosospira-13	0	0	0	5	0
Delftia-14	1	0	0	0	4
Comamonas_jiangduensis-13	5	0	0	0	0
Acidovorax-14	0	4	0	0	1
Streptococcus-12	0	0	4	0	0
Pseudomonas-13	4	0	0	0	0
Moraxella-12	0	0	4	0	0
Cloacibacterium-13	1	1	0	2	0
Acidovorax-12	0	4	0	0	0
Achromobacter-13	0	1	2	0	1
Noviherbaspirillum-12	0	0	0	0	3
Moraxella-13	0	0	3	0	0
Methylobacterium-13	0	0	0	0	3
Corynebacterium-13	0	0	2	0	1
Bradyrhizobium-13	0	0	0	3	0
Acinetobacter_tjernbergiae-12	0	0	3	0	0
Variovorax-14	0	0	0	0	2
Variovorax-13	0	0	0	0	2
Stenotrophomonas-13	0	0	0	2	0
Sphingomonas-14	0	1	0	0	1
Sediminibacterium-14	0	0	0	1	1
Noviherbaspirillum-13	0	0	0	0	2
Moraxella-14	2	0	0	0	0
Methylobacterium-14	0	0	0	0	2
Meiothermus-13	0	0	0	2	0
Kocuria-14	0	0	0	2	0
Kocuria-13	0	0	1	1	0
Janthinobacterium-14	0	0	1	0	1
Caulobacter-14	0	0	0	0	2
Bradyrhizobium-14	0	0	0	2	0
Bacteroides-13	2	0	0	0	0
Bacillus-14	0	0	0	0	2
Actinomyces-13	0	0	0	0	2
Achromobacter-14	0	0	1	1	0
Virgibacillus-14	0	0	0	0	1
Veillonella-13	0	0	0	0	1
Tabrizicola-13	0	1	0	0	0
Streptococcus-14	0	0	0	0	1
Streptococcus-13	0	0	0	0	1
Stenotrophomonas-14	0	0	0	0	1
Sporocytophaga-13	0	0	0	0	1
Solirubrobacter-13	0	0	1	0	0
Snodgrassella-13	0	0	0	0	1
Singulisphaera-13	0	1	0	0	0
Shigella-14	0	0	1	0	0
Rubellimicrobium-14	0	0	0	0	1
Roseomonas-14	0	1	0	0	0
Ralstonia-13	0	0	0	0	1
Pseudomonas-14	1	0	0	0	0
Pedobacter-13	0	0	0	0	1
Paracoccus-13	0	0	0	0	1
Paraburkholderia-13	0	1	0	0	0
Novosphingobium-14	0	1	0	0	0
Nocardioides-13	0	0	0	0	1
Niabella-13	0	0	0	0	1

Table S7: (cont.)

OBJECT					
Neomicrococcus-13	0	0	0	0	1
Microbacterium-13	0	0	1	0	0
Massilia-14	0	0	0	0	1
Lysinibacillus-13	0	0	0	1	0
Lactobacillus-13	0	0	0	1	0
Lachnoclostridium-13	1	0	0	0	0
Klebsiella-13	0	0	0	0	1
Jeotgalicoccus-14	0	0	1	0	0
Janthinobacterium-13	0	0	1	0	0
Herbiconiux-13	0	0	0	0	1
Gemella-14	0	0	1	0	0
Flavihumibacter-13	0	1	0	0	0
Ferrovibrio-14	0	0	0	1	0
Enterococcus-13	0	0	0	1	0
Empedobacter-13	0	0	0	1	0
Delftia-13	0	0	0	0	1
Cutibacterium-13	0	0	1	0	0
Comamonas_testosteroni-13	0	1	0	0	0
Clostridium-14	0	0	1	0	0
Clostridioides-13	0	0	1	0	0
Cloacibacterium-14	1	0	0	0	0
Cellulosimicrobium-13	0	0	0	0	1
Brevundimonas-14	0	0	0	0	1
Brachybacterium-13	0	0	1	0	0
Bosea-14	0	0	0	1	0
Bosea-13	0	0	0	0	1
Blautia-13	0	1	0	0	0
Blastocatella-14	0	0	0	1	0
Bacteroides-14	1	0	0	0	0
Aquisphaera-13	0	0	0	0	1
Anaerococcus-13	0	0	0	0	1
Anabaena-13	0	1	0	0	0
Actinoplanes-13	0	0	1	0	0
Acinetobacter_guillouiae-13	0	0	0	0	1
Acinetobacter_tjernbergiae-10	6	0	4	0	0
Cloacibacterium-11	4	4	0	0	0
Comamonas_testosteroni-10	0	7	0	0	0
Cloacibacterium-10	0	7	0	0	0
Acidovorax-11	0	7	0	0	0
Streptococcus-11	3	0	0	0	3
Cutibacterium_acnes-11	3	0	0	0	3
Comamonas_testosteroni-11	0	6	0	0	0
Acidovorax-8	3	0	0	0	3
Novosphingobium-9	0	5	0	0	0
Corynebacterium-11	0	0	0	0	5
Acinetobacter_junii-10	0	0	5	0	0
Zoogloea-9	0	4	0	0	0
Pseudomonas-8	0	4	0	0	0
Moraxella-11	4	0	0	0	0
Lactobacillus-10	4	0	0	0	0
Delftia-11	0	0	0	4	0
Corynebacterium-10	0	0	4	0	0
Sphingobium-10	0	3	0	0	0
Sediminibacterium-11	0	0	3	0	0
Pseudomonas-9	0	3	0	0	0
Cutibacterium_acnes-9	0	3	0	0	0
Cutibacterium_acnes-10	0	3	0	0	0
Moraxella-10	0	0	0	3	0
Kocuria-10	0	0	3	0	0
Comamonas_testosteroni-9	0	3	0	0	0
Comamonas_jiangduensis-11	0	3	0	0	0
Bradyrhizobium-10	0	0	3	0	0
Bacillus-10	0	3	0	0	0
Anaerococcus-8	3	0	0	0	0
Acinetobacter_tjernbergiae-11	3	0	0	0	0
Acinetobacter_junii-9	0	0	3	0	0
Acinetobacter_baumannii-9	3	0	0	0	0
Acinetobacter_baumannii-8	3	0	0	0	0
Acidovorax-9	0	0	3	0	0
Achromobacter-9	0	3	0	0	0
Achromobacter-10	0	0	3	0	0

Figure S1: Differences in relative abundance between the Alzheimer's disease (AD) group and the age-matched control group (controls). The relative abundances were estimated for each OTU from each group through hierarchical Bayesian modeling while ignoring the non-independence of the samples. The vertical axis shows the difference for the estimated relative abundance of OTU between the AD and control groups. Points are the means of PPD and the whiskers show the 95% equal tail probability intervals of PPD (see Materials and methods).

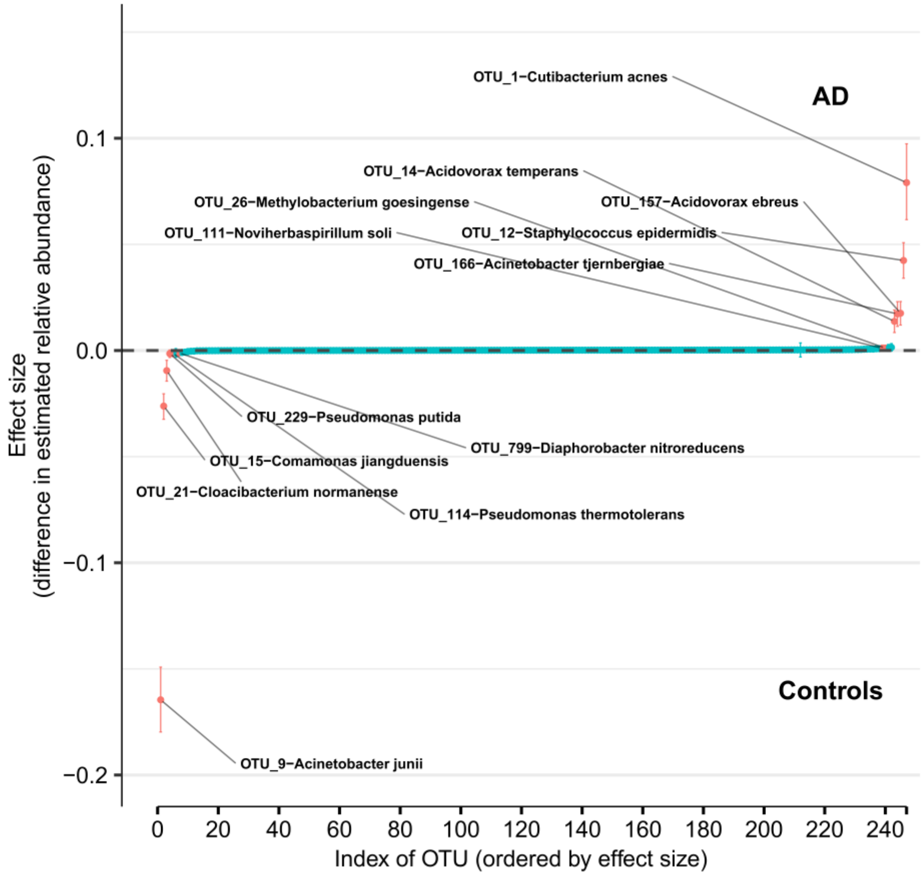

Supplement: Supplementary Table S1 — OTU identified as potential contaminants. [file DataSheet_1.zip › Supplementary_Material_ALL_Tables_Figure.pdf]
