# Supplementary material for: Evidence supportive of a bacterial component in the etiology for Alzheimer’s disease and for a temporal-spatial development of a pathogenic microbiome in the brain
Source: Front Cell Infect Microbiol. 2023 Sep 13;13:1123228. doi: 10.3389/fcimb.2023.1123228 (PMC10534976; doi:10.3389/fcimb.2023.1123228)
Supplement: Supplementary Table S1 — OTU identified as potential contaminants. [file DataSheet_1.zip › Supplementary_Methods.pdf]

SUPPLEMENTARY METHODS

This supplement contains details of the computational methods that we used in this work. Our goal is to provide a heuristic approach to these methods rather than go overboard with mathematical rigor.

Dirichlet-Multinomial modelling (DMM). We summarize the DMM algorithm which is a Bayesian approach to comparing compositional data.

Latent Dirichlet Allocation (LDA). We begin with a summary of the overall algorithm. We then proceed with a semi-formal derivation of the LDA classifier that differs from the derivations in the literature. The classifier is used to assign classes to the input objects from the data set. It is essentially an unfair die that is repeatedly rolled until the classification converges. We then go on to describe modifications to the algorithm that we implemented to improve convergence and repeatability.

Color Classes. In our analysis, we make heavy use of color for visualization of class. We describe what we mean by color classes as opposed to class.

Parameter Setting and Input Object Optimization. There are several adjustable parameters in the computation that include LDA hyper-parameters and the number classes with which to classify. One can also consider the binning structure and microbe grouping as an adjustable parameter too. We explain in detail what we did and specifically how we used convergence tests to assure that our choices were reasonable. We also show how we visualized the performance and convergence of the interim LDA results with graph theoretics.

Graph Visualization. We provide an expanded presentation of the graph section of the paper that includes issues such as similarity, embedding and convergence.

1. Differential abundance analysis using the Dirichlet-Multinomial model (DMM)

To investigate the differences in relative abundances for each OTU between AD and control conditions, we used the Dirichlet-Multinomial modelling approach described in Harrison et al. (Harrison et al., 2020). Here we describe the specification of the DMM. The model was implemented using the Stan probabilistic programming language through the Python interface Pystan (version 2.19.1.1), the code is available at:

https://github.com/jlapides/alzheimers_method_1/blob/main/Additional file 2.rmd

DMM estimates the relative abundances (*i.e.* proportions) of OTUs within a sample as parameters of a multinomial distribution which are a vector of probabilities (vector $\vec{p}$). Multinomial parameters are modeled using a Dirichlet distribution as prior. The parameters of the Dirichlet distribution describe the expected relative abundances of each OTU for the entire sampling group (vector $\vec{\pi}$).

To take into account that some samples were from the same individual subject (non-independent samples), the vector $\vec{\pi}$ is informed by an additional Dirichlet distribution with $\vec{\psi}$ that describes the relative abundance of OTUs within each subject and the intensity parameter τ.

The model was specified as follows:

$$\vec{x}_i Multinomial\left(\vec{p}_i, N_i\right) \quad (1)$$

$$\vec{p}_i Dirichlet\left(\vec{\pi}_k, \theta_k\right) \quad (2)$$

$$\vec{\pi}_k Dirichlet\left(\vec{\psi}_s, \tau_s\right) \quad (3)$$

$$\vec{\psi}_s Dirichlet\left(\vec{\alpha}\right) \quad (4)$$

with priors,

$$\theta_k Exponential(\lambda = 0.01) \quad (5)$$

$$\tau_s Exponential(\lambda = 0.01) \quad (6)$$

$\vec{x}_i$ is the observed count of a particular OTU in the sample i. $\vec{p}_i$ is the vector of the estimated OTU proportion and N_i is the total counts in each sample i. $\vec{\pi}_k$ is the estimated proportion of each OTU in the sampling group k and θ_k is the intensity parameter of the Dirichlet distribution. $\vec{\psi}_s$ describes the expected relative abundance of OTU within each subject s and τ_s is the intensity parameter of the Dirichlet distribution. The prior for the $\vec{\psi}_s$ parameter is a Dirichlet distribution with equal prior probability for each OTU. Exponential distributions have been used as the prior for θ_k and τ_s.

To quantify the differences in relative abundance between AD and control groups, the posterior probability distribution (PPD) for the OTU of interest in the control group was subtracted from the PPD of that OTU in the AD group. Following convention, if 95% of the PPD for difference does not overlap zero, there is high certainty that the OTU of interest differs in relative abundance between AD and control.

DMM was specified in the Stan probabilistic programming language through the Python interface Pystan (version 2.19.1.1) that implements the Hamiltonian Monte Carlo No-U-Turn sampler (HMC-NUTS) algorithm. For each of four chains, 3500 iterations were used with 1500 burn-in and a total of 4000 samples were drawn (thin=2). Convergence was assessed using the Gelman-Rubin statistic (Gelman and Rubin, 1992).

Latent Dirichlet Allocation Algorithm Summary.
In essence the LDA algorithm consists of computing the single-valued class of each measurement object in a data set. LDA assumes that the class of an object is determined first by the chance that each class is present in the sample and second, for each class, what the chances are of measuring a particular object. In other words, the class of an object is determined by two probability distributions. The former distribution is given by the sample class distribution and the

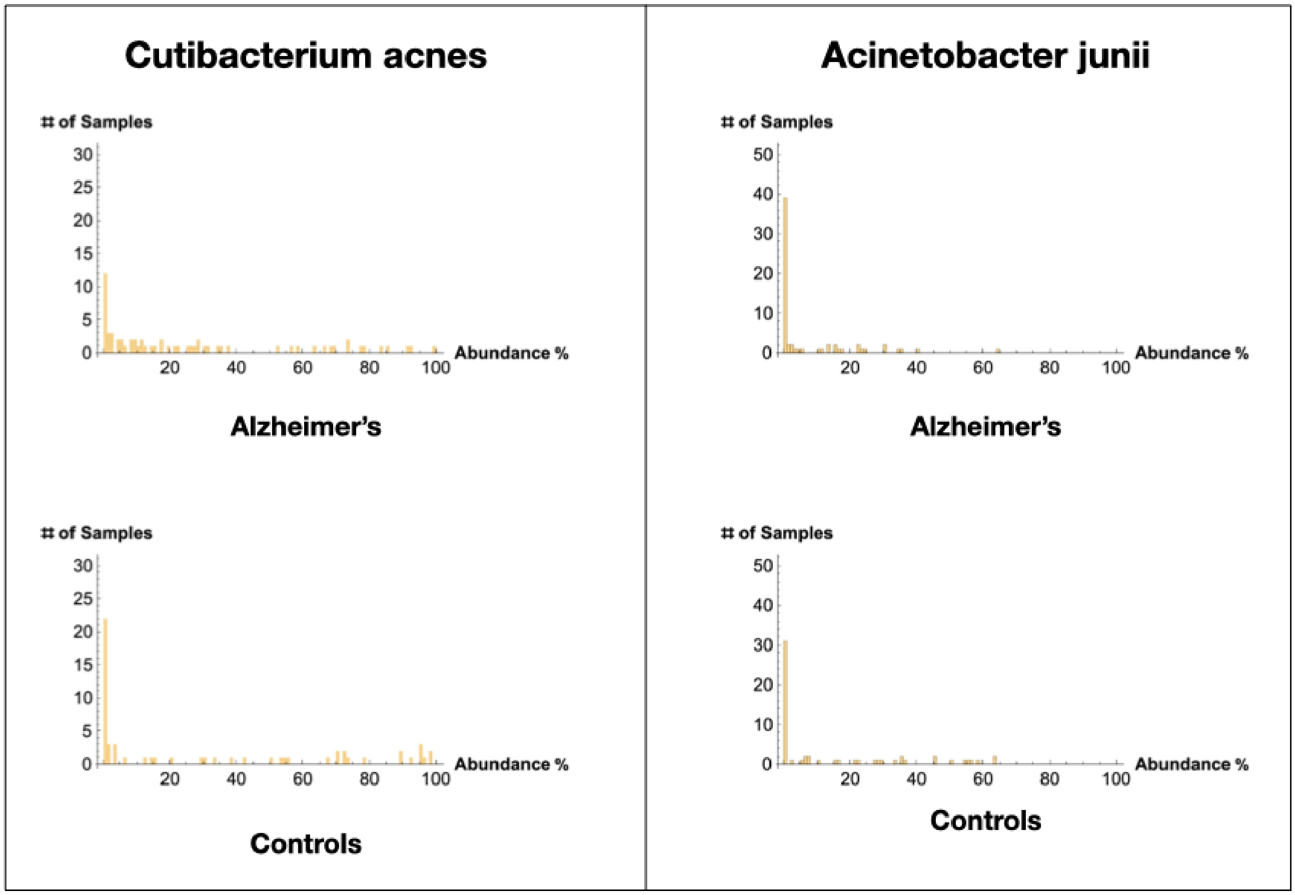

Figure SF1: Comparisons of AD & control abundance distributions for *Cutibacterium acnes* and *Acinetobacter junii*.

latter by the object class distribution. The probability of the class of the object is roughly the component-by-component product of both distributions. Unfortunately, we do not know these distributions a priori. In other words, this is one of those situations where we need to know the answer in order to compute it.

One way to solve this problem is through iteration. We chose a Markov chain Monte Carlo (stochastic) approach (Griffiths and Steyvers, 2004) although there are others. In general, since we do not know the distributions, we begin the assignment computation by randomly assigning classes to all of the microbial objects in all of the samples using a die with equal weights. This allows starting sample and object distributions to be calculated with their normalized values interpreted as probabilities for the stochastic computation. Then we cycle through the entire data set over and over (iterations) recomputing the assignments. For each object, we recompute the two probability distributions, multiply them component by component and renormalize. The components form the weights of an unfair die which is used as a random number generator whose probabilities contain complete information of the object and sample. When the die is rolled, it yields a class which is used to update the class assignment of the object. This sequence of stochastic computations forms a Markov chain since each depends on the last.

Since the die weights are functions of the distributions which are obtained from the assignments, every roll of the die changes the die weights a small amount. Eventually, as the iterations proceed, the distributions stop changing and converge, hopefully resulting in a stable and repeatable class distribution for each sample class distribution (SD) and for each object class distribution (OD). It is a bit more complicated than this and we describe other features that are necessary for convergence below. Generally, we used 1000 iterations.

There is one other way to look at what we are doing that helps to distinguish this method from other dimensionality reduction approaches. We note that one of the distributions, the sample distribution, is computed only from the sample while the other, the object distribution, is computed from all samples. We can call the sample distribution a local distribution because it only uses class assignments from the sample and the object distribution a global distribution because it uses class assignments from all the samples. Using this terminology, we see that the class of each object is determined by both a local and global contribution. As long

as the experiment was adequately designed so that local and global information is adequately sampled, the scheme can work. Other dimensionality reduction schemes, e.g. t-SNE and UMAP (van der Maaten and Hinton, 2008; McInnes et al., 2018) tend to focus on sample attributes, e.g. making sure that sample closeness in the n-dimensional input space is preserved in the lower dimensional output space. Local schemes like these may have trouble allowing objects (or measurements) to have multiple meanings although the common objects within the samples of a sample cluster in low dimensions could represent common meaning the way a class does in LDA. The rigorous definition of cluster may involve a second computational step or often, eyeballing. In contrast, the LDA cluster is intrinsically defined by class. PCA, on the other hand, does have a global contribution even though its results are linear combinations of sample abundances. The global dependence enters through the coefficients that define the combination which will depend on the abundance variances of individual microbes across samples. A full exploration of the relationships among these methods is beyond the scope of this paper.

Color Classes. In order to make analysis and discussion easier, we label the components of the classifications with colors. From this labeling, we define the color of a sample class distribution or object distribution to be the color of its largest component. For example, a red sample's largest component is the red component. We also use the concept of color to approximate or describe microbiomes. In this example, it can be thought of as the set of microbial objects that occur in samples of a given color.

LDA Classifier Derivation. Following is a heuristic derivation of the Latent Dirichlet Allocation (LDA) classifier we described above that provides a more intuitive sense of the algorithm without resorting to Bayesian probability theory. Rigorous Bayesian derivations can be found here (Geman and Geman, 1984; Hofmann, 2001; Blei et al., 2003; Griffiths and Steyvers, 2004; Heinrich, 2008, 2009; Blei, 2012).

Specifically, we will describe how to compute the probability weights of the die which are a function of the sample distribution (SD) and the object distribution (OD). We begin by assuming that the classifier formula will depend in some way on the two distributions. Our approach is to find the simplest reasonable combination of the distributions. In this way, we arrive at a result that has an easy intuitive appeal.

The simplest expression that one can imagine for the weights involves the sum of four terms, two linear terms, a quadratic term and a constant. Each of the terms is a vector with length C, the number of classes with which we will classify. Keep in mind that the die roll assigns the class for one microbial object. All of the vector multiplications below are element-wise products where vector components from each vector are multiplied. In other words, the product of two 5-component vectors yields a 5-component vector.

They are as follows:

Term 1: It seems reasonable to suppose that the class of a particular object in a sample should be proportional to the class count distribution (SD) for the entire sample. The proportionality constant is a constant vector (vector of equal constants).

$$\beta \cdot SD \tag{7}$$

where SD means sample distribution.

Term 2: Since the object may occur in many samples, there should also be a term that reflects its classification in all of the samples which is the class distribution of the particular object (OD) being classified. The proportionality constant is a constant vector here as well.

$$\alpha \cdot OD \tag{8}$$

where OD means object distribution.

Term 3: There should also be a term that is the component-by-component product of these two distributions. The meaning of this is seen to be the joint distribution of the two.

$$SD \cdot OD \tag{9}$$

where for now we assume no proportionality constant.

Term 4: A constant, K.

Combining the four terms we obtain:

$$classifier \propto OD \cdot SD + \alpha \cdot OD + \beta \cdot SD + K \tag{10}$$

We can express equation (10) as the product of two terms.

$$= (OD + \beta)(SD + \alpha) \tag{11}$$

where the constant is seen to be:

$$K = \alpha \cdot \beta \tag{12}$$

This way of expressing the relationship suggests the form of the denominator, D. For a frequency, we need an expression that is unit-less. Since the numerator is in units of counts, the denominator needs to be in counts as well. A reasonable linear form is have the object count term be divided by the total number of all objects assigned to each class, $\sum_{all} O$, plus a constant and the sample term be divided by the number of objects in the sample, NS, plus a constant. (Neither should include the object being classified which is required by probability theory and is also the case for the above distributions).

$$D = (\textstyle\sum_{all} O + A1) \cdot (NS + A2) \tag{13}$$

$$D = \textstyle\sum_{all} O \cdot (NS + A2) + A1 \cdot NS + A1 \cdot A2 \tag{14}$$

In practice, the first two of these terms dominate with the count product being the largest. We have found that the calculation converges with the Alzheimer's data sets using the sum over objects term alone and normalizing. So in summary, the classifier is expressed in the following way:

$$classifier = \left(\frac{1}{D}\right) \cdot (OD + \beta)(SD + \alpha) \tag{15}$$

We can compare this result to equation (5) of (Griffiths and Steyvers, 2004) which is a rigorous result and see that it is the same result.

$$P(z_i = j|\mathbf{z}_{-i}, \mathbf{w}) \propto \frac{n_{-i,j}^{(w_i)}+\beta}{n_{-i,j}^{(\cdot)}+W\beta}\frac{n_{-i,j}^{(d_i)}+\alpha}{n_{-i,\cdot}^{(\cdot)}+T\alpha} \quad (16)$$

where P is the classifier and the $n's$ correspond to the distributions in the above formulas with

$$A1 = W \cdot \beta = NO \cdot \beta \quad (17)$$

and

$$A2 = T \cdot \alpha = C \quad (18)$$

W is the number of unique objects in a data set which we call NO, and T is the number of classes in the computation which we call C in this paper.

The dot indicates that the distributions are to be calculated without using the object that is being classified. This is the case you obtain when the distributions are estimates of conditional probabilities which is technically what they are. The final expression is then:

$$classifier = \frac{(OD+\beta)}{(\sum_{all} O+NO\cdot\beta)}\frac{(SD+\alpha)}{(NS+C\cdot\beta)} \quad (19)$$

The components of the classifier, a vector with dimension equal to the number classes one is trying to classify into, are the weights of the die that we described above. This is the stochastic formula we use to classify an individual object. So, we will be classifying an individual object using the class distribution of the sample it is in and the object distribution of all the other occurrences of the object in the data set. We used the estimation rules for α and β presented in Griffiths and Steyvers (Griffiths and Steyvers, 2004) but also explored their parameter spaces. They suggest that α be 50/C. We adjusted to a lower value for α of 0.5 and retained the value of β suggested, 0.1, to obtain good graphical clusters.

Modified LDA (MLDA). Popular implementations of LDA algorithms use the sample and object distributions obtained after completing a number of iterations sufficient for these distributions to converge. For our data set, and in many others too, this approach does not result in repeatable distribution computations. We added the following procedures to improve repeatability.

<u>Accumulation, Thinning and Burn-in</u>. Rather than using the terminal distributions as the result, we accumulated the count distributions over the course of the iterations. To guard against correlations between iterations, we included results from one iteration after n iterations in the accumulation, typically 5. Sometimes this is called thinning. These two procedures were suggested by Heinrich (Heinrich, 2008, 2009).

When the classification begins, the distributions are far from convergence and would distort the accumulation if included, so we begin the accumulation after some number iterations, typically 100, determined after convergence monitoring (see below).

<u>Randomization</u>. The order of sample classification was randomized with every iteration. We did not randomize the object classification order within sample although it may be useful.

<u>Classifier Mixing</u>. After the burn-in we add additional averaging to the classifier beginning at 150 iterations which is performed for every object classification. This is done by computing the object and sample count distributions and then averaging in a small amount of the accumulated distribution, typically 4%, scaled by the ratio of counts in the current distribution to the accumulated distribution. The averaging is delayed until 150 iterations to allow for some accumulation to occur after burn-in.

<u>Classifier Filtering</u>. We filtered the classifier to include only the top three components, turning the classifier into a three-sided die.

<u>Run Summing</u>. The last layer of averaging added together multiple runs, usually five. We found that we could not just extend the length of a run to improve the convergence. After long runs, the classifications can destabilize and increase their entropy. We discovered that we could obtain better repeatability by summing multiple runs.

<u>Mapping Classes before Run Summing</u>. Since the computation begins with a random assignment of classes to the objects, the class labels (1, 2, 3, …) or their equivalent colors, change from run to run, requiring that they be mapped to a common labeling scheme before summing. We compared the samples by color class with assistance from the graphs to accomplish this.

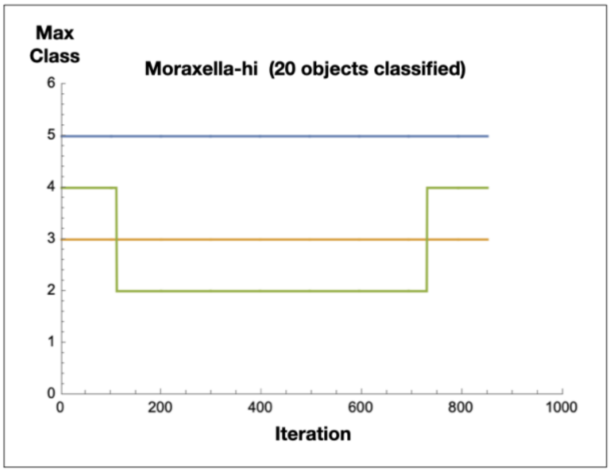

Figure SF2: Convergence Monitoring (Fine).

If the computation and graph of individual runs are fairly repeatable, we should end up with the same clusters within statistical fluctuation but having different colors (labels). If our computations are not repeatable, we will see splitting of classes where, for example, the samples in cluster from one run end up with two or more other different clusters. On the other hand, if most of the samples from a particular class in one run correspond to most of the samples in another class

in another run, we can see corresponding topologies and confirm it by checking which samples are in each cluster. In practice, run summing stabilizes the minority of samples that jump between color classes.

Convergence Monitoring. We monitor convergence in both a coarse and fine way. The coarse method is to compute the average entropy of the sample distributions and the object distributions. We normalize the entropy of the standard entropy by dividing by the entropy of a constant distribution for a vector of length C. This has the effect of making the entropy vary from zero to one.

$$Entropy = \sum normalizeddistribution \cdot log(normalizeddistribution) \quad (20)$$

A = entropy of a vector of length C with each component equaling 1/C. (21)

$$NormalizedEntropy = \frac{Entropy}{A} \quad (22)$$

The entropy of the sample and object distributions tends to decline rapidly to values around 0.5 or below. Stable classifications will have lower entropy but low entropy does not always mean a stable classification so we also do the following.

For fine monitoring, we grab the maximum component label for each sample and each object. We make sure that the maximum components remain the same for 30-50% of the last iterations. See Figure SF2. Iterations are plotted beginning after burn-in. If we look up the final component values of this object, we find that blue is 0.8, orange is 0.06, and green is 0.12. The graph shows that the main component, blue, is stable from after burn-in until the end. If there were hopping at the left end, we would have increased the burn-in. The two others are smaller components and so we expect some instability. In this way we checked the stability of all samples and objects. Generally, we strove to have the main component be very stable, giving us confidence that there were no issues with burn-in. In setting burn-in and other iteration parameters, we also made sure to be well beyond where entropy stopped declining.

Repeatability. For the graph to be a useful representation of reality, it must be reasonably repeatable. These are stochastic computations, so we do expect some variability between runs. Operationally, we defined repeatability in the following way. We performed multiple runs then added each sample's count distributions together. We assessed the repeatability by comparing the populations of each color cluster of the summed graph with the populations of the color clusters of the individual runs that comprised it. The populations of the summed graph matched those from individual runs in the 80-90% range for each run in each color cluster.

Parameter Setting and Optimization. It will become clear that there are many adjustable parameters in the analytical methodology we used for combinations of bacteria. These include LDA input parameters (e.g. number of classes), Monte Carlo parameters (e.g. number of iterations), parameters that describe the LDA results in graph visualizations (e.g. spring constant), data abundance binning and phylogenetic level summation(naming). This constitutes a large parameter trade space for which we do not have a rigorous optimization methodology. There are several criteria we used to guide our choice of parameters. The last set is treated in more detail in the following section.

1) Convergence of the sample and object class distributions. After some number of iterations in the LDA Monte Carlo computation, we require individual sample class distributions and object distributions to change little after a preset number of iterations. See Figure SF2 and Convergence Monitoring above.

2) Repeatability. We require that the same samples end up with the same color classes defined above after repeated runs. This is critical because it is possible that there could be convergence without repeatability. A rigorous repeatability evaluation method is detailed below.

3) Sample and Object Graph Quality. The graphs, described above, are a holistic comparison of every pair of samples or objects. The abiding feature of the graphs is that similarly colored nodes cluster together which randomly generated data does not do. In unpublished results of about 7,000 subject's gut microbiomes, we saw this pattern ubiquitously. Without proof we used this feature to adjust parameters to get the tightest well-defined homogeneously colored clusters. It seems reasonable to suppose that tight, homogeneously colored graphs represent repeatable computations because the opposite will have ill-defined (multi-colored) and small features that are prone to statistical fluctuations and thus lack repeatability.

Even with non-optimized parameters, LDA can provide insights about the data, so we explored the parameter space by running the LDA algorithm hundreds of times both to optimize the parameters and get a sense for patterns in the data. We focused on whether objects and samples had repeatable class component maxima (colors) or whether component values were roughly equal. If class means something, the former occurs while if the classification is independent of class, the latter occurs. This is particularly important in the binning and naming adjustments of the next section.

It is important to realize that we are using these techniques to find qualitative patterns in the data leading to insights and the formation of new hypotheses about the biology of AD. Consequently, rigorous optimization and justification of particular parameter values was not attempted, relying instead on a trial-and-error approach and the general criteria above.

Overall, once the criteria were met, we did not continue optimizing parameters, but froze them and tried to discern if the sample and object classification patterns revealed underlying biology. Further validation of this method is important, but we emphasize that there is already an extensive literature on LDA's ability to find classes (topics in the literature) in documents by finding words that co-

occur within documents. Of course, LDA can't tell the difference between a microbial object and a word.

Abundance Binning, Microbe Naming and Object Merging. To utilize the LDA algorithm, the data needed to be converted to objects. This required a phylogenetic level (naming) to sum the OTU data to and an abundance binning structure. The raw data consists of OTU counts labeled by subject, sample, and bacterium. We removed measured contaminants which is described in a subsequent section. The *Cutibacterium* and *Acinetobacter* genera occurred the

MICROBIAL OBJECT	Alzheimer's	Controls	MICROBIAL OBJECT	Alzheimer's	Controls
Cutibacterium acnes-14	20	23	Acidovorax-8	4	3
Cutibacterium acnes-13	17	6	Acinetobacter junii-10	2	5
Acinetobacter junii-13	14	8	Comamonas jiangduensis-14	0	7
Acinetobacter tjernbergiae-13	11	6	Sediminibacterium-13	4	2
Acinetobacter junii-14	4	13	Pseudomonas-9	4	2
Cloacibacterium-12	9	6	Comamonas testosteroni-11	4	2
Cutibacterium acnes-12	9	4	Sediminibacterium-11	3	3
Acidovorax-13	8	4	Streptococcus-12	2	4
Acinetobacter tjernbergiae-10	1	10	Nitrosospira-13	4	1
Cloacibacterium-11	3	7	Moraxella-10	4	1
Acidovorax-11	3	7	Delftia-14	4	1
Cutibacterium acnes-11	6	3	Acinetobacter junii-11	4	1
Acinetobacter tjernbergiae-12	6	3	Acidovorax-14	4	1
Acinetobacter tjernbergiae-14	5	4	Sediminibacterium-10	3	2
Streptococcus-11	4	5	Nitrosospira-14	3	2
Sediminibacterium-12	6	2	Bacillus-10	3	2
Corynebacterium-11	6	2	Acidovorax-12	3	2
Comamonas testosteroni-10	6	2	Streptococcus-10	2	3
Delftia-11	5	3	Stenotrophomonas-10	2	3
Moraxella-11	3	5	Cutibacterium acnes-10	2	3
Corynebacterium-10	3	5	Kocuria-10	2	3
Bradyrhizobium-10	3	5	Streptococcus-9	1	4
Acinetobacter junii-12	3	5	Pseudomonas-8	1	4
Pseudomonas-12	2	6	Pseudomonas-11	1	4
Cloacibacterium-10	2	6	Nitrosospira-11	1	4
Novosphingobium-9	5	2	Lactobacillus-10	1	4
Moraxella-12	5	2	Anaerococcus-8	1	4
Moraxella-9	4	3	Comamonas jiangduensis-13	0	5

Table SF1: Results of object merge transformations.

most frequently in the samples, so we summed these to the species level. Although not as prevalent, we also decided to do the same for *Comamonas* because of its prevalence within the controls. The rest were summed to the genus level. These counts were then normalized to relative abundances within each sample. We did experiment with all genus level summation without much difference in results. The latter three genera are hereafter referred to as the principal bacteria and are sometimes abbreviated, Cu, A and C.

We binned these counts using the logarithmic binning (14 bins from 10^{-5} to 100%) shown in the paper in Table 2, implicitly avoiding the assumption that small abundances were not important. While we did not know if this binning was optimal, we knew from experience that it could reveal microbiome structure, so we began here with the intention of adjusting it to maximize the repeatability of the computations. Discrete measurement objects for each sample were created by concatenating microbe name and bin number (1-14), e.g., *Methylobacterium*-14, as already described. Note that the number of sample objects are not necessarily the same for each sample, nor do we include zero abundance objects.

With this scheme, 83% of the objects occurred 5 times or less and 96% occurred 10 or less times in the data set. *Cutibacterium*, *Acinetobacter* and *Comamonas* species objects made up most of the objects with 10 or more counts. See Table SF1: Results of object merge transformations. The average number of common objects between sample pairs, the overlap, equaled 0.43, an indication of the sparseness of the data.

LDA can perform analyses where there is little overlap between pairs of samples but functions better the more overlap there is, and our initial binning and naming scheme did not decrease sparsity enough. The first runs suggested that the data could support about 5 classes. Since LDA finds within-sample object co-occurrences across many samples, we thought higher overlap would improve repeatability. Doing this objectively required filtering and grouping the data differently, specifically, reducing the microbial name specificity or the abundance resolution or both. We refer to this as object merging since different named and abundance-labeled objects are mapped to the same name and abundance label. In many microbiome studies, this is accomplished by summing higher up the phylogenetic tree, but this is a blunt instrument that would make it more difficult to see the biology if it were there. Our sequencing capability provided sub-species fidelity and we wished to retain as much information as possible. Summing higher in the phylogenetic tree turned out to be unnecessary to achieve repeatability.

Objects contain information about both the bacterium's identity and its abundance in a sample which is a crude way of measuring their behavior, albeit not a detailed molecular biological characterization. When LDA results characterize a sample by class, the suggestion is that two samples in the same color class, even when they do not contain exactly the same bacterial objects, may have similar processes going on within them. In other words, the result is suggesting a commonality that could represent a redundancy of bacterial behavior.

Still, transformations that group different-named bacteria and that reduce abundance resolution could average out the biology. The goal is to transform the data to get reasonable repeatability but not go beyond what is needed. Of course, we did not know a priori whether we had enough data to achieve reproducibility without averaging out the biology nor where the line of optimal binning and naming was. The best approach was to achieve repeatability and hope the results revealed biology.

The next several paragraphs summarize how we arrived at a naming and binning structure that produced repeatable LDA results.

To begin with, we noticed that *Cutibacterium*, *Acinetobacter* and *Comamonas* species objects had class structure (one or two dominant classes) that was apparent from the earliest runs. Individual species of *Cutibacterium* and *Acinetobacter*, particularly C. acnes and A. junii are examples. *Comamonas*, while its species objects have fewer total counts, had high counts per class in one class, increasing its statistical significance. So, we preserved the abundance resolution and naming from our starting point for *Cutibacterium* and *Acinetobacter* and preserved the name for *Comamonas* while reducing the abundance resolution. For other objects, the overall idea was that either by summing over lower abundances or lower occurring objects, we could get enough

statistical significance to see a significant class structure emerge.

Objects with abundances over 12 had class structures where one component dominated the class distribution. Below 12, it was less clear mainly because objects had too few occurrences (counts). There were several high abundance but low count objects that occurred frequently in the magenta and red classes but whose samples jumped from one of these classes to the other with repeated runs, perhaps contributing to the repeatability problem. Last, there were many objects with 5 or less occurrences.

In this scheme to reduce abundance resolution and naming specificity, we never cherry-picked by name or used disease state. We only used count cutoffs and abundance cutoffs applied to the entire data set. Because 12 was the abundance limit above which we could see structure with *Cutibacterium* and *Acinetobacter* we used this cutoff as we gradually realized that two abundance bins was what worked to get repeatability for other objects. We summed over the objects of questionable significance because of counts and filtered out very low count objects. This was not done in one step but through multiple runs of trying to optimize repeatability and graph quality.

It is useful to break the objects into four categories to describe the transformation from the original binning and naming: High-Count-High-Abundance (HiCnt-hi), Low-Count-High-Abundance (LoCnt-hi), High-Count-Low-Abundance (HiCnt-lo), and Low-Count-Low-Abundance (LoCnt-lo). The high-abundance cutoff was 12 or more and the high-count cutoff was about 10. The detailed parameters described below are a little different, but the following summary is easier to understand. Only resultant objects with 5 or more counts were retained. Please note that even when an object is mapped to a new name and abundance, it is still classified and contributes exactly the same to the LDA statistics but from a better-defined object.

HiCnt-hi: Consisted of mainly P and A objects. Kept initial abundance resolution over 12 and retained names.

LoCnt-hi: Consisted of many differently named objects mainly found in samples from red and magenta classes. Renamed to LoCnt. retained abundance 14 label with abundances of 12 or 13 mapped to hi.

HiCnt-lo: There were very few of these objects. Name retained. Abundance mapped to lo.

LoCnt-lo: Many different bacteria. When these were P or A, the name was retained, otherwise the object name was mapped to LoCnt and abundance to lo. 16 of these were kept.

LDA tends to defy standard statistical intuition though. While there may be a tendency to filter out low-occurring (low-count) objects, retaining these objects within a sample can reduce sample class distribution statistical error. This is why a trial-and-error approach, slowly adjusting parameters to get repeatability in terms of sample membership in each color class and cluster was an appropriate way to avoid classification bias.

The results are shown in Table SF1. The specific transformations are detailed in Object Merging: Details below.

Overall, we reduced the number of objects from 218 to 69 with the abundance transformation and then to 52 with the name transformation. With this scheme, 2% of the objects occurred 5 times or less and 40% occurred 10 or less and we were able to increase the overall overlap statistics from 0.43 objects per sample to 0.86 objects per sample. The number of pairs with no overlap went from about 5,000 to 3,500 out of totals of about 7,500 (~120*120/2). Among pairs with non-zero overlap our scheme improved the overlap from 1.30 to 1.63. The three renamed objects had maximum classes as follows: LoCnt-lo was blue and both LoCnt-Hi and LoCnt-14 were magenta. See Table S5. These changes improved the repeatability (detailed definition above) into the 80-90% range.

<u>Object merging: Details</u>. After a great deal of LDA experimentation, we arrived at this scheme as described above.

Identify the following subsets of objects:

Subset 1: Include any *Cutibacterium* (P), *Acinetobacter* (A) or *Comamonas* (C) species and genera from other objects with abundances ≥ 12 and occurring three or more times (specific objects for use in abundance transformation).
Subset 2: Include any species or genera not in subset 1 that occur ≥ 5 times (all objects of specific microbes for use in abundance transformation).
Subset 3: Include objects with counts > 1 and not P or A and £ 4 occurrences - (specific objects for use in count-based name transformation).

Perform the following transformations: for each of the subsets:

1a) For subset 1, P & A species objects remain the same for abundances ≥ 12. All other abundances are mapped to -lo.
1b) For the rest of subset 1, if their abundance is 14, no change. For abundance 12 and 13 objects, the abundances are mapped to -hi and the remaining are mapped to -lo.
1c) For the objects whose microbes are not in subset 1 ≥ 12 is mapped to -hi, £ 11 to -lo.
2) For subset 2, for objects with abundances of 14, the object is not changed; if abundance = 12 or 13, mapped to -hi otherwise it is deleted.
3) Using the results of 1 and 2, for objects in subset 3, names are mapped to LoCnt. resulting in members of subset three being mapped to 'LoCnt-14', 'LoCnt-hi' and 'LoCnt-lo'.

Graphs. The patterns found by LDA are sometimes difficult to understand so we developed graphical visualization techniques to assist us. Below, we include the shorter information from the paper with additional technical details.

<u>Type I Graphs</u>. This type of graph, where the nodes are samples, was designed to display classification results,

10.3389/fcimb.2023.1123228

sample similarity, metadata values and metadata statistics. A glance enables you to get a sense of the quality of the classification and see the presence of statistical fluctuations in the classification, which are the nodes outside the clusters. The graph helps to reveal gross features of the classification which may relate to the emergent features of the microscopic ecosystem biology. The graphs were drawn using Wolfram Mathematica (Wolfram Research Inc., 2010, 2021).

Nodes. Each node represents a sample.

Color. Each node is colored with the sample's color. The LDA computations result in each sample being described by C components, where C is the preset number of classes used by the LDA algorithm. The color of a node should not be confused with an exclusive classification of the node. While each node is, in fact, described by a mixture of C components, the ubiquitous existence of color clusters suggests that the exclusive classification suggested by the colors is an approximation that is justified.

Node Size. Nodes are enlarged if a sample contains one or more specific microbial objects of interest. This visualization is used frequently to explore the class location of objects of the same microbe but differing abundance bin.

Node Shape. The shape of the node displays the SUBJECT metadata value - diamonds for AD, circles for controls. Typically, we may note the diamond fraction statistic next to a color cluster. This AD statistic is the number of diamonds in the cluster divided by the total number of nodes in the color cluster. In our data, we have roughly 50% of the samples from AD subjects and 50% from controls. So, if the class means something for AD, the diamond statistic should be way over 50% if there is a correlation with AD or way less than 50% if the class is anti-correlated with AD. The fact that this is not the case is something we address.

Class Number Optimization. The embedding algorithm helped to optimize the class number input parameter. From experience, we knew that microbiome data formed homogeneously colored clusters mainly because samples tend to be dominated by one class. If the class number is set too high, the graph will display small satellite clusters near the main clusters, often not tightly clustered or repeatable from run to run. If it is set too low, clusters will be formed with samples that are too dissimilar. These are often not tightly clustered and can be multi-colored when two smaller components are merged that sometimes become the dominant class. Proving that tightly clustered, homogeneously colored clusters represent the best classification is beyond the scope of this paper but we will assume it since it helps to make sure that samples that have similar composition end up in the same class. Keep in mind that the disease state is not used in the classification computation. Heterogeneous colored clusters suggest a lack of class dominance within samples and a lack of class structure (non-equal components, one dominating, in particular). Small, rarefied satellite clusters of samples are prone to class hopping between runs and therefore suggest diminished repeatability.

Edge. Edges were defined by node pair similarity. In general, many types of similarities can be used but we used a coarse measure, the dot product. In this case, the similarity is the product of each pair of components summed together. We did not use every possible pair to form an edge. We selected edges in the following way. We formed a histogram of all possible similarities from pairs of nodes, and selected a range from those pairs with the highest similarities on the right tail of the distribution. We found that even when ranges spanned a small piece of this tail, the entire set of nodes was likely to be included among the selected edges. In practice, we varied this range as a kind of focusing mechanism to get a well clustered graph.

Node Position. The features above define the topology of the graph — how the nodes were connected (Wolfram Research Inc., 2010). An embedding algorithm is used to position the nodes in 2D, or 3D space. The algorithm finds the equilibrium position of the nodes when the nodes and edges are given physical properties that both repel and attract the nodes. The repulsion is computed by assuming that each node possesses the same electrical charge, and the attraction derives from representing each edge as a spring. This algorithm is known as spring-electrical embedding (Wolfram Research Inc., 2021) and the resultant graphs are called force-directed graphs. It is possible to have springs whose spring constants are a function of similarity; however, we used a simple binary method. If nodes were connected, they used springs with the same constant, an adjustable parameter. Node clustering is driven by the edge spring. This algorithm positions the nodes in 3D space and the images we present are a projection of the 3D arrangement onto a 2D plane. Because nodes that are the most similar are connected by springs, samples that are the most similar are pulled together in clusters. Nodes that are similar are found near one another and nodes that are not similar are located far away from each other.

Outliers. Typically, nodes that are relatively far away from a cluster compared to other nodes of the same color indicate a statistical fluctuation in the LDA class assignment. What happens is the underlying class distribution has a maximum close to another component which should be the maximum, so it ends up with the 'wrong color' and because it is not similar to the other nodes, it is positioned far away.

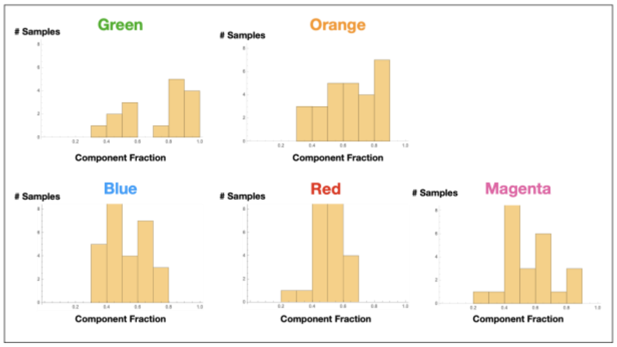

Figure SF3: Sample maximum component distributions by class.

Origin of clusters. A fundamental result of the graph visualization of the MLDA results is the appearance of homogeneously colored clusters. The colors show the maximum MLDA component of the node classification vector. Since there is always a maximum component, we emphasize that the graph embedding algorithm does not just gather the samples with the same maximum component. Only when the MLDA computation has resulted in sample distributions where the maximum component is large, typically over ~0.40 do you get homogeneously colored clusters. In other words, > 40% of the microbial objects have the same classification for each of the samples of a particular color cluster. If the components of the samples were more evenly distributed, where class components were ~0.20, the graph would show a multicolored cluster. Figure SF3 shows that all five classes have samples whose maximum component is $\gtrapprox$ 0.4.

Principal Microbial Object by Class. Below, we describe the principal microbial objects of each class, their class occurrence, average abundances, and the width of the distribution. There are, of course, other objects which can be seen in Table S3, Table S4, Table S5 and Table S7. The dominant bacteria are also made clear in Figure 5.

Green. The most prevalent taxa are the *Comamonas* and *Acinetobacter* species shown in Table 4. Their average abundances are medium, and the width of their abundance distributions is narrow. The abundance of *C. acnes* is low, and its width is wide.

Orange. The most prevalent bacteria are species of the *Acinetobacter* genus. The abundance of the principal *Acinetobacter* species, *A. junii* is high with a narrow distribution width. There was another species of *Acinetobacter, A. tjernbergiae,* with a significant occurrence, but its green-orange dynamic was different from *A. junii* although it was similar. Its involvement, however, in a complex dynamic with *A. junii* is responsible for a significant part of the overlap among samples in the orange class. Orange samples have minimal amounts of *Comamonas* species. The abundance of *C. acnes* is medium with a medium width distribution, occurring in nearly all samples.

Blue. *C. acnes* occurs in almost all samples with a very high abundance and a narrow distribution. *A. junii* is present in low abundances with a wide distribution width. *Comamonas spp.* are not present.

Red. *C. acnes* occurs in all but three samples with somewhat lower abundance than in the blue class but still with a narrow width. The abundance of *A. junii* is present at low abundance with a wide distribution. There is a high occurrence of the M+ genera particularly with abundance levels 13 and 14 and medium distribution width.

Magenta. This class is dominated by the M+ set of genera and they occur in all samples with a medium abundance average and medium width. A significant fraction of the samples has abundance 14 M+ objects. *C. acnes* has a medium abundance and narrow distribution. There is a minimal amount of *A. junii*.

Temporal - Class Relationships Analysis.

Introduction. A strong statistical relationship between pairs of color clusters, that might indicate a temporal relationship, should involve samples that contain microbial objects whose abundances are the same or differ by one. These are situations where it is likely that one microbe is just beginning to outcompete others or the reverse. To visualize this, we constructed graphs where the samples containing objects of neighboring abundances were enlarged. For example, for a microbe m, we might enlarge samples containing m-11 and m-12. These graphs revealed classes that could evolve into one another. When a relationship is present, you see classes, particularly neighboring classes, containing large populations of enlarged nodes.

Another indication of a relationship is when you see a large population of a particular microbe's object (i.e., only one abundance) in two nearby classes. In this case, the actual abundance differences between samples containing the object are not enough to cross the boundary of the logarithmic bin but small changes in the abundances of minor objects are enough to change the class of many samples.

Last, if there were not sufficient data to find large numbers of samples with unit or zero changes in abundance, we constructed the following statistic which has lower abundance resolution (i.e., larger abundance standard deviation). Specifically, we looked at correlations between ranges of abundance greater than 1 for specific bacteria to find these weaker relationships.

Overall, we are looking for a small change or no abundance bin change so that it is sensible to conclude that ecosystems underlying the microbiome of one class could have evolved to another.For shorthand, we will refer to these patterns as the microbe having *pronounced dynamics*.

We show results for the highly occurring species of the *Cutibacterium, Acinetobacter* and *Comamonas* genera as well as the low-count high-abundance objects referred to as M+. These had the most pronounced dynamics. It is difficult to see other less frequently occurring object's populations change as the prevalences are too low to be statistically significant, usually less than 5 samples per class. Thus, while some of these microbes may be important, understanding them will require larger sample sizes.

Class Relationship Details. See Figure 9 and Table 4.

Green-Orange. This pair has *A. junii*-(13-14). In this case, both objects are found in both the green and orange samples. This distribution comprises 10/16 green nodes and 23/27 orange nodes. The dominance of these abundance levels in each class suggests a relationship of green and orange. The presence of a peak of *C. acnes* at 11 in green and 13 in orange is another indicator of a relationship but it is greater than one so we will not focus on it. It is to be noted that this difference

does not seem to be enough to have resulted in a diminishment of the *A. junii* abundances because of the wide bin structure.

Orange-Blue and Green-Blue. These two relationships are a little more difficult to characterize and we will use the wide abundance range statistic for their analyses. By enlarging samples with *A. junii*-(9-13) objects, we see high populations in green, orange, and blue. For the orange-blue, *A. junii* has a peak at 13-14 and blue has a broad wide distribution. The green-blue relationship is similar albeit with a smaller peak at 13-14 in the green. The *C. acnes* abundance also helps clarify these relationships as its average abundance in blue is much larger than in both orange and green. Of these two possible relationships, we are more confident with the orange-blue as the peak for *C. acnes* is 13 in orange and 14 in blue, a change of 1, while there is no pronounced *C. acnes* peak in the green, requiring a very large change to go from green to blue.

Blue-Red. This relationship can be seen in Figure 9 (c) & (d). In (c) there is a commonality between samples with *C. acnes*-14 in blue and the lower part of the red cluster. This is a change of zero. In (d), we show 14-13 together demonstrating a stronger relationship. We will ignore the orange samples that show up for now because it turns out the *C. acnes* goes up and then down by class. The orange samples are on the ascendant part of the curve and the blue, red, and magenta are on the descendant side. We discuss this in more detail below. To drive home this relationship, the *C. acnes* abundance statistics are nearly the same in blue (twenty-seven 14s) and red (fourteen 14s) with a small population of P-13 objects (six 13s). There is an additional correlation provided by *A. junii* that it is widely distributed in both classes, 9-13 in blue and 10-13 in red such that they overlap. This correspondence, however, is weak as there is only one instance where the bin counts are ≥ 5. At any rate, whether we use the vary by zero or one criterion, we can see a strong blue-red relationship.

Red-Magenta. We use three examples to demonstrate this relationship, one with *C. acnes*, one with M+, and the last with *A. junii*. The results are in Figure 9(e) and (f). For *C. acnes* in magenta, there are nine 13s and seven 12s suggesting a strong relationship to red where the abundance level shifts by one or two from the red distribution of 14s and 13s which can be seen in (c) and (d). The red 13s are mainly in the upper part of the red cluster closest to magenta and the magenta 13s are throughout. There are about the same number of instances of *A. junii* over a wide distribution, 4 in red and 5 in magenta. When we use the distribution of M+-(13-14) bacteria in (f) we can see a striking correspondence between the red and magenta *C. acnes* distributions. While the M+ are found in every class, it is only at abundances of 13-14 that a concentration in two classes is seen. Collectively these data support a strong relationship between red and magenta.

Others. We make short arguments for why the following remaining possible pairs should not be included in constructing the time-ordered network. Refer to Table 4.

Green-Red and Green-Magenta. There is a large difference in the average *C. acnes* abundance.There are also extensive differences in the total population of *A. junii* between classes and no *Comamonas spp.* were observed in the red and magenta classes.

Orange-Red. Since orange and blue are related, and blue and red are related, there could be a relationship between orange and red, but it is not as strong as with blue because the *A. junii* differences between orange and red are quite large.

Orange-Magenta. It might seem reasonable to make this argument as the orange *C. acnes* distribution is similar to magenta's, both peaking at 13; however, the *A. junii* connection is not as strong as with blue. Additional arguments come below where we argue that over time, *C. acnes* first rises then falls. The orange 13s can then not be associated with the magenta 13s because orange is on the ascending part of the curve and magenta is on the descending part.

Blue-Magenta. We are going to rule out this relationship as we have already provided ample evidence that red is an intermediate class between blue and magenta. In short, the *C. acnes* abundance is 14 in blue, between 13 and 14 in red, and 13 and under in magenta.

Subject-color logit fit details. The values of coefficients for magenta and green are large and opposite in sign and the others are much smaller, again supporting what can be seen in Figure 11, that the ecosystems underlying all but the magenta class are likely not pathogenic. The coefficients were: (20.0, -3.86, 2.21, -14.9, -31.4) for (green, orange, blue, red, magenta). This raises an issue that will have to be addressed with more research and that is whether the AD statistics for the orange, blue and red classes mainly reflect how large the regions of the AD subject are that are dominated by non-pathogenic ecosystems. A new set of measurements with a similar N could yield quite different AD statistics for the orange, blue and red classes because under-sampled statistics have very large variances.

Supplementary Methods References:

Blei, D. M. (2012). Probabilistic topic models. *Commun. ACM* 55, 77–84. doi: 10.1145/2133806.2133826.

Blei, D. M., Ng, A. Y., and Jordan, M. I. (2003). Latent Dirichlet Allocation. *J. Mach. Learn. Res.* 3, 993–1022.

Gelman, A., and Rubin, D. B. (1992). Inference from Iterative Simulation Using Multiple Sequences. *Stat. Sci.* 7. doi: 10.1214/ss/1177011136.

Geman, S., and Geman, D. (1984). Stochastic relaxation, gibbs distributions, and the bayesian restoration of images. *IEEE Trans. Pattern Anal. Mach. Intell.* 6, 721–41. doi: 10.1109/tpami.1984.4767596.

Griffiths, T. L., and Steyvers, M. (2004). Finding scientific topics. *Proc. Natl. Acad. Sci. U. S. A.* 101 Suppl, 5228–35. doi: 10.1073/pnas.0307752101.

Harrison, J. G., Calder, W. J., Shastry, V., and Buerkle, C. A. (2020). Dirichlet-multinomial modelling outperforms alternatives for analysis of microbiome

and other ecological count data. *Mol. Ecol. Resour.* 20, 481–497. doi: 10.1111/1755-0998.13128.
Heinrich, G. (2008). Parameter estimation for text analysis. Leipzig, Germany Available at: https://www.arbylon.net/publications/text-est2.pdf.
Heinrich, G. (2009). A Generic Approach to Topic Models. in *Proceedings of the 2009th European Conference on Machine Learning and Knowledge Discovery in Databases - Volume Part I* ECMLPKDD'09. (Berlin, Heidelberg: Springer-Verlag), 517–532.
Hofmann, T. (2001). Unsupervised Learning by Probabilistic Latent Semantic Analysis. *Mach. Learn.* 42, 177–196. doi: 10.1023/A:1007617005950.
McInnes, L., Healy, J., Saul, N., and Großberger, L. (2018). UMAP: Uniform Manifold Approximation and Projection. *J. Open Source Softw.* 3, 861. doi: 10.21105/joss.00861.
van der Maaten, L., and Hinton, G. (2008). Visualizing Data using t-SNE. *J. Mach. Learn. Res.* 9, 2579–2605. Available at: http://jmlr.org/papers/v9/vandermaaten08a.html.
Wolfram Research Inc. (2010). Graph. Available at: https://reference.wolfram.com/language/ref/Graph.html [Accessed March 3, 2022].
Wolfram Research Inc. (2021). GraphLayout. Available at: https://reference.wolfram.com/language/ref/GraphLayout.html [Accessed March 3, 2022].
